# Formation of 5-Aminomethyl-2,3-dihydropyridine-4(1*H*)-ones from 4-Amino-tetrahydropyridinylidene Salts

**DOI:** 10.3390/molecules28196869

**Published:** 2023-09-29

**Authors:** Werner Seebacher, Michael Hoffelner, Ferdinand Belaj, Teresa Pirker, Muaaz Alajlani, Rudolf Bauer, Eva-Maria Pferschy-Wenzig, Robert Saf, Robert Weis

**Affiliations:** 1 Pharmaceutical Chemistry, Institute of Pharmaceutical Sciences, University of Graz, Schubertstrasse 1, 8010 Graz, Austria; 2 Institute of Chemistry, University of Graz, Schubertstrasse 1, 8010 Graz, Austria; 3 Pharmacognosy, Institute for Pharmaceutical Sciences, University of Graz, Beethovenstrasse 8, 8010 Graz, Austriarudolf.bauer@uni-graz.at (R.B.);; 4 Faculty of Pharmacy, Al-Sham Private University, 011 Damascus, Syria; 5 Institute for Chemistry and Technology of Materials (ICTM), Graz University of Technology, Stremayrgasse 9, 8010 Graz, Austria

**Keywords:** antibacterial, anticancer activity, dihydropyridin-4(1*H*)-ones, tetrahydropyridinylidene salts

## Abstract

Various 4-aminotetrahydropyridinylidene salts were treated with aldehydes in an alkaline medium. Their conversion to 5-substituted β-hydroxyketones in a one-step reaction succeeded only with an aliphatic aldehyde. Instead, aromatic aldehydes gave 5-substituted β-aminoketones or a single δ-diketone. The new compounds were characterized using spectroscopic methods and a single crystal structure analysis. Some of them showed anticancer and antibacterial properties.

## 1. Introduction

**Scheme 1:**
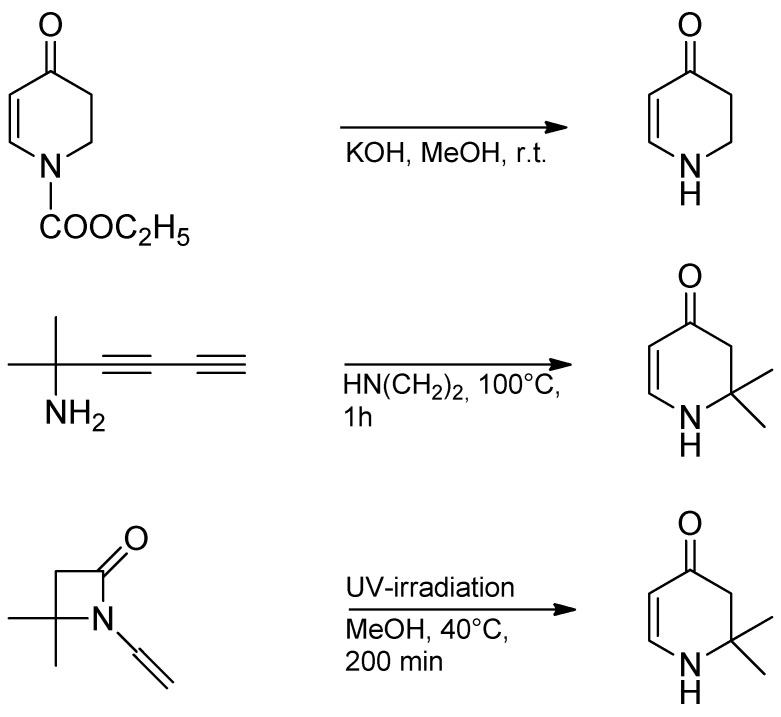
Syntheses of 2,3-dihydropyridin-4(1*H*)-ones [12,13,14].

Recently, we described the synthesis and antiprotozoal potency of 4-aminotetrahydropyridinylidene salts [1] and of 1-benzyl derivatives of these salts with enhanced antiprotozoal activities [2,3]. The insertion of an additional benzyl group in ring position 3 via alkylation under basic conditions yielded 1,3-disubstituted compounds with increased antiplasmodial activity [4]. This paper reports the synthesis of 5-substituted derivatives. Surprisingly, the reaction afforded mainly β-aminoketones, less frequently δ-diketones or the expected β-hydroxyketones, which are usually obtained from α,β-unsaturated ketones under Morita–Baylis–Hillmann conditions [5,6,7]. Similar Mannich-like products were obtained from formaldehyde and secondary amines with quinolin-4(1*H*)-ones [8,9] or pyridin-4(1*H*)-ones [10,11]. However, their synthesis from 4-amino-tetrahydropyridinylidene salts or from their hydrolysis products, the 2,3-dihydropyridin-4(1*H*)-ones, have not yet been reported. Unsubstituted 2,3-dihydropyridin-4(1*H*)-one has been prepared from ethyl 4-oxo-1,4-dihydropyridin-1-carboxylate [12]. Its 2,2-dimethyl analog has been synthesized from 2-methylhexa-3,5-diyn-2-amine [13] as well as from 1-ethenyl-4,4-dimethylazetidin-2-one via UV-irradiation [14] (Scheme 1). Further cyclic enaminones have been used as convenient synthons for numerous reactions including photocycloadditions [15,16,17,18,19] and alkaloid synthesis [20]. The structural and electronic properties of a series of 2,3-dihydropyridin-4-ones have been the subject of a theoretical study [21]. So far, only the insertion of an allyl or an alkenyl substituent in ring position 5 of a dihydropyridine-4-one has been reported [22,23].

**Figure 1:**
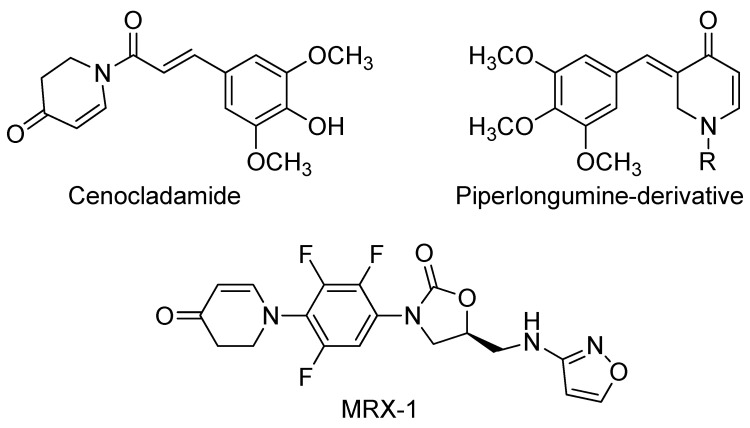
Compounds containing the 2,3-dihydropyridin-4(1*H*)-one motif [24,25,26,27].

2,3-Dihydropyridin-4(1*H*)-one is a partial structure of cenocladamide, an alkaloid from *Piper cenocladum* [24]. This alkaloid and derivatives thereof have previously been investigated for their anticancer potency [25]. Furthermore, the dihydropyridin-4(1*H*)-one moiety is part of derivatives of piperlongumine, which have been investigated for their anticancer activity [26] as well as of the potent antibacterial MRX-I [27] (Figure 1). Therefore, we investigated the new compounds for their anticancer activities as well as their antibacterial potency in vitro.

## 2. Results and Discussion

### 2.1. Chemistry

**Scheme 2:**
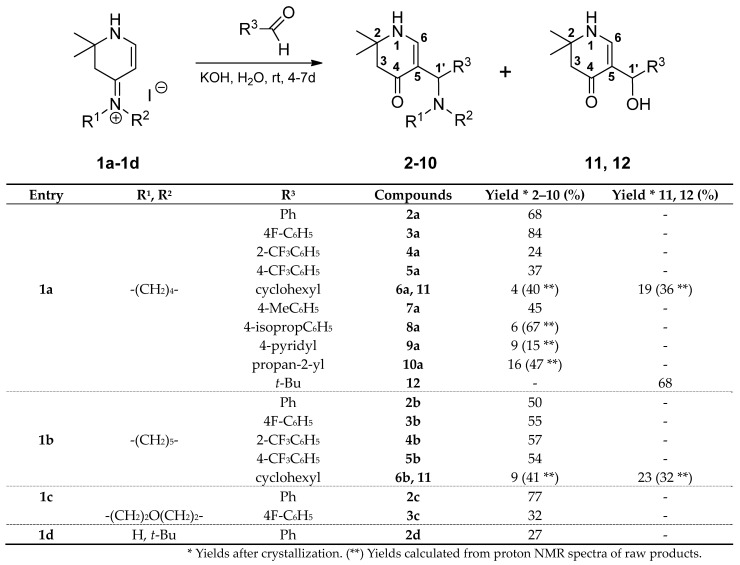
Synthesis of compounds **2–12**.

The different 4-dialkylaminotetrahydropyridinylidene salts **1a–1d** used as starting products were prepared as described earlier [1]. They were exposed for several days to aromatic aldehydes or aliphatic aldehydes in an alkaline medium at room temperature. Hydrolysis of **1a–1d** in ring position 4 was taken into account, but the expected β-hydroxyketones **11** and **12** were only formed as the main product from the aliphatic cyclohexane carbaldehyde and pivalaldehyde, whereas the β-aminoketones **6a** and **6b** were isolated as by-products. The reaction of salts **1a–1d** with aromatic aldehydes afforded mainly β-aminoketones (compounds **2**–**5** and **7–10**) (Scheme 2).

**Scheme 3:**
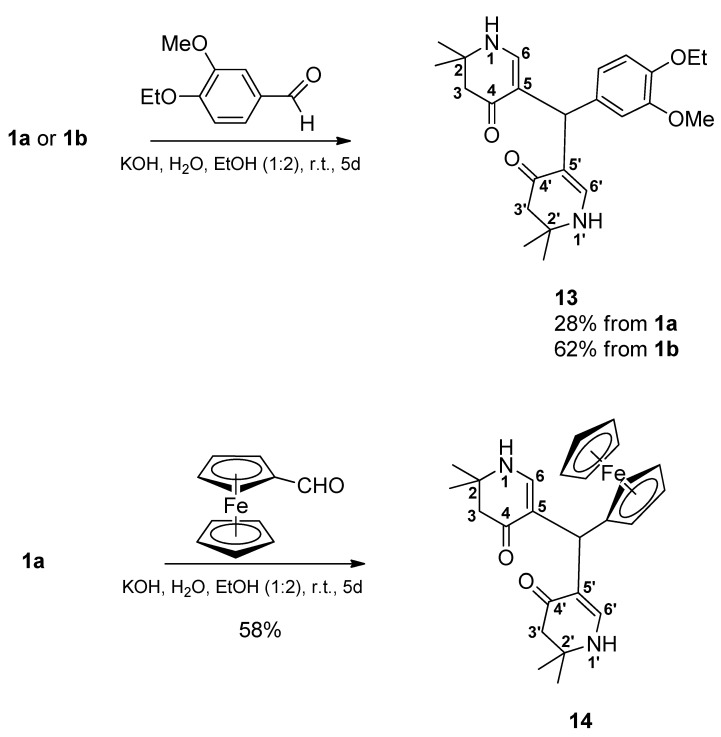
Synthesis of δ-diketones **13** and **14** with electron-sufficient aldehydes.

Due to the low solubility in water of solid ferrocenyl carbaldehyde and 4-ethoxy-3-methoxybenzaldehyde, ethanol was added to the reaction mixture. Other than the so far reported reactions, these electron-sufficient aldehydes resulted in dimers **13** and **14** (Scheme 3).

**Scheme 4:**
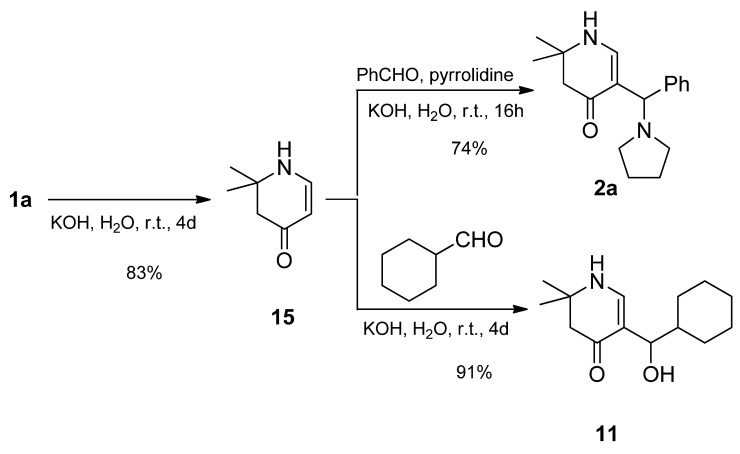
Control experiments with dihydropyridone **15**.

In order to confirm the initial steps of the proposed mechanism, we treated the 4-dialkylaminotetrahydropyridinylidene salt **1a** with potassium hydroxide for 4 days to yield dihydropyridone **15**, quantitatively. Then, a mixture of pyrrolidine and benzaldehyde in alkaline solution was added and considerable amounts of **2a** were formed overnight. The reaction of **15** without secondary amine resulted in a high yield of the hydroxy analog **11** (Scheme 4).

**Scheme 5:**
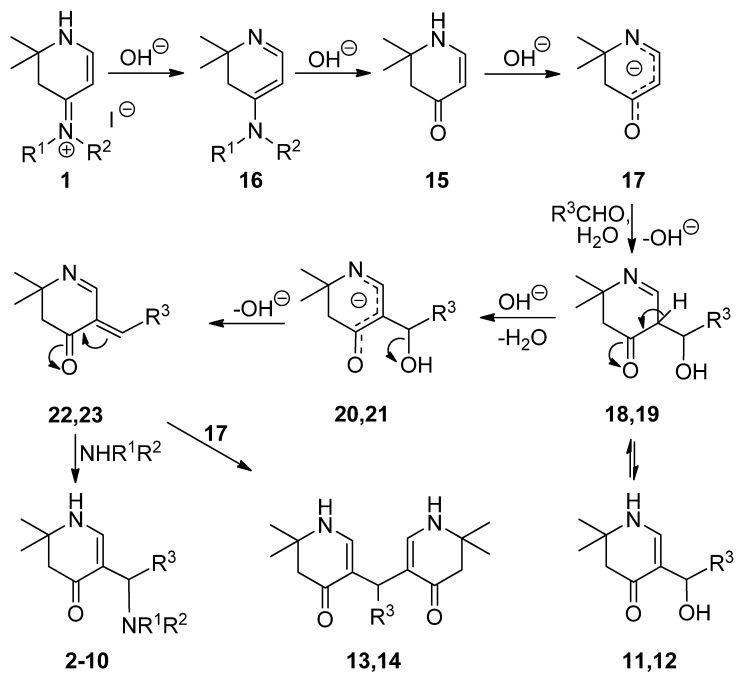
Possible mechanisms of the formation of compounds **2**–**14**.

The reaction mechanism can be considered as follows: The 4-dialkylaminotetrahydropyridinylidene salts **1** are deprotonated by potassium hydroxide to their corresponding bases **16** in the first step. Due to strong alkaline conditions, these are hydrolyzed to ketone **15** which is deprotonated to the resonance-stabilized anion **17**. The formed methylene component is readily attacked by aldehydes resulting in aldols **18**,**19**. If R^3^ is an aliphatic group, tautomerization to compounds **11**,**12** may occur. Moreover, a proton can be abstracted from the α-position of aldols **18**,**19**. Cleavage of a hydroxy group from the formed resonance-stabilized anions **20**,**21** leads to α,β-unsaturated ketones **22**,**23**. Addition of secondary amines in β-position yields β-aminoketones **2–10**. This is the main reaction for the less electrophilic aromatic aldehydes as well as isobutyric aldehydes. Alternatively, compounds **22,23** can be attacked by anion **17** resulting in δ-diketones **13**, **14** via a Michael reaction (Scheme 5).

### 2.2. Structure Elucidation

**Figure 2:**
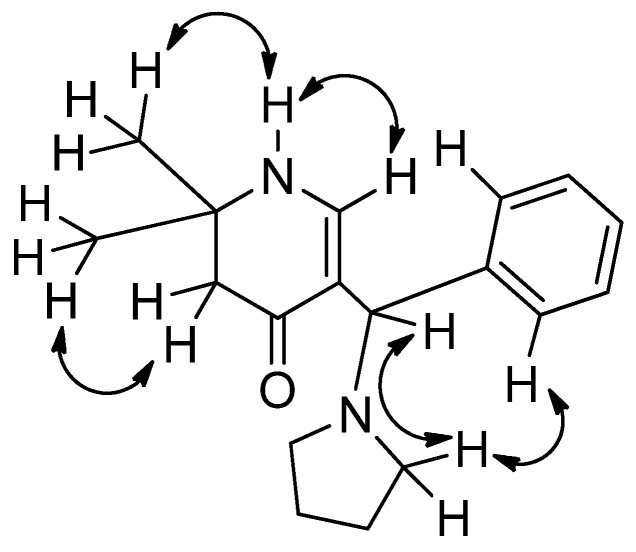
Through-space couplings indicated as arrows in compound **2a**.

The structures of the obtained compounds were elucidated using NMR spectroscopy: In the ^13^C-spectra, a signal shift from 162 to 189 ppm was observed for C-4 due to ketone formation. Furthermore, the resonance of the proton in position 5 disappeared in the ^1^H spectra. The remaining olefinic proton showed a coupling to the proton in ring position 1. Connectivity was proven by cross peaks of the newly formed methine proton to C-4 and C-5 as well as to NCH_2_ and aromatic carbons in the HMBC spectra. Through-space couplings were detected in NOE experiments (Nuclear Overhauser experiments) (Figure 2). Both can be seen in the Supplementary Materials.

**Figure 3:**
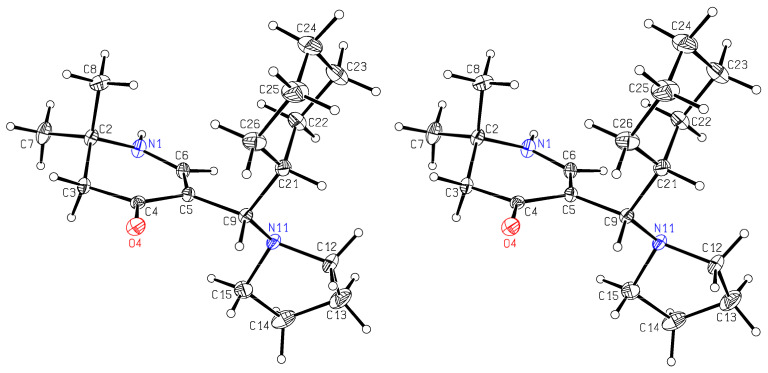
Stereoscopic ORTEP [28] plot of **6a** showing the atomic numbering scheme. The probability ellipsoids are drawn at the 50% probability level. The H atoms are drawn with arbitrary radii. Nitrogen atoms are blue, oxygen atoms are red.

Finally, evidence of the structure of compound **6a** was achieved using a single X-ray crystal analysis which confirmed the compound as 5-[cyclohexyl(pyrrolidin-1-yl)methyl]-2,2-dimethyl-2,3-dihydropyridin-4(1*H*)-one. This is the first determination of a structure containing a (pyrrolidin-1-yl) substituent in a 2,3-dihydropyridin-4(1*H*)-one (Figure 3). All atoms lie on general positions. The compound is a racemate.

### 2.3. Biological Activities

**Figure 4:**
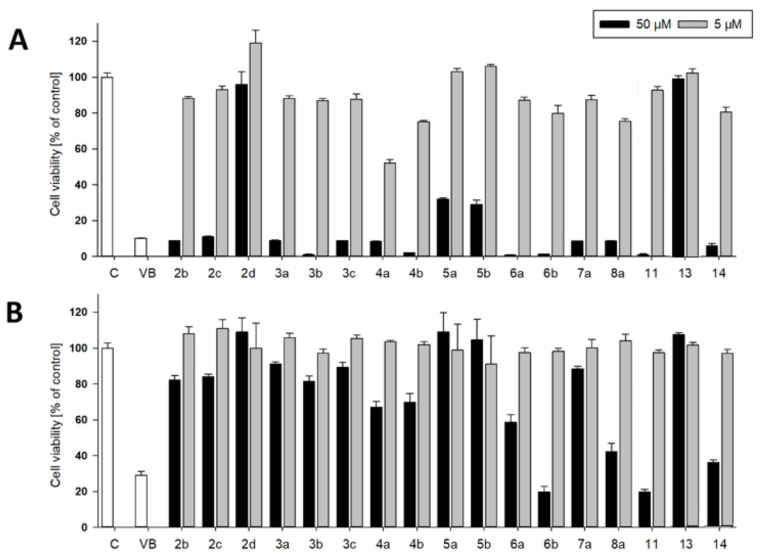
Anti-proliferative activity of compounds **2b–14** against human leukemia cell line CCRF-CEM (**A**) and against non-tumorigenic MRC-5 lung fibroblasts (**B**) expressed as mean ± SEM. Cells were treated with 50 and 5 µM of the compounds for 72 h. Cell viability was measured via XTT assay. Viability rates were expressed as a percentage of vehicle-treated control cells (0.1% DMSO). Vinblastine (VB) at a concentration of 100 nM served as positive control.

Most of the new compounds were tested for their anti-proliferative activity against human leukemia cells (CCRF-CEM) as well as against non-tumorigenic human lung fibroblasts (MRC-5) using an XTT assay. Cells were exposed to compounds at concentrations of 5 and 50 µM for a time period of 72 h. The results are presented in Figure 4A,B.

At concentrations of 5 µM, only the 2-(trifluoromethyl)phenyl aminoketone **4a** showed moderate activity against a leukemia cell line. All other test compounds were inactive at this concentration. At 50 µM, the (*tert*-butylamino)ketone **2d** as well as the dimer **11** were still non-effective and the 4-(trifluoromethyl)phenyl aminoketones **5a** and **5b** had moderate activity. All other compounds showed a selective anti-leukemic effect. However, the aminoketones **6b** and **8a**, dimer **12,** and the β-hydroxyketone **13** were comparably toxic against lung fibroblasts. The most promising selectivity was observed for the 2-(trifluoromethyl)phenyl aminoketone **4a**, which showed good anti-leukemic effect paired with low cytotoxicity in fibroblasts at 5 µM.

The results of the antibacterial assay against Gram-negative (*E. coli*) and Gram-positive bacteria (*B. sub.*) are listed in Table 1.

The most active compounds against *Escherichia coli* were aminoketones **3a**, **4b**, **5a**, and **5b** with fluorophenyl or (trifluoromethyl)phenyl substitution as well as their cyclohexyl analog **6a**. The most active compound against *Bacillus subtilis* was the β-hydroxyketone **11**.

## 3. Materials and Methods

### 3.1. Instrumentation and Chemicals

Solvents were used without further purification. Aldehydes were purified via chromatographic separation on a small column filled with aluminum oxide 60 active basic (activity I) (Merck, Darmstadt, Germany) to remove acidic impurities. For thin-layer chromatography (TLC), TLC plates with silica gel 60 F254 (Merck, Darmstadt, Germany) were used. Melting points were obtained on an Electrothermal IA 9200 melting point apparatus. IR spectra: Bruker Alpha Platinum ATR FTIR spectrometer (KBr discs); frequencies are reported in cm^−1^. The structures of all newly synthesized compounds were determined using one- and two-dimensional NMR spectroscopy. The NMR spectra were acquired with Varian UnityInova 400 (298 K) or Bruker Avance Neo 400 instruments in 5 mm tubes. Some spectra were acquired in CDCl_3_ containing 0.03% TMS. Chemical shifts were recorded in parts per million (ppm). For the ^1^H spectra, TMS (0.00) was used as the internal standard, and for the ^13^C spectra, the central peak of the CDCl_3_ peak was used as the internal reference (77.0). Most of the spectra were acquired in DMSO-d_6_. In this case, the central peaks of the solvent signal at 2.49 ppm in the ^1^H spectra and at 39.7 ppm in the ^13^C spectra served as the internal references. Shifts in the ^1^H NMR (400 MHz) and the ^13^C NMR (100 MHz) spectra are reported in ppm; ^1^H- and ^13^C-resonances were assigned using ^1^H,^1^H- and ^1^H,^13^C-correlation spectra and are numbered as given in Scheme 1. Abbreviations: aromatic H, ArH; aromatic C, ArC; and quaternary aromatic C, ArC_q_. Signal multiplicities are abbreviated as follows: s, singlet; d, doublet; dd, double doublet; dsept, double septet; quin, quintet; t, triplet; m, multiplet; and br, broad. Coupling constants (*J*) are reported in Hertz (Hz). Assignments marked with an asterisk are interchangeable. HRMS: Micromass Tofspec 3E spectrometer (MALDI) and GCT Premier (Waters, Milford, MA, USA) (EI, 70 eV) or Q Exactive Hybrid Quadrupole-Orbitrap mass spectrometer, Thermo Fisher Scientific, Austin, TX, USA (HESI, capillary voltage 3.5 kV). Electron ionization (EI+, 70 eV, source at 250 °C) mass spectra were acquired on a JMS-T2000GC (AccuTOF^TM^ GC-Alpha) from JEOL Ltd. (Tokyo, Japan) equipped with a direct insertion probe (DIP). The ^1^H NMR and ^13^C NMR spectra of new compounds are provided in the Supplementary Materials.

### 3.2. Syntheses

Compounds **1a**–**1d** were prepared earlier and their NMR data are in accordance with the reported literature [1].

*rac*-2,2-Dimethyl-5-[(*R*)-phenyl(pyrrolidin-1-yl)methyl]-2,3-dihydropyridin-4(1*H*)-one (**2a**): Compound **1a** (2.000 g (6.53 mmol)) was suspended in water (16 mL) and a solution of KOH (1.466 g (26.13 mmol)) in water (16 mL) was added. The mixture was stirred at r.t. until a solution was formed (ca. 5–10 min). Then, benzaldehyde (0.693 g (6.53 mmol)) was added. The reaction mixture was stirred for 4 days at r.t. The separated crystalline solid was filtered with suction, washed with water and acetone, and dried in vacuo yielding **2a** (1.261g (68%)) as an orange-yellow solid. For analytical purposes, it was recrystallized from ethyl acetate/cyclohexane resulting in a white solid.

From **15**: Compound **15** (0.671 g (5.36 mmol)) was dissolved in a solution of KOH (1.213 g (21.62 mmol)) in distilled water (26 mL). The mixture was sonicated and stirred at r.t. until all was dissolved. To the resulting solution, pyrrolidine (0.409 g (5.75 mmol)) and benzaldehyde (0.582 g (5.48 mmol)) were added. The reaction mixture was stirred for 16 h at r.t. The separated crystalline solid was filtered with suction, washed clean with water, and dried in vacuo resulting in **2a** (1.129 g (74%)) as a pale pink solid. For analytical purposes, it was recrystallized from ethyl acetate resulting in colorless needles.

*R*_f_ (CH_2_Cl_2_:MeOH = 10:1): 0.09; mp: 147–148 °C; IR = 3232, 2967, 2783, 1623, 1568, 1534, 1395, 1231, 700; ^1^H NMR (CDCl_3_, 400 MHz) *δ* = 1.19 (s, 3H, CH_3_), 1.29 (s, 3H, CH_3_), 1.75 (br, s, 4H, (CH_2_)_2_), 2.37 (s, 2H, 3-H), 2.50 (br, s, 4H, 2NCH_2_), 4.52 (s, 1H, 1′-H), 4.60 (d, *J* = 5.5 Hz, 1H, 1-H), 7.14–7.38 (m, 6H, 6-H, ArH); ^13^C NMR (CDCl_3_, 100 MHz) *δ* = 23.49 ((CH_2_)_2_), 26.57, 27.57 (2CH_3_), 49.18 (C-3), 53.17 (2NCH_2_), 53.80 (C-2), 63.98 (C-1′), 111.43 (C-5), 126.09, 127.14, 128.03 (5ArC), 144.87 (ArC_q_), 149.23 (C-6), 189.50 (C-4); HRMS (EI): calcd. (C_18_H_24_N_2_O^+^) [M]^+^: 284.1889; found: 284.1874.

*rac*-2,2-Dimethyl-5-[(*R*)-phenyl(piperidin-1-yl)methyl]-2,3-dihydropyridin-4(1*H*)-one (**2b**): Compound **1b** (2.091 g (6.53 mmol)) was suspended in water (16 mL) and a solution of KOH (1.470 g (26.20 mmol)) in water (16 mL) was added. The mixture was stirred at r.t. until a solution was formed (ca. 10–20 min). Then, benzaldehyde (0.695 g (6.55 mmol)) was added. The reaction mixture was stirred for 5 days at r.t. The separated crystalline solid was filtered with suction, washed with water and acetone, and dried in vacuo resulting in **2b** (0.972 g (50%)) as an orange-yellow solid. For analytical purposes, it was recrystallized from ethyl acetate resulting in a white solid. *R*_f_ (CH_2_Cl_2_:MeOH = 1:1): 0.86; mp: 158 °C; IR = 2925, 1623, 1571, 1527, 1255, 1232, 700; ^1^H NMR (DMSO-d_6_, 400 MHz) *δ* = 1.09 (s, 3H, CH_3_), 1.18 (s, 3H, CH_3_), 1.31–1.51 (m, 6H, (CH_2_)_3_), 2.13–2.22 (m, 4H, 3-H, NCH_2_), 2.28–2.38 (m, 2H, NCH_2_), 4.37 (s, 1H, 1′-H), 6.95 (d, *J* = 6.8 Hz, 1H, 6-H), 7.09–7.14 (m, 1H, ArH), 7.20–7.26 (m, 4H, ArH), 7.35 (d, *J* = 6.8 Hz, 1H, 1-H); ^13^C NMR (DMSO-d_6_, 100 MHz) *δ* 24.62 (CH_2_), 26.11 (2CH_2_), 26.19, 26.78 (2CH_3_), 48.90 (C-3), 52.34 (2NCH_2_), 53.03 (C-2), 64.17 (C-1′), 106.75 (C-5), 125.98, 127.30, 128.17 (5ArC), 144.62 (ArC_q_), 149.38 (C-6), 188.49 (C-4); HRMS (HESI): calcd. (C_19_H_27_N_2_O^+^) [M + H]^+^: 299.2123; found: 299.2114.

*rac*-2,2-Dimethyl-5-[(*R*)-phenyl(morpholin-4-yl)methyl]-2,3-dihydropyridin-4(1*H*)-one (**2c**): Compound **1c** (2.000 g (6.22 mmol)) was suspended in water (30 mL) and KOH (1.397 g (24.89 mmol)) was added. To the resulting yellow solution benzaldehyde (0.660 g (6.22 mmol)) was added. The reaction mixture was stirred for 4 days at r.t. The separated crystalline solid was filtered with suction, washed with water and acetone, and dried over phosphorus pentoxide in vacuo resulting in **2c** (1.436 g (77%)) as an off-white solid. For analytical purposes, it was recrystallized from ethyl acetate resulting in white needles. *R*_f_ (CH_2_Cl_2_:MeOH = 1:1): 0.89; mp: 165 °C; IR = 3257, 2961, 1623, 1571, 1522, 1395, 1291, 1231, 1181, 1116, 702; ^1^H NMR (DMSO-d_6_, 400 MHz) *δ* 1.09 (s, 3H, CH_3_), 1.17 (s, 3H, CH_3_), 2.15–2.25 (m, 4H, 3-H, NCH_2_), 2.29–2.39 (s, 2H, NCH_2_), 3.49–3.60 (br, s, 4H, 2OCH_2_), 4.36 (s, 1H, 1′-H), 7.00 (d, *J* = 6.8 Hz, 1H, 6-H), 7.13–7.16 (m, 1H, ArH), 7.25–2.26 (m, 4H, ArH), 7.42 (d, *J* = 6.8 Hz, 1H, 1-H); ^13^C NMR (DMSO-d_6_, 100 MHz) *δ* 26.20, 26.77 (2CH_3_), 48.82 (C-3), 52.11 (2NCH_2_), 53.04 (C-2), 64.25 (C-1′), 66.61 (2OCH_2_), 106.04 (C-5), 126.26, 127.42, 128.33 (5ArC), 143.71 (ArC_q_), 149.51 (C-6), 188.47 (C-4); HRMS (HESI): calcd. (C_14_H_16_ON^+^) [M + H − C_4_H_9_NO]^+^: 214.1232; found: 214.1223.

*rac*-5-[(*R*)-(*tert*-Butylamino)(phenyl)methyl]-2,2-dimethyl-2,3-dihydropyridin-4(1*H*)-one (**2d**): Compound **1d** (2.013 g (6.53 mmol)) was suspended in water (30 mL) and KOH (1.400 g (25 mmol)) was added. Then, benzaldehyde (0.693 g (6.53 mmol)) was added and the reaction mixture was stirred for 6 days at r.t. From the resulting orange resin, the aqueous solution was decanted and the resin was washed with water repeatedly. Then, it was dried overnight in vacuo over phosphorus pentoxide. The dry resin was dissolved in the minimum amount of hot ethyl acetate and left for crystallization at r.t. The solid was sucked off and dried resulting in **2d** (0.512 g (27%)) as off-white needles. *R*_f_ (MeOH): 0.07; mp: 136 °C; IR = 2964, 1633, 1550, 1477, 1454, 1412, 1367, 1357, 1195, 1174, 947, 875, 828, 727, 713, 697, 678; ^1^H NMR (DMSO-d_6_, 400 MHz) *δ* 1.07 (s, 3H, CH_3_), 1.10 (s, 3H, CH_3_), 1.20 (s, 9H 3CH_3_), 2.31 (d, *J* = 15.0 Hz, 1H, 3-H), 2.36 (d, *J* = 15.0 Hz, 1H, 3-H), 5.21 (s, 1H, 1′-H), 5.98 (br, s, 1H, NH*), 6.25 (br, s, 1H, 6-H*), 6.94 (br, s, 1H, 1-H*), 7.12–7.34 (m, 5H, ArH); ^13^C NMR (DMSO-d_6_, 100 MHz) *δ* 26.64, 26.81 (2CH_3_), 31.66 (3CH_3_), 41.37 (C-3), 50.97 (C-2), 53.45 (*C*(CH_3_)_3_), 73.61 (C-1′), 109.18 (C-5), 126.17, 126.43, 127.59 (5ArC), 138.93 (C-6), 146.02 (ArC_q_), 161.70 (C-4); HRMS (HESI): calcd. (C_18_H_27_N_2_O^+^) [M + H]^+^: 287.2123; found: 287.2114.

*rac*-5-[(*R*)-(4-Fluorophenyl)(pyrrolidin-1-yl)methyl]-2,2-dimethyl-2,3-dihydropyridin-4(1*H*)-one (**3a**): Compound **1a** (2.000 g (6.53 mmol)) was suspended in water (16 mL) and a solution of KOH (1.466 g (26.13 mmol)) in water (16 mL) was added. The mixture was stirred at r.t. until a solution was formed (ca. 5–10 min). Then, 4-fluorobenzaldehyde (0.810 g (6.53 mmol)) was added and the reaction mixture was stirred for 6 days at r.t. The separated crystalline solid was filtered with suction, washed with water, and dried in vacuo over phosphorus pentoxide resulting in **3a** (1.658 g (84%)) as a beige solid. It was recrystallized from ethyl acetate resulting in white needles. *R*_f_ (CH_2_Cl_2_:MeOH = 1:1): 0.29; mp: 169 °C; IR = 3231, 2970, 2783, 1623, 1603, 1568, 1531, 1507, 1395, 1220, 1182; ^1^H NMR (DMSO-d_6_, 400 MHz) *δ* 1.07 (s, 3H, CH_3_), 1.16 (s, 3H, CH_3_), 1.62–1.65 (m, 4H, (CH_2_)_2_), 2.14 (s, 2H, 3-H), 2.25–2.39 (m, 2H, NCH_2_), 2.33–2.39 (m, 2H, NCH_2_), 4.29 (s, 1H, 1′-H), 7.00–7.05 (m, 2H, ArH), 7.10 (d, *J* = 6.8 Hz, 1H, 6-H), 7.25–7.29 (m, 2H, ArH), 7.36 (d, *J* = 6.8 Hz, 1H, 1-H); ^13^C NMR (DMSO-d_6_, 100 MHz) *δ* 23.34 ((CH_2_)_2_), 26.02, 26.85 (2CH_3_), 48.92 (C-3), 52.79 (2NCH_2_), 53.03 (C-2), 63.35 (C-1′), 108.89 (C-5), 114.74 (d, ^2^*J*(C,F) = 21.0 Hz, ArC), 128.66 (d, ^3^*J*(C,F) = 7.9 Hz, ArC), 141.72 (d, ^4^*J*(C,F) = 3.1 Hz, ArC_q_), 149.08 (C-6), 160.69 (d, ^1^*J*(C,F) = 241.3 Hz, ArC_q_), 187.86 (C-4); HRMS (HESI): calcd. (C_18_H_24_FN_2_O^+^) [M + H]^+^: 303.1873; found: 303.1863.

*rac*-5-[(*R*)-(4-Fluorophenyl)(piperidin-1-yl)methyl]-2,2-dimethyl-2,3-dihydropyridin-4(1*H*)-one (**3b**): Compound **1b** (2.091 g (6.53 mmol)) was suspended in water (30 mL) and KOH (1.470 g (26.20 mmol)) was added. Then, 4-fluorobenzaldehyde (0.810 g (6.53 mmol)) was added and the reaction mixture was stirred for 4 days at r.t. The separated crystalline solid was filtered with suction, washed with water, and dried in vacuo over phosphorus pentoxide yielding **3b** as a yellowish solid. It was dissolved in hot ethyl acetate and the insoluble part was removed via filtration. The product crystallized overnight as needles, was sucked off, and washed with ice-cold ethyl acetate resulting in **3b** (1.137 g (55%)) as yellowish needles. *R*_f_ (CH_2_Cl_2_:MeOH = 1:1): 0.30; mp: 183 °C; IR = 3250, 3031, 2965, 2938, 1624, 1571, 1525, 1506, 1393, 1380, 1290, 1255, 1231, 1220, 1180; ^1^H NMR (DMSO-d_6_, 400 MHz) *δ* 1.10 (s, 3H, CH_3_), 1.18 (s, 3H, CH_3_), 1.34–1.48 (m, 6H, (CH_2_)_3_), 2.14–2.23 (m, 4H, 3-H, NCH_2_), 2.26–2.36 (m, 2H, NCH_2_), 4.37 (s, 1H, 1′-H), 6.95 (d, *J* = 6.8 Hz, 1H, 6-H), 7.05 (t, *J* = 8.9 Hz, 2H, ArH), 7.25 (dd, *J* = 8.5, 5.8 Hz, 2H, ArH), 7.38 (d, *J* = 6.8 Hz, 1H, 1-H); ^13^C NMR (DMSO-d_6_, 100 MHz) *δ* = 24.56 (CH_2_), 26.09 (2CH_2_), 26.15, 26.72 (2CH_3_), 48.88 (C-3), 52.22 (2NCH_2_), 53.02 (C-2), 63.60 (C-1′), 106.41 (C-5), 114.78 (d, ^2^*J*(C,F) = 21.0 Hz, ArC), 128.96 (d, ^3^*J*(C,F) = 7.8 Hz, ArC), 140.55 (d, ^4^*J*(C,F) = 3.0 Hz, ArC_q_), 149.31 (C-6), 160.66 (d, ^1^*J*(C,F) = 241.6 Hz, ArC_q_), 188.51 (C-4); HRMS (HESI): calcd. (C_19_H_26_FN_2_O^+^) [M + H]^+^: 317.2029; found: 317.2020.

*rac*-5-[(*R*)-4-Fluorophenyl)(morpholin-4-yl)methyl]-2,2-dimethyl-2,3-dihydropyridin-4(1*H*)-one (**3c**): Compound **1c** (0.700 g (2.18 mmol)) was suspended in water (11 mL) and KOH (0.489 g (8.71 mmol)) was added. 4-Fluorobenzaldehyde (0.271 g (2.18 mmol)) was added and the reaction mixture was stirred for 6 days at r.t. The separated resin was washed with water and dried in vacuo. It was triturated with hot ethyl acetate and the insoluble parts were filtered off and a part of the solvent was removed via evaporation. The formed amorphous precipitate was filtered off and the filtrate concentrated in vacuo. The product crystallized and was sucked off, washed with cold ethyl acetate, and dried yielding **3c** (0.225 g (32%)). *R*_f_ (CH_2_Cl_2_:MeOH = 1:1): 0.90; mp: 162 °C; IR = 3244, 2969, 2957, 2842, 2809, 1651, 1621, 1571, 1508, 1394, 1292, 1263, 1249, 1224, 1182, 1118; ^1^H NMR (DMSO-d_6_, 400 MHz) *δ* = 1.09 (s, 3H, CH_3_), 1.17 (s, 3H, CH_3_), 2.14–2.23 (m, 4H, 3-H, NCH_2_), 2.30–2.39 (m, 2H, NCH_2_), 3.51–3.56 (m, 4H, 2OCH_2_), 4.35 (s, 1H, 1′-H), 7.00 (d, *J* = 6.8 Hz, 1H, 6-H), 7.07 (t, *J* = 8.8 Hz, 2H, ArH), 7.27 (dd, *J* = 8.5, 5.8 Hz, 2H, ArH), 7.45 (d, *J* = 6.8 Hz, 1H, 1-H); ^13^C NMR (DMSO-d_6_, 100 MHz) *δ* = 26.18, 26.73 (2CH_3_), 48.82 (C-3), 52.02 (2NCH_2_), 53.05 (C-2), 63.72 (C-1′), 66.60 (2OCH_2_), 105.78 (C-5), 114.99 (d, ^2^*J*(C,F) = 21.0 Hz, ArC), 129.15 (d, ^3^*J*(C,F) = 7.7 Hz, ArC), 139.72 (d, ^4^*J*(C,F) = 3.0 Hz, ArC_q_), 149.46 (C-6), 160.81 (d, ^1^*J*(C,F) = 241.8 Hz, ArC_q_), 188.51 (C-4); HRMS (HESI): calcd. (C_14_H_15_FNO^+^) [M + H − C_4_H_9_NO]^+^: 232.1138; found: 232.1129.

*rac*-2,2-Dimethyl-5-{(*R*)-(pyrrolidin-1-yl)[(2-trifluoromethyl)phenyl]methyl}-2,3-dihydropyridin-4(1*H*)-one (**4a**): Compound **1a** (1.000 g (3.27 mmol)) was suspended in water (16 mL) and KOH (0.733 g (13.1 mmol)) was added. The mixture was stirred at r.t. until a solution was formed (ca. 5–10 min). Then, 2-(trifluoromethyl)benzaldehyde (0.569 g (3.27 mmol)) was added and the reaction mixture was stirred for 4 days at r.t. The separated resinous solid was washed with water and dried in vacuo over phosphorus pentoxide. It was dissolved in hot ethyl acetate and cyclohexane was added until the first turbidity appeared. An oil separated which solidified. This amorphous precipitate was filtered off and discarded. The filtrate was evaporated and dissolved in hot ethyl acetate. It was allowed to stand for some days until crystallization took place resulting in **4a** (0.282 g (24%)) as white needles. *R*_f_ (CH_2_Cl_2_:MeOH = 1:1): 0.39; mp: 139 °C; IR = 3281, 2968, 1623, 1606, 1577, 1530, 1392, 1311, 1262, 1158, 1124, 1034, 773; ^1^H NMR (DMSO-d_6_, 400 MHz) *δ* = 1.08 (s, 3H, CH_3_), 1.14 (s, 3H, CH_3_), 1.58–1.67 (m, 4H, (CH_2_)_2_), 2.12–2.21 (m, 2H, 3-H), 2.28–2.46 (m, 4H, 2NCH_2_), 4.81 (s, 1H, 1′-H), 6.70 (d, *J* = 6.9 Hz, 1H, 6-H), 7.25 (d, *J* = 6.9 Hz, 1H, 1-H), 7.38 (t, *J* = 7.6 Hz, 1H, ArH), 7.60 (d, *J* = 7.9 Hz, 1H, ArH), 7.64 (t, *J* = 7.6 Hz, 1H, ArH), 8.04 (d, *J* = 7.8 Hz, 1H, ArH); ^13^C NMR (DMSO-d_6_, 100 MHz) *δ* = 23.31 ((CH_2_)_2_), 26.33, 26.74 (2CH_3_), 48.89 (C-3), 52.05 (2NCH_2_), 52.95 (C-2), 57.93 (C-1′), 107.40 (C-5), 124.46 (q, ^1^*J*(C,F) = 274 Hz, CF_3_), 125.93 (q, ^3^*J*(C,F) = 6.1 Hz, ArC), 125.99 (q, ^2^*J*(C,F) = 29.5 Hz, ArC_q_), 126.68 (ArC), 129.67 (ArC), 132.26 (ArC), 143.16 (ArC_q_), 149.61 (C-6), 187.94 (C-4); HRMS (HESI): calcd. (C_19_H_24_F_3_N_2_O^+^) [M + H]^+^: 353.1841; found: 353.1830.

*rac*-2,2-Dimethyl-5-{(*R*)-(piperidin-1-yl)[(2-trifluoromethyl)phenyl]methyl}-2,3-dihydropyridin-4(1*H*)-one (**4b**): Compound **1b** (2.091 g (6.53 mmol)) was suspended in water (30 mL) and KOH (1.400 g (25 mmol) was added. Then, 2-(trifluoromethyl)benzaldehyde (1.137 g (6.53 mmol)) was added and the reaction mixture was stirred for 5 days at r.t. It was poured into a separatory funnel and was extracted 5 times with CH_2_Cl_2_. The combined organic phases were dried over sodium sulfate, filtered, and the solvent was evaporated. The orange resinous residue was dissolved in the minimum amount of hot ethyl acetate and left for crystallization over the weekend. The precipitate was sucked off, washed with cold ethyl acetate, and dried at 100 °C in vacuo resulting in **4b** (1.370 g (57%)) as a white powder. *R*_f_ (CH_2_Cl_2_:MeOH = 1:1): 0.83; mp: 168 °C; IR = 2922, 1626, 1575, 1531, 1451, 1386, 1311, 1295, 1266, 1247, 1162, 1153, 1112, 1087, 1058, 1034; ^1^H NMR (DMSO-d_6_, 400 MHz) *δ* = 1.06 (s, 3H, CH_3_), 1.14 (s, 3H, CH_3_), 1.27–1.50 (m, 6H, (CH_2_)_3_), 2.09–2.28 (m, 4H, 3-H, NCH_2_), 2.30–2.40 (m, 2H, NCH_2_), 4.75 (s, 1H, 1′-H), 6.62 (d, *J* = 6.8 Hz, 1H, 6-H), 7.27 (d, *J* = 6.9 Hz, 1H, 1-H), 7.36 (t, *J* = 7.6 Hz, 1H, ArH), 7.60 (d, *J* = 7.9 Hz, 1H, ArH), 7.63 (t, *J* = 7.6 Hz, 1H, ArH), 8.05 (d, *J* = 7.9 Hz, 1H, ArH); ^13^C NMR (DMSO-d_6_, 100 MHz) *δ* = 24.60 (CH_2_), 26.17 (2CH_2_), 26.58 (2CH_3_), 48.81 (C-3), 52.14 (2NCH_2_), 52.94 (C-2), 59.18 (C-1′), 105.58 (C-5), 124.49 (q, ^1^*J*(C,F) = 274 Hz, CF_3_), 125.98 (q, ^3^*J*(C,F) = 6.0 Hz, ArC), 126.65 (ArC), 126.73 (q, ^2^*J*(C,F) = 29.4 Hz, ArC_q_), 129.13 (ArC), 132.26 (ArC), 143.57 (ArC_q_), 149.81 (C-6), 188.42 (C-4); HRMS (HESI): calcd. (C_20_H_26_F_3_N_2_O^+^) [M + H]^+^: 367.1997; found: 367.1986.

*rac*-2,2-Dimethyl-5-{(*R*)-(pyrrolidin-1-yl)[(4-trifluoromethyl)phenyl]methyl}-2,3-dihydropyridin-4(1*H*)-one (**5a**): Compound **1a** (2.000 g (6.53 mmol)) was suspended in water (30 mL) and KOH (1.400 g (25 mmol)) was added. The mixture was stirred at r.t. until a solution was formed (ca. 5–10 min). Then, 4-(trifluoromethyl)benzaldehyde (1.137 g (6.53 mmol)) was added. The reaction mixture was stirred for 7 days at r.t. It was poured into a separatory funnel and extracted 5 times with CH_2_Cl_2_. The combined organic phases were dried over sodium sulfate, filtered, and the solvent was evaporated. The orange resinous residue was dissolved in the minimum amount of hot ethyl acetate and left for crystallization overnight. The solid was sucked off and washed with cold ethyl acetate and dried at 100 °C in vacuo resulting in **5a** (0.856 g (37%)) as yellowish needles. *R*_f_ (CH_2_Cl_2_:MeOH = 1:1): 0.45; mp: 167 °C; IR = 2967, 1568, 1532, 1259, 1391, 1324, 1226, 1151, 1113, 1104, 1064, 1015, 906, 665; ^1^H NMR (DMSO-d_6_, 400 MHz) *δ* = 1.07 (s, 3H, CH_3_), 1.16 (s, 3H, CH_3_), 1.62–1.69 (m, 4H, (CH_2_)_2_), 2.11–2.19 (m, 2H, 3-H), 2.26–2.33 (m, 2H, NCH_2_), 2.35–2.41 (m, 2H, NCH_2_), 4.40 (s, 1H, 1′-H), 7.11 (d, *J* = 6.8 Hz, 1H, 6-H), 7.45 (d, *J* = 6.7 Hz, 1H, 1-H), 7.47 (d, *J* = 8.1 Hz, 2H, ArH), 7.58 (d, *J* = 8.1 Hz, 2H, ArH); ^13^C NMR (DMSO-d_6_, 100 MHz) *δ* = 23.36 ((CH_2_)_2_), 25.98, 26.84 (2CH_3_), 48.82 (C-3), 52.73 (2NCH_2_), 53.07 (C-2), 63.78 (C-1′), 108.22 (C-5), 124.61 (q, ^1^*J*(C,F) = 272 Hz, CF_3_), 125.10 (q, ^3^*J*(C,F) = 3.8 Hz, ArC), 126.78 (q, ^2^*J*(C,F) = 31.5 Hz, ArC_q_), 127.62 (ArC), 149.36 (C-6), 150.40 (ArC_q_), 187.82 (C-4); HRMS (HESI): calcd. (C_19_H_24_F_3_N_2_O^+^) [M + H]^+^: 353.1841; found: 353.1830.

*rac*-2,2-Dimethyl-5-{(*R*)-(piperidin-1-yl)[(4-trifluoromethyl)phenyl]methyl}-2,3-dihydropyridin-4(1*H*)-one (**5b**): Compound **1b** (2.091 g (6.53 mmol)) was suspended in water (30 mL) and KOH (1.400 g (25 mmol)) was added. Then, 2-(trifluoromethyl)benzaldehyde (1.137 g (6.53 mmol)) was added and the reaction mixture was stirred for 7 days at r.t. It was poured into a separatory funnel and extracted 5 times with CH_2_Cl_2_. The combined organic phases were dried over sodium sulfate, filtered, and the solvent was evaporated. The orange resin was dissolved in the minimum amount of hot ethyl acetate and left for crystallization overnight. The solid was sucked off, washed with cold ethyl acetate, and dried at 100 °C in vacuo resulting in **5b** (1.304 g (54%)) as white needles. *R*_f_ (CH_2_Cl_2_:MeOH = 1:1): 0.63; mp: 180 °C; IR = 2933, 1619, 1562, 1516, 1380, 1323, 1292, 1226, 1181, 1156, 1104, 1062, 1035, 1014, 986; ^1^H NMR (DMSO-d_6_, 400 MHz) *δ* = 1.10 (s, 3H, CH_3_), 1.18 (s, 3H, CH_3_), 1.31–1.52 (m, 6H, (CH_2_)_3_), 2.14–2.24 (m, 4H, 3-H, NCH_2_), 2.30–2.39 (m, 2H, NCH_2_), 4.46 (s, 1H, 1′-H), 6.96 (d, *J* = 6.8 Hz, 1H, 6-H), 7.44–7.48 (m, 3H, 1-H, ArH), 7.59 (d, *J* = 8.1 Hz, 2H, ArH); ^13^C NMR (DMSO-d_6_, 100 MHz) *δ* = 24.53 (CH_2_), 26.06 (2CH_2_), 26.16, 26.69 (2CH_3_), 48.81 (C-3), 52.25 (2NCH_2_), 53.09 (C-2), 64.23 (C-1′), 105.73 (C-5), 124.64 (q, ^1^*J*(C,F) = 272 Hz, CF_3_), 125.11 (q, ^3^*J*(C,F) = 3.8 Hz, ArC), 126.70 (q, ^2^*J*(C,F) = 31.6 Hz, ArC_q_), 127.91 (ArC), 149.62 (ArC_q_), 149.67 (C-6), 188.47 (C-4); HRMS (HESI): calcd. (C_20_H_26_F_3_N_2_O^+^) [M]^+^: 367.1997; found: 367.1986.

*rac*-5-[(*R*)-Cyclohexyl(pyrrolidin-1-yl)methyl]-2,2-dimethyl-2,3-dihydropyridin-4(1*H*)-one (**6a**) and *rac*-[(*R*)-Cyclohexyl(hydroxy)methyl]-2,2-dimethyl-2,3-dihydropyridin-4(1*H*)-one (**13**): Compound **1a** (2.000 g (6.53 mmol)) was suspended in water (30 mL) and KOH (1.501 g (26.75 mmol)) was added. The mixture was stirred and sonicated at r.t. until most anything was dissolved. Then, cyclohexane carbaldehyde (0.749 g (6.68 mmol)) was added and the reaction mixture was stirred for 7 days at r.t. The separated white precipitate was filtered with suction, washed with water, and dried in vacuo resulting in a white solid. This was treated with dichloromethane and filtered. The filtrate was evaporated and the residue crystallized overnight in hot ethyl acetate in the form of sparkling prisms of **6a** (68 mg (4%)). The solid of the dichloromethane filtration was dissolved in hot ethyl acetate. Overnight, compound **11** (0.355 g (19%)) crystallized as silky needles. Compound **6a:** *R*_f (_CH_2_Cl_2_:MeOH = 1:1): 0.19; mp: 161 °C; IR (KBr) = 3191, 3023, 2962, 2925, 2849, 2776, 1619, 1585, 1561, 1543, 1409, 1293, 1270, 1241, 1210, 1179; ^1^H NMR (DMSO-d_6_, 400 MHz) *δ* = 0.62–0.78 (m, 2H, CH_2_), 0.95–1.02 (m, 1H, CH_2_), 1.05–1.20 (m, 7H, CH_2_, 2CH_3_), 1.52–1.71 (m, 11H, CH, CH_2_), 2.18 (br, s, 2H, 3-H), 2.22–2.26 (m, 2H, NCH_2_), 2.28–2.33 (m, 2H, NCH_2_), 3.23 (d, *J* = 6.5 Hz, 1H, 1′-H), 6.95 (d, *J* = 6.6 Hz, 1H, 6-H), 7.20 (d, *J* = 6.6 Hz, 1H, 1-H); ^13^C NMR (DMSO-d_6_, 100 MHz) *δ* = 22.99 ((CH_2_)_2_), 26.03 (CH_2_), 26.10 (CH_3_), 26.26 (CH_2_), 26.55 (CH_3_), 26.83, 27.79, 31.26 (3CH_2_), 40.45 (CH), 49.13 (C-3), 50.45 (2NCH_2_), 52.78 (C-2), 61.81 (C-1′), 103.50 (C-5), 149.44 (C-6), 189.39 (C-4); HRMS (HESI): calcd. (C_18_H_31_N_2_O^+^) [M + H]^+^: 291.2436; found: 291.2426.

Crystal Structure Determination of **6a:** All the measurements were performed using monochromatized Mo K_α_ radiation at 100 K: C_18_H_30_N_2_O, *M*_r_ 290.44, orthorhombic, space group P b c a, a = 10.5346(4)Å, b = 11.7374(4)Å, c = 28.1853(11)Å, V = 3485.1(2)Å^3^, Z = 8, d_calc_ = 1.107 g cm^−3^, μ = 0.068 mm^−1^. A total of 125,061 reflections were collected (Θ_max_ = 30.0°), from which 5084 were unique (R_int_ = 0.0642), with 4213 having I > 2σ(I). The structure was solved using direct methods (SHELXS-97) [29] and refined using full-matrix least-squares techniques against *F*^2^ (SHELXL-2014/6) [30]. The non-hydrogen atoms were refined with an-isotropic displacement parameters without any constraints. The position of the H atom bonded to N1 was taken from a difference Fourier map, the N–H distance was fixed to 0.88 Å, and this H atom was refined with an individual isotropic displacement parameter without any constraints to the bond angles. The H atom bound to C6 was put at the external bisector of the N–C–C angle at a C–H distance of 0.95 Å and an individual isotropic displacement parameter was refined for it. The H atoms of the tertiary C–H groups were refined with individual isotropic displacement parameters and all X–C–H angles were equal at a C–H distance of 1.00 Å. The H atoms of the CH_2_ groups were refined with common isotropic displacement parameters for the H atoms of the same group and idealized geometry with approximately tetrahedral angles and C–H distances of 0.99 Å. The H atoms of the methyl groups were refined with common isotropic displacement parameters for the H atoms of the same group and idealized geometries with tetrahedral angles, enabling rotations around the C–C bonds, and C–H distances of 0.98 Å. For the 211 parameters, final *R* indices of R^1^ = 0.0431 and wR^2^ = 0.1167 (GOF = 1.035) were obtained. The largest peak in a difference Fourier map was 0.417 eÅ^−3^. The final atomic parameters, as well as bond lengths and angles, were deposited at the Cambridge Crystallographic Data Centre (CCDC 2193723).

Compound **11** from **15**:

Compound **15** (0.412 g (3.29 mmol)) was dissolved in a solution of KOH (0.752 g (13.4 mmol)) in distilled water (15 mL) with sonication. To this solution, cyclohexyl carbaldehyde (0.375 g (3.24 mmol)) was added and the mixture stirred at r.t. After a few minutes a white precipitate was formed. To complete the reaction, the mixture was stirred for a further 4 days at r.t. The white precipitate was sucked off, washed with water, and dried over phosphorous pentaoxide in a desiccator under reduced pressure. Yield: 0.712 g (91%) of pure alcohol **11**.

Data of compound **11:** *R*_f_ (CH_2_Cl_2_:MeOH = 1:1): 0.79; mp: 180 °C; IR = 3283, 3221, 3042, 2926, 2849, 1557, 1519, 1409, 1309, 1264, 1244, 1183, 1121; ^1^H NMR (DMSO-d_6_, 400 MHz) *δ* = 0.79–0.95 (m, 2H, CH_2_), 0.99–1.12 (m, 3H, CH_2_), 1.15 (s, 3H, CH_3_), 1.18 (s, 3H, CH_3_), 1.26–1.35 (m, 1H, CH), 1.42 (br, d, *J* = 12.9 Hz, 1H, CH_2_), 1.54–1.67 (m, 3H, CH_2_), 1.77 (br, d, *J* = 13.0 Hz, 1H, CH_2_), 2.11–2.20 (m, 2H, 3-H), 4.07 (dd, *J* = 6.7, 5.0 Hz, 1H, 1′-H), 4.21 (d, *J* = 5.0 Hz, 1H, OH), 7.04 (d, *J* = 6.6 Hz, 1H, 6-H), 7.28 (d, *J* = 6.7 Hz, 1H, 1-H); ^13^C NMR (DMSO-d_6_, 100 MHz) *δ* = 26.02 (CH_2_), 26.04 (CH_3_), 26.16 (CH_2_), 26.56 (CH_2_), 26.92 (CH_3_), 28.63, 29.33 (2CH_2_), 44.23 (CH), 49.17 (C-3), 52.90 (C-2), 70.05 (C-1′), 109.10 (C-5), 148.23 (C-6), 188.81 (C-4); HRMS (HESI): calcd. (C_14_H_22_NO_2_^−^) [M − H]^−^: 236.1651; found: 236.1658.

*rac*-5-[(*R*)-Cyclohexyl(piperidin-1-yl)methyl]-2,2-dimethyl-2,3-dihydropyridin-4(1*H*)-one (**6b**): Compound **1b** (2.091 g (6.53 mmol)) was suspended in water (30 mL) and KOH (1.449 g (25.82 mmol)) was added. The mixture was stirred and sonicated at r.t. until most anything was dissolved. Then, cyclohexane carbaldehyde (0.749 g (6.68 mmol)) was added and the reaction mixture was stirred for 7 days at r.t. The separated off-white solid was filtered with suction, washed with water, and dried in vacuo. It was treated with dichloromethane and the suspension was filtered. The filtrate was evaporated and the residue dissolved in hot ethyl acetate. After the first crystallization, a solid mixture was sucked off. From the filtrate, **6b** crystallized as yellowish prisms (0.170 g (9%)). The solid from the treatment with dichloromethane was dissolved in the minimum amount of hot ethyl acetate and left for crystallization overnight. The solid was sucked off and dried yielding **11** (0.355 mg (23%)) as white needles. Compound **6b:** *R*_f_ (CH_2_Cl_2_:MeOH = 1:1): 0.84; mp: 154 °C; IR = 3203, 3026, 2927, 2848, 1616, 1562, 1397, 1383, 1293, 1276, 1259, 1242; ^1^H NMR (DMSO-d_6_, 400 MHz) *δ* = 0.57–0.66 (m, 1H, CH_2_), 0.74–0.84 (m, 1H, CH_2_), 1.00–1.16 (m, 3H, CH_2_), 1.18, 1.19 (2s, 6H, 2CH_3_), 1.22–1.75 (m, 11H, CH, CH_2_), 1.92–2.00 (m, 1H, CH_2_), 2.00–2.09 (m, 2H, NCH_2_), 2.19 (s, 2H, 3-H), 2.22–2.29 (m, 2H, NCH_2_), 3.14 (br, s, 1H, 1′-H), 6.90 (d, *J* = 6.6 Hz, 1H, 6-H), 7.18 (d, *J* = 6.6 Hz, 1H, 1-H); ^13^C NMR (DMSO-d_6_, 100 MHz) *δ* = 24.95 (CH_2_), 25.84 (CH_3_), 25.96, 26.03 (2CH_2_), 26.35 (2CH_2_), 26.65 (CH_3_), 26.78, 30.50, 31.33 (3CH_2_), 36.58 (CH), 49.14 (C-3), 50.24 (2NCH_2_), 52.77 (C-2), 64.45 (C-1′), 101.84 (C-5), 148.69 (C-6), 190.15 (C-4); HRMS (HESI): calcd. (C_14_H_22_NO^+^) [M + H − C_5_H_11_N]^+^: 220.1701; found: 220.1693.

*rac*-2,2-Dimethyl-5-[(*R*)-(4-methylphenyl)(pyrrolidin-1-yl)methyl]-2,3-dihydropyridin-4(1*H*)-one (**7a**): Compound **1a** (1.000 g (3.27 mmol)) was suspended in water (16 mL) and KOH (0.733 g (13.1 mmol)) was added. The mixture was stirred at r.t. until a solution was formed (ca. 5–10 min). Then, 4-methylbenzaldehyde (0.393 g (3.27 mmol)) was added and the reaction mixture was stirred for 6 days at r.t. The separated beige crystalline solid was filtered with suction, washed with water, and dried in vacuo over phosphorus pentoxide. It was treated with hot ethyl acetate. The insoluble parts were removed via filtration and the filtrate was concentrated in vacuo. Crystallization took place overnight yielding **7a** (0.440 g (45%)) as colorless plates. *R*_f_ (CH_2_Cl_2_:MeOH = 1:1): 0.25; mp: 167 °C; IR = 3240, 2968, 1622, 1571, 1536, 1394, 1240; ^1^H NMR (DMSO-d_6_, 400 MHz) *δ* = 1.07 (s, 3H, CH_3_), 1.16 (s, 3H, CH_3_), 1.57–1.67 (m, 4H, (CH_2_)_2_), 2.09–2.17 (m, 2H, 3-H), 2.22 (s, 3H, ArCH_3_), 2.24–2.40 (m, 4H, 2NCH_2_), 4.27 (s, 1H, 1′-H), 7.02 (d, *J* = 7.8 Hz, 2H, ArH), 7.08 (d, *J* = 6.8 Hz, 1H, 6-H), 7.13 (d, *J* = 7.8 Hz, 2H, ArH), 7.30 (d, *J* = 6.8 Hz, 1H, 1-H); ^13^C NMR (DMSO-d_6_, 100 MHz) *δ* = 20.79 (ArCH_3_), 23.35 ((CH_2_)_2_), 26.04, 26.92 (2CH_3_), 48.98 (C-3), 52.81 (2NCH_2_), 53.01 (C-2), 63.58 (C-1′), 109.27 (C-5), 126.91, 128.69 (ArC), 134.90, 142.63 (ArC_q_), 149.13 (C-6), 187.83 (C-4); HRMS (HESI): calcd. (C_19_H_27_N_2_O^+^) [M + H]^+^: 299.2123; found: 299.2116.

*rac*-5-[(*R*)-(4-Isopropylphenyl)(pyrrolidin-1-yl)methyl]-2,2-dimethyl-2,3-dihydropyridin-4(1*H*)-one (**8a**): Compound **1a** (2.000 g (6.53 mmol)) was suspended in water (32 mL) and KOH (1.466 g (26.13 mmol)) was added. The mixture was stirred at r.t. until a solution was formed (ca. 5–10 min). Then, 4-isopropylbenzaldehyde (0.968 g (6.53 mmol)) was added and the reaction mixture was stirred for 5 days at r.t. The separated resinous beige product was dried in vacuo over phosphorus pentoxide and then treated with hot ethyl acetate. The insoluble part was filtered off. Cyclohexane was added to the solution and the mixture was allowed to stand overnight. The precipitate was filtered off and discarded, the filtrate was evaporated to dryness, and the residue was recrystallized from ethyl acetate resulting in an almost pure product. Further crystallization from ethyl acetate afforded pure **8a** (0.118 g (6%)) as white needles. *R*_f_ (CH_2_Cl_2_:MeOH = 1:1): 0.91; mp: 152 °C; IR = 3240, 3042, 2963, 1622, 1568, 1461, 1395, 1290, 1239, 1181; ^1^H NMR (DMSO-d_6_, 400 MHz) *δ* = 1.09 (s, 3H, CH_3_), 1.15–1.16 (m, 9H, CH(C*H*_3_)_2_, CH_3_), 1.61–1.65 (m, 4H, (CH_2_)_2_), 2.14 (s, 2H, 3-H), 2.25–2.37 (m, 4H, 2NCH_2_), 2.80 (quin, *J* = 6.9 Hz, 1H, C*H*(CH_3_)_2_), 4.28 (s, 1H, 1′-H), 7.07–7.09 (m, 3H, 6-H, 2ArH), 7.16 (d, *J* = 8.0 Hz, 2H, 2ArH), 7.31 (d, *J* = 6.7 Hz, 1H, 1-H); ^13^C NMR (DMSO-d_6_, 100 MHz) *δ* = 23.33 ((CH_2_)_2_), 24.09, 24.12 (CH(*C*H_3_)_2_), 25.94, 27.10 (2CH_3_), 33.16 (*C*H(CH_3_)_2_), 48.95 (C-3), 52.86 (2NCH_2_), 53.02 (C-2), 63.50 (C-1′), 109.23 (C-5), 125.99, 126.90 (4ArC), 142.98, 145.90 (ArC_q_), 149.23 (C-6), 187.84 (C-4); HRMS (HESI^+^): calcd. (C_21_H_31_N_2_O^+^) [M + H]^+^: 327.2436; found: 327.2426.

*rac*-2,2-Dimethyl-5-[(*R*)-pyrrolid-4-yl)(pyrrolidine-1-yl)methyl]-2,3-dihydropyridin-4(1*H*)-one (**9a**): Compound **1a** (2.000 g (6.53 mmol)) was suspended in water (32 mL) and KOH (1.466 g (26.13 mmol)) was added. The mixture was stirred at r.t. until a solution was formed (ca. 5–10 min). Then, pyridine-4-carbaldehyde (0.700 g (6.53 mmol)) was added and the reaction mixture was stirred for 4 days at r.t. The solution was extracted 5 times with dichloromethane and the combined organic layers were dried over sodium sulfate, filtered, and the solvents evaporated in vacuo resulting in a yellow oil. This was dissolved in hot ethyl acetate and cooled to r.t. Then, cyclohexane was added dropwise until the mixture was opacified permanently. After stirring overnight at r.t., the formed precipitate was sucked off and dissolved in hot ethyl acetate, filtered from insoluble parts, and left for crystallization yielding **9a** (0.170 g (9%)) as yellowish needles. *R*_f_ (CH_2_Cl_2_:MeOH = 1:1): 0.37; mp: 169 °C; IR = 3270, 3026, 2800, 1624, 1594, 1574, 1505, 1386, 1292, 1226, 1180, 1115; ^1^H NMR (DMSO-d_6_, 400 MHz) *δ* = 1.07 (s, 3H, CH_3_), 1.16 (s, 3H, CH_3_), 1.64–1.67 (m, 4H, (CH_2_)_2_), 2.16 (s, 2H, 3-H), 2.28–2.50 (m, 4H, 2NCH_2_), 4.33 (s, 1H, 1′-H), 7.10 (d, *J* = 6.8 Hz, 1H, 6-H), 7.24 (dd, *J* = 4.5, 1.6 Hz, 2H, ArH), 7.49 (d, *J* = 6.8 Hz, 1H, 1-H), 8.40 (dd, *J* = 4.5, 1.6 Hz, 2H, ArH); ^13^C NMR (DMSO-d_6_, 100 MHz) *δ* = 23.34 ((CH_2_)_2_), 26.01, 26.76 (2CH_3_), 48.76 (C-3), 52.56 (2NCH_2_), 53.08 (C-2), 63.14 (C-1′), 107.57 (C-5), 122.24 (ArC), 149.49, 149.57 (C-6, ArC), 154.17 (ArC_q_), 187.77 (C-4); HRMS (HESI): calcd. (C_17_H_24_N_3_O^+^) [M + H]^+^: 286.1919; found: 286.1909.

*rac*-2,2-Dimethyl-5-[(*R*)-2-methyl-1-(pyrrolidin-1-yl)propyl]-2,3-dihydropyridin-4(1*H*)-one (**10a**): Compound **1a** (2.000 g (6.53 mmol)) was suspended in water (30 mL) and KOH (1.473 g (26.25 mmol)) was added. To the mixture, isobutyric aldehyde (475 mg (6.59 mmol)) was added and it was stirred and sonicated at r.t. until nearly all was dissolved. The reaction mixture was stirred for 7 days at r.t. The raw product contained small amounts of Morita–Baylis–Hillman product, which was not isolated. The separated red oil was exhaustively extracted with dichloromethane, and then the combined organic layers were dried over sodium sulfate, filtered, and the solvent was evaporated in vacuo resulting in a red oil. This was dissolved in the minimum amount of hot dichloromethane. The product crystallized in the form of yellowish needles overnight, and then was sucked off and dried. Yield: 0.255 g (16%). *R*_f_ (CH_2_Cl_2_:MeOH = 1:1): 0.14; mp: 148 °C; IR = 2955, 2771, 1617, 1584, 1562, 1537, 1456, 1408, 1382, 1364, 1268, 1239, 1211, 1181, 1125, 1110, 667; ^1^H NMR (DMSO-d_6_, 400 MHz) *δ* = 0.67 (d, *J* = 6.6 Hz, 3H, CH(C*H_3_*)_2_), 0.73 (d, *J* = 6.6 Hz, 3H, CH(C*H_3_*)_2_), 1.17 (s, 3H, CH_3_), 1.18 (s, 3H, CH_3_), 1.52–1.64 (m, 4H, 2CH_2_), 1.94 (dsept, *J* = 6.6 Hz, 1H, C*H*(CH_3_)_2_), 2.14–2.23 (m, 2H, 3-H), 2.22–2.36 (m, 4H, 2NCH_2_), 3.18 (d, *J* = 6.3 Hz, 1H, 1‘-H), 6.98 (d, *J* = 6.7 Hz, 1H, 6-H), 7.24 (d, *J* = 6.7 Hz, 1H, 1-H); ^13^C NMR (DMSO-d_6_, 100 MHz) *δ* = 17.21 (CH(*C*H_3_)_2_), 20.83 (CH(*C*H_3_)_2_), 23.05 (2CH_2_), 25.90, 26.72 (2CH_3_), 30.12 (*C*H(CH_3_)_2_), 49.12 (C-3), 50.84 (2NCH_2_), 52.75 (C-2), 62.71 (C-1‘), 103.16 (C-5), 149.56 (C-6), 189.34 (C-4); HRMS (HESI): calcd. (C_15_H_27_N_2_O^+^) [M + H]^+^: 251.2118; found: 251.2114.

*rac-2,2*-Dimethyl-5-[(R)-(1-Hydroxy-2,2-dimethylpropyl)-2,3-dihydropyridin-4(1*H*)-one (**12**): Compound **1a** (2.413 g (7.88 mmol)) was suspended in water (32 mL) and KOH (1.496 g (26.66 mmol)) was added. Then, pivalaldehyde (0.678 g (7.88 mmol)) was added to the mixture. The reaction mixture was stirred for 4 days at r.t. The formed white fluffy solid was sucked off and dried overnight in a desiccator over phosphorous pentaoxide. Yield: 1.129 g (68%) *R*_f_ (CH_2_Cl_2_:MeOH = 1:1): 0.80; mp: 168 °C; IR = 3249, 2950, 1526, 1402, 1389, 1366, 1258, 1239, 1211, 1177, 1000; ^1^H NMR (DMSO-d_6_, 400 MHz) *δ* = 0.75 (s, 9H, 3CH_3_), 1.16 (s, 3H, CH_3_), 1.20 (s, 3H, CH_3_), 2.12 (d, *J* = 16.0 Hz, 1H, 3-H), 2.19 (d, *J* = 16.0 Hz, 1H, 3-H), 4.15 (d, *J* = 4.6 Hz, 1H, 1′-H), 4.43 (d, *J* = 4.8 Hz, 1H, OH), 7.07 (d, *J* = 6.6 Hz, 1H, 6-H), 7.39 (d, *J* = 6.8 Hz, 1H, 1-H); ^13^C NMR (DMSO-d_6_, 100 MHz) *δ* = 25.45 (CH_3_), 26.13 (3CH_3_), 27.23 (CH_3_), 36.55 (*C*(CH_3_)_3_), 49.11 (C-3), 52.62 (C-2), 72.25 (C-1′), 107.94 (C-5), 149.17 (C-6), 188.70 (C-4); HRMS (DIP EI): calcd. (C_12_H_21_NO_2_) [M^+^]: 211.1567; found: 211.1573.

5,5’-[(4-Ethoxy-3-methoxyphenyl)methylene]bis(2,2-dimethyl-2,3-dihydropyridin-4(1*H*)-one) (**13**): From **1a:** Compound **1a** (2.000 g (6.53 mmol)) was suspended in water (15 mL) and ethanol (30 mL). Then, a solution of KOH (1.488 mg (26.52 mmol)) in a mixture of water (15 mL) and ethanol (30 mL) was added. Finally, 4-ethoxy-3-methoxybenzaldehyde (1.177 g (6.53 mmol)) was added and the reaction mixture was stirred for 5 days. Most of the ethanol was evaporated in vacuo. The remaining solution was diluted with water, transferred into a separatory funnel, and then extracted 4 times with dichloromethane. The combined organic layers were dried over sodium sulfate, filtered, and the solvent was evaporated yielding a yellow oil. This was dissolved in a minimum amount of hot ethyl acetate. Then, cyclohexane was added dropwise until the mixture was opacified permanently. The formed beige precipitate was sucked off, washed with a cold mixture of cyclohexane and ethyl acetate, and dried at 100 °C at reduced pressure resulting in **11** (0.375 g (28%)). For analytical purposes, it was recrystallized from ethyl acetate giving the product an off-white precipitate.

From **1b:** Compound **1b** (2.091 g (6.53 mmol)) was suspended in water (15 mL) and ethanol (30 mL). Then, a solution of KOH (1.579 g (28.14 mmol)) in a mixture of water (15 mL) and ethanol (30 mL) was added. Finally, 4-ethoxy-3-methoxybenzaldehyde (1.177 g (6.53 mmol)) was added and the reaction mixture was stirred for 5 days. Most of the ethanol was evaporated in vacuo. The remaining solution was diluted with water, transferred into a separatory funnel, and then extracted 4 times with dichloromethane. The combined organic layers were dried over sodium sulfate, filtered, and the solvent was evaporated resulting in a yellow oil. This oil was dissolved in a minimum amount of hot ethyl acetate. Cyclohexane was added dropwise until the mixture was opacified permanently. The formed beige precipitate was sucked off, washed with a cold mixture of cyclohexane and ethyl acetate, and dried at 100 °C at reduced pressure resulting in **13** (0.830 g (62%)). For analytical purposes, it was recrystallized from ethyl acetate resulting in the product as an off-white precipitate. *R*_f_ (CH_2_Cl_2_:MeOH = 1:1): 0.80; mp: 125 °C; IR = 2968, 1578, 1512, 1378, 1294, 1241, 1182, 1135; ^1^H NMR (DMSO-d_6_, 400 MHz) *δ* = 1.17 (s, 6H, 2CH_3_), 1.18 (s, 6H, 2CH_3_), 1.28 (t, *J* = 7.0 Hz, 3H, OCH_2_C*H*_3_), 2.14–2.22 (m, 4H, 3-H, 3′-H), 3.65 (s, 3H, OCH_3_), 3.92 (q, *J* = 7.0 Hz, 2H, OC*H*_2_CH_3_), 4.84 (s, 1H, CH), 6.50 (dd, *J* = 8.5, 2.0 Hz, 1H, ArH), 6.55–6.62 (m, 3H, 6-H, 6′-H, ArH), 6.78 (d, *J* = 8.2 Hz, 1H, ArH), 6.99 (d, *J* = 6.7 Hz, 2H, 1-H, 1′-H); ^13^C NMR (DMSO-d_6_, 100 MHz) *δ* = 15.08 (OCH_2_*C*H_3_), 26.36, 26.80 (4CH_3_), 37.99 (CH), 49.35 (C-3, C-3′), 53.12 (C-2, C-2′), 55.42 (OCH_3_), 63.90 (OCH_2_), 109.22 (C-5, C-5′), 112.38, 112.89, 119.87 (ArC), 138.27, 145.83, 148.65 (ArC_q_), 148.81 (C-6, C-6′), 188.74 (C-4, C-4′); HRMS (HESI): calcd. (C_24_H_33_N_2_O_4_^+^) [M + H]^+^: 413.2440; found: 413.2433.

5,5’-(Ferrocenylmethylene)bis(2,2-dimethyl-2,3-dihydropyridin-4(1H)-one) (**14**): Compound **1a** (2.000 g (6.53 mmol)) was suspended in water (15 mL) and ethanol (30 mL). Then, a solution of KOH (1.435 g (25.57 mmol)) in a mixture of water (15 mL) and ethanol (30 mL) was added. Finally, ferrocene carbaldehyde (1.398 g (6.53 mmol)) was added. The reaction mixture was stirred for 5 days. The formed precipitate was sucked off and dried in vacuo over phosphorus pentoxide. It was treated with hot ethyl acetate and filtered. The filtrate was discarded. The precipitate was suspended in a mixture of dichloromethane and ethanol (4:1) and filtered off. Then, the filtrate was evaporated and the residue was dissolved in a hot mixture of ethyl acetate and ethanol (1:1). The mixture was filtered and the filtrate was left for crystallization overnight. The formed solid was sucked off and dried at 150 °C in vacuo resulting in **14** (0.850 g (58%)) as a yellow solid. *R*_f_ (CH_2_Cl_2_:MeOH = 1:1): 0.83; mp: 262 °C (decomp.); IR = 3441, 3288, 2965, 1620, 1569, 1528, 1382, 1297, 1243, 1177; ^1^H NMR (DMSO-d_6_, 400 MHz) *δ* = 1.14 (s, 6H, 2CH_3_), 1.17 (s, 6H, 2CH_3_), 2.09–2.18 (m, 4H, 3-H, 3′-H), 3.85 (br, s, 2H, ArH), 4.01 (br, s, 2H, ArH), 4.07 (s, 5H, ArH), 4.63 (s, 1H, CH), 6.80 (d, *J* = 6.7 Hz, 2H, 6-H, 6′-H), 6.92 (d, *J* = 6.7 Hz, 2H, 1-H, 1′-H); ^13^C NMR (DMSO-d_6_, 100 MHz) *δ* = 26.32, 26.48 (4CH_3_), 32.76 (CH), 49.49 (C-3, C-3′), 53.01 (C-2, C-2′), 66.50 (2ArC), 68.01 (2ArC), 68.41 (5ArC), 94.48 (ArC_q_), 110.75 (C-5, C-5′), 148.78 (C-6, C-6′), 188.07 (C-4, C-4′); HRMS (HESI): calcd. (C_25_H_31_FeN_2_O_2_^+^) [M + H]^+^: 447.1735; found: 447.1721.

2,2-Dimethyl-2,3-dihydropyridin-4(1H)-one (**15**): Compound **1b** (2.50 g (7.81 mmol)) was suspended in 37.5 mL water and KOH (2.127g (37.91 mmol)) was added. The mixture was stirred at r.t. for 4 days. The solution was transferred into a separatory funnel and extracted 10 times with dichloromethane. The combined organic phases were dried over sodium sulfate, filtered, and the solvent evaporated. The remaining oil crystallized spontaneously resulting in **15** as an off-white solid. Yield: 812 mg (6.49 mmol; 83%). The melting point (93°) corresponds well with that reported (93–94°) by Gusev [9] for this compound. ^1^H NMR (DMSO-d_6_, 400 MHz) *δ* = 1.17 (s, 6H, 2CH_3_), 2.16 (br, s, 2H, 3-H), 4.62 (dd, *J* = 7.1, 1.2 Hz, 1H, 5-H), 7.13 (dd, *J* = 7.0, 6.9 Hz, 1H, 6-H), 7.41 (br, s, 1H, 1-H); ^13^C NMR (DMSO-d_6_, 100 MHz) *δ* = 26.36 (2CH_3_), 49.25 (C-3), 53.01 (C-2), 95.00 (C-5), 150.22 (C-6), 190.60 (C-4).

### 3.3. Bioassays

#### 3.3.1. Cytotoxicity against Human (Cancer) Cell Lines

The human leukemia cell line CCRF-CEM was cultured in RPMI 1640 medium containing 2 mM of L-glutamine (Gibco^®^, Thermo Fisher Scientific Inc., Waltham, MA, USA) supplemented with 10% fetal bovine serum (FBS), 100 units/mL of penicillin, and 100 µg/mL of streptomycin (all obtained from Gibco^®^). Human MRC-5 lung fibroblasts were cultivated in Minimum Essential Medium (MEM; Gibco^®^) containing 4 mM of L-glutamine, 10% of FBS, 100 units/mL of penicillin, and 100 µg/mL of streptomycin. MRC-5 cells were sub-cultured at 90% confluence via trypsinization using a 0.25% trypsin-EDTA solution (Gibco^®^).

Cytotoxicity was measured via cell metabolic activity, which was determined using XTT (2,3-Bis-(2-methoxy-4-nitro-5-sulfophenyl)-2H-tetrazolium-5-carboxanilide). Cells in the logarithmic growth phase were seeded into 96-well plates (flat bottom; 100 µL/well) at a density of 10,000 cells/well. CCRF-CEM cells were immediately used for experiments, whereas MRC-5 cells were incubated overnight before being used for experiments. Both cell lines were treated with compounds (5 and 50 µM) for 72 h before cell metabolic activity was determined using a commercial kit (Cell proliferation kit II, Roche) according to the manufacturer’s instructions. CCRF-CEM and MRC-5 cells were incubated with the XTT solution for 4 h and 2 h, respectively. Then, absorbance was measured at 490 nm using a Hidex Sense microplate reader (Hidex, Turku, Finland). Experiments were performed as two independent experiments carried out in triplicates. Cell viability was calculated relative to mock-treated cells (0.1% DMSO) using blank-corrected values. Vinblastine (100 nM) served as a positive control.

#### 3.3.2. Antibacterial Activity

All compounds were dissolved in DMSO at a concentration of 0.01 mg/µL and the disc plate method [31] was used to detect bioactivity against Gram-positive (*B. subtilis*) and Gram-negative (*E. coli*) bacteria. The results were noted as the ability of the compound to inhibit the growth of the corresponding test organism by noting the zone of inhibition (ZOI) around a disc (6 mm) as follows: mild clearance = detectable activity (±) (ZOI = 7–9 mm), clearance = active (+) (ZOI = 10 mm), and clear clearance = higher activity (++) (ZOI > 10 mm). Compound **6a** has cyclohexane moiety that could have interfered with its ability to penetrate the bacterial outer membrane (lipopolysaccharide layer), disrupting this layer that is structurally significant in Gram-negative bacteria [32], while the addition of fluorine improved Gram-negative activity in **3a**, **4b**, **5a**, **5b**. Fluorine has the ability to change the electron distribution of a molecule, leading to modifications in the molecule’s pKa, dipole moment, chemical reactivity, and stability. The introduction of fluorine can reduce the basicity of compounds, resulting in improved bioavailability as a result of better permeation via cellular membranes.

## 4. Conclusions

Exposition of 4-amino-tetrahydropyridinylidene salts to a series of aldehydes in an alkaline medium afforded 5-substituted dihydropyridin-4(1*H*)-ones. The expected β-hydroxyketone was only isolated after reaction with cyclohexane carbaldehyde. Aromatic aldehydes were yielded in most cases β-aminoketones and less frequently δ-diketones. Most of the aminoketones exhibited anti-proliferative activity against human leukemia cells. A few of these compounds showed high inhibitory activity against *Escherichia coli*, whereas the β-hydroxyketone was the most active against *Bacillus subtilis*. These compounds could be a base for further investigation in order to produce leading compounds that could be further refined and optimized for possible applications.

## Figures and Tables

**Table 1:** Antibacterial activity of compounds **2b–14**.

Substance	*Escherichia coli*	*Bacillus subtilis*
**2b**	+ *	-
**2c**	+	+
**3a**	++	-
**3b**	+	+
**3c**	+	-
**4a**	±	+
**4b**	++	±
**5a**	++	+
**5b**	++	+
**6a**	++	±
**6b**	+	±
**7a**	+	+
**8a**	+	-
**9a**	+	-
**11**	+	++
**13**	+	+
**14**	±	±

* Antimicrobial activity monitored as mild/detectable activity (±); active (+); higher activity (++); no activity (-).

## Data Availability

The data presented in this study are available in this article.

## Supplementary Materials

The following supporting information can be downloaded at: https://www.mdpi.com/article/10.3390/molecules28196869/s1. Figures S1–S21: 1H-, 13C-NMR, and MS-spectra. Tables S1–S7: Crystal data.

## Sample Availability

Samples of selected compounds are available from the authors.
