# Supplementary material for: Formation of 5-Aminomethyl-2,3-dihydropyridine-4(1H)-ones from 4-Amino-tetrahydropyridinylidene Salts
Source: Molecules. 2023 Sep 29;28(19):6869. doi: 10.3390/molecules28196869 (PMC10574582; doi:10.3390/molecules28196869)
Supplement: Supplementary file 1 [file molecules-28-06869-s001.zip › molecules-2638519-supplementary.pdf]

Supplementary Information

Formation of 5-Aminomethyl-2,3-Dihydropyridine-4(1*H*)-Ones from 4-Amino-Tetrahydropyridinylidene Salts

Werner Seebacher [1,*], Michael Hoffelner [1], Ferdinand Belaj [2], Teresa Pirker [3], Muaaz Alajlani [4], Rudolf Bauer [3], Eva-Maria Pferschy-Wenzig [3], Robert Saf [5] and Robert Weis [1]

[1] Pharmaceutical Chemistry, Institute of Pharmaceutical Sciences, University of Graz, Schubertstrasse 1, 8010 Graz, Austria; michael.hoffelner@edu-graz.at (M.H.); robert.weis@uni-graz.at (R.W.)
[2] Institute of Chemistry, University of Graz, Schubertstrasse 1, 8010 Graz, Austria; ferdinand.belaj@uni-graz.at
[3] Pharmacognosy, Institute for Pharmaceutical Sciences, University of Graz, Beethovenstrasse 8, 8010 Graz, Austria;
teresa.pirker@uni-graz.at (T.P.); rudolf.bauer@uni-graz.at (R.B.); eva-maria.wenzig@uni-graz.at (E.-M.P.-W.)
[4] Faculty of Pharmacy, Al-Sham Private University, Tal, 011 Damascus, Syria; muaaz.alajlani.foph@aspu.edu.sy
[5] Institute for Chemistry and Technology of Materials (ICTM), Graz University of Technology, Stremayrgasse 9, 8010 Graz, Austria; robert.saf@tugraz.at
* Correspondence: we.seebacher@uni-graz.at; Tel.: +43-316-380-5383; Fax: +43-316-380-9846

Abstract: 4-amino-tetrahydropyridinylidene salts and especially their N-alkyl-aryl derivatives show antiprotozoal potencies. Therefore, we tried an aldol reaction using the salts and aryl aldehydes in basic milieu. Instead of the awaited aldol products, an additional migration of the amine residue from position 4 to 5 was observed which formed together with the aldehyde dihydropyridine-4(1H)-ones bearing an amino aryl- moiety in position 5. The new compounds were investigated for their antiprotozoal, anticancer and antibacterial properties.

1. NMR and MS spectra of compounds **2a—12**………………Figures **S1-S21**
2. Crystal data of **6a**………………………………………… .Tables 1-7

Figure S1. ^{1}H NMR at 400 MHz, NOE's, ^{13}C NMR at 100 MHz spectra, HMBC and MS spectra for compound **2a**

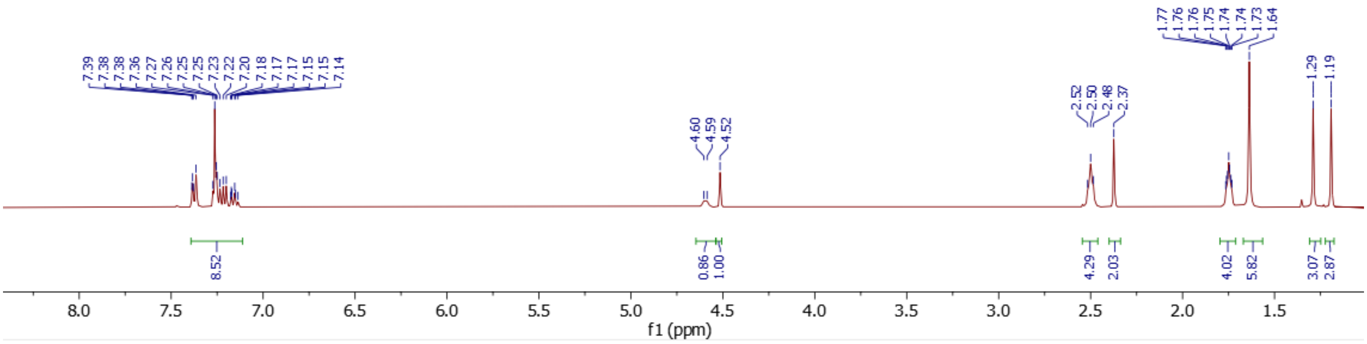

NOE from 1-H to 6-H and CH_3

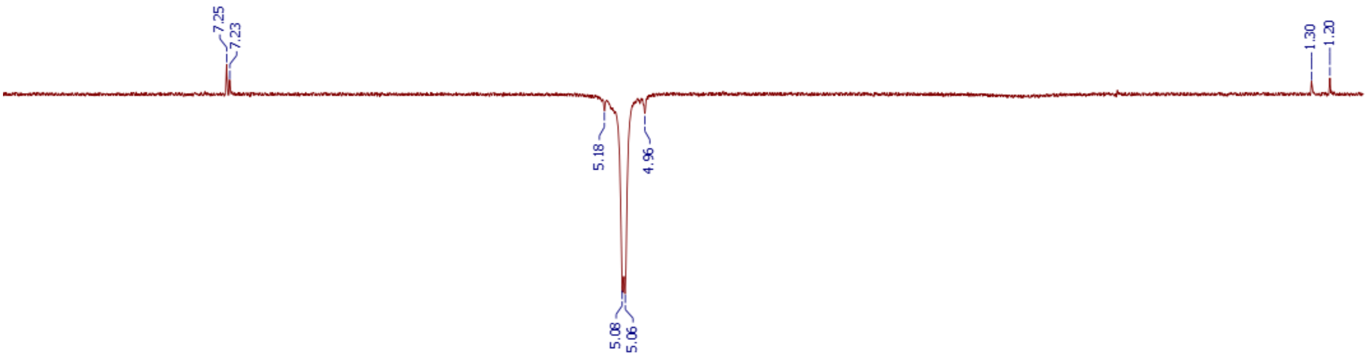

NOE from NCH_2 to 1'-H and to ArH

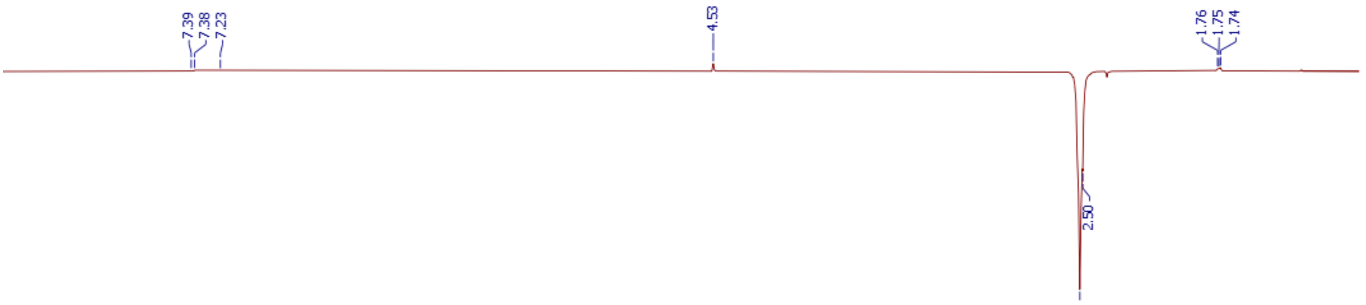

NOE from 3-H to CH_3

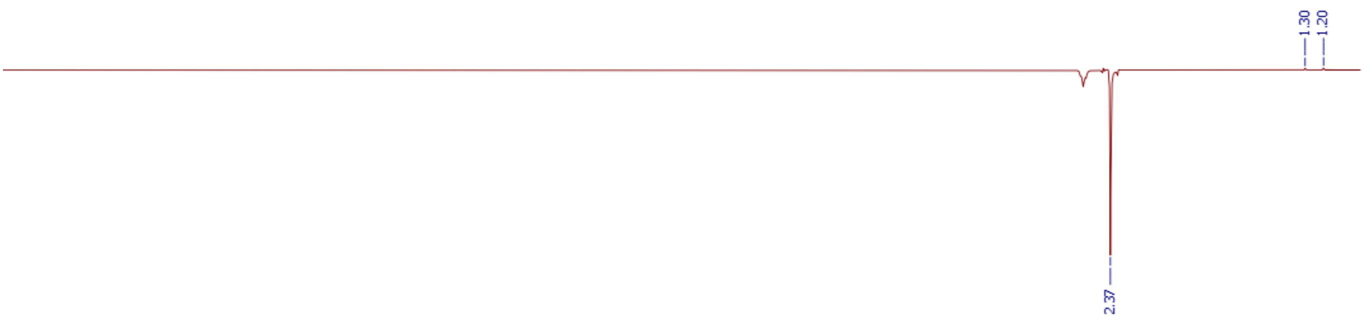

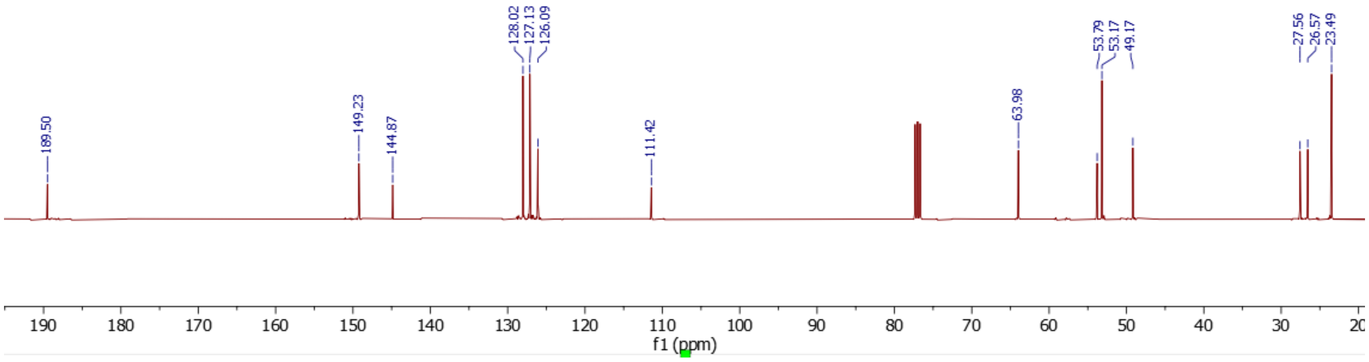

Cross-peak between C-4 and H-1’ in HMBC spektra:

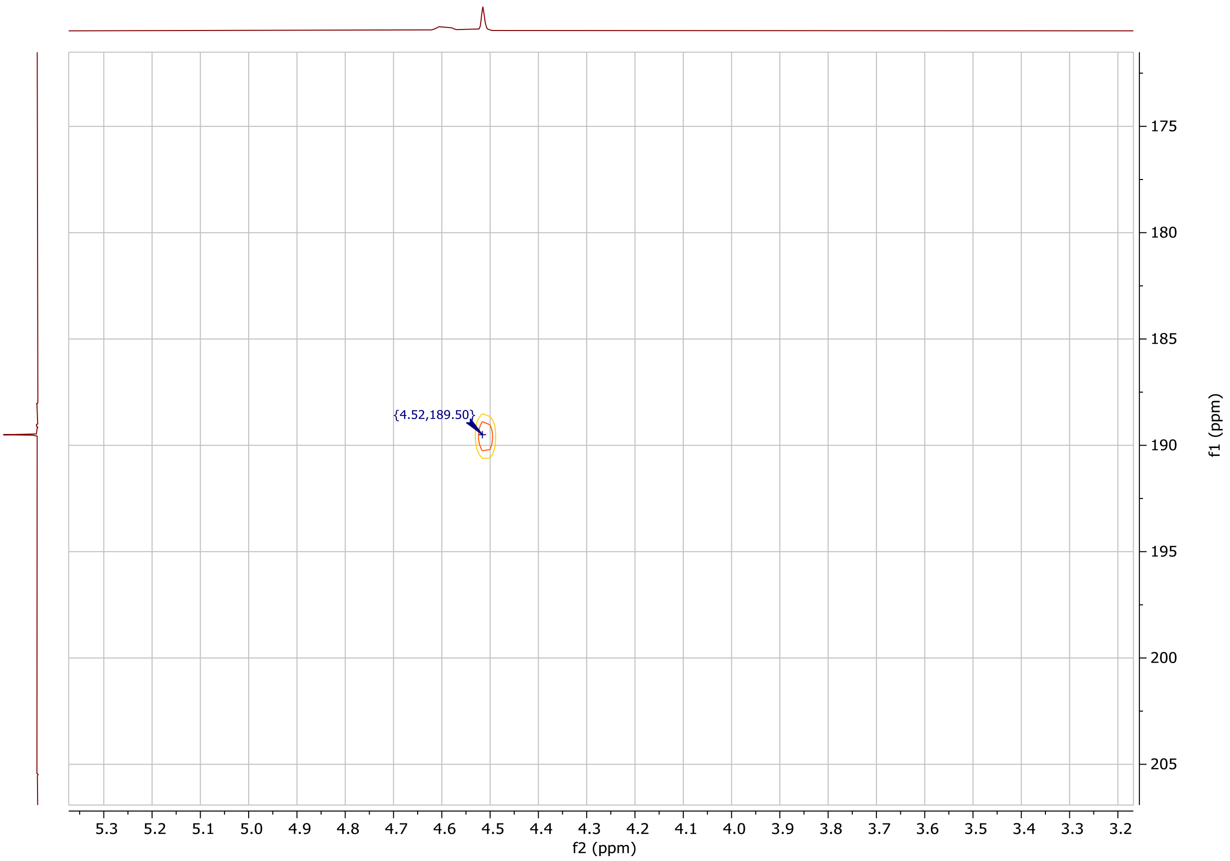

Cross-peak between C-5 and H-1’in HMBC spectra

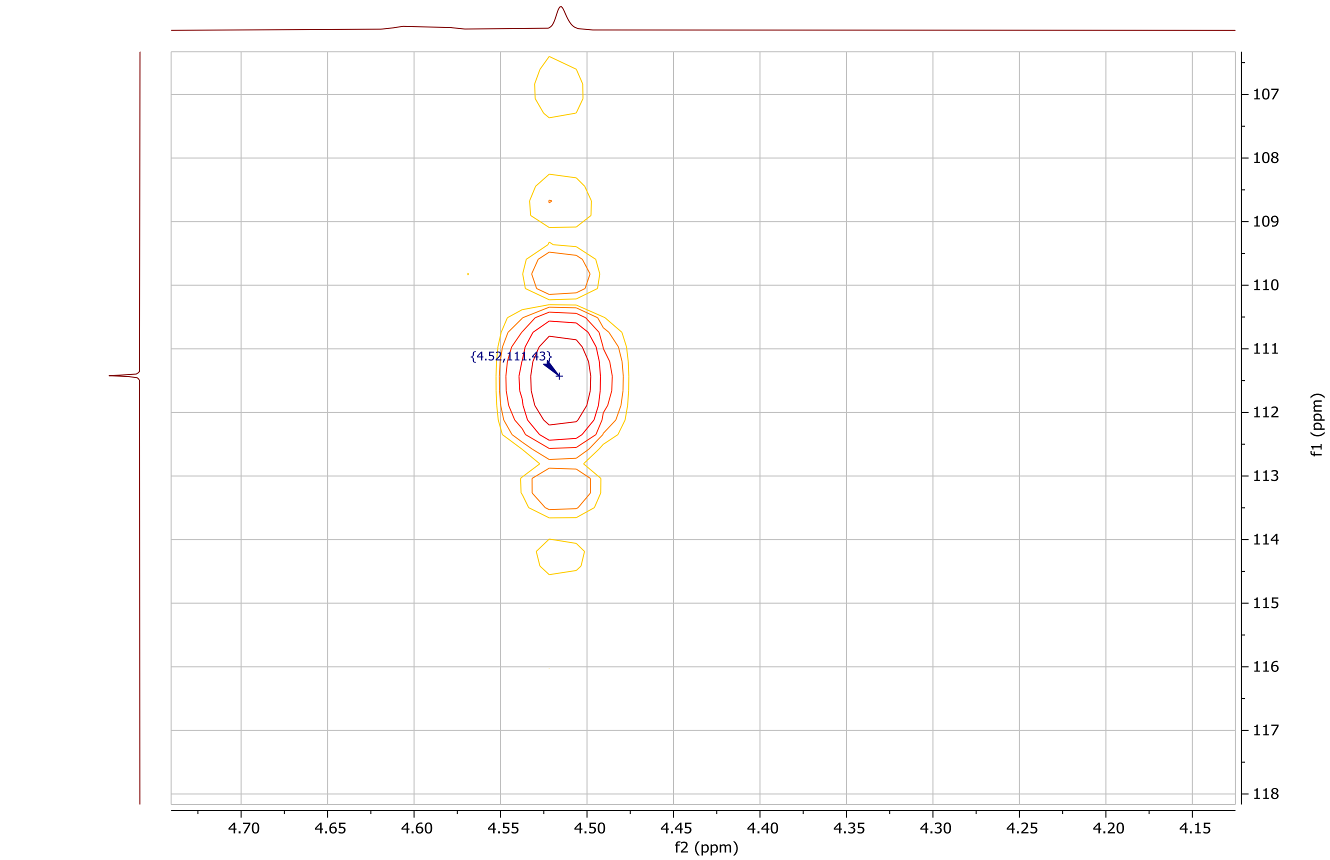

Cross-peak between NCH_2 and 1’-H in HMBC spectra

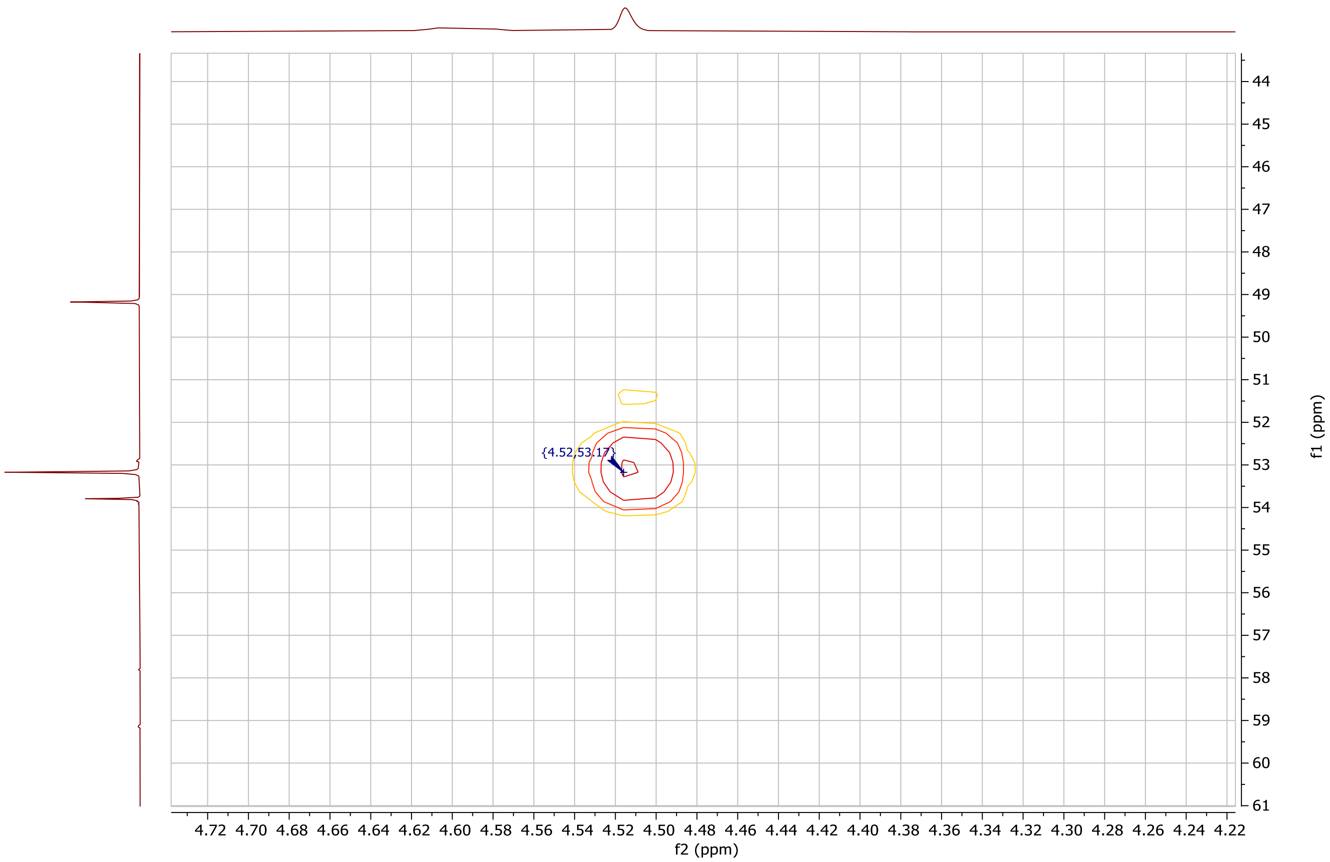

Sample: HMD95 Ionisation: DI-EI

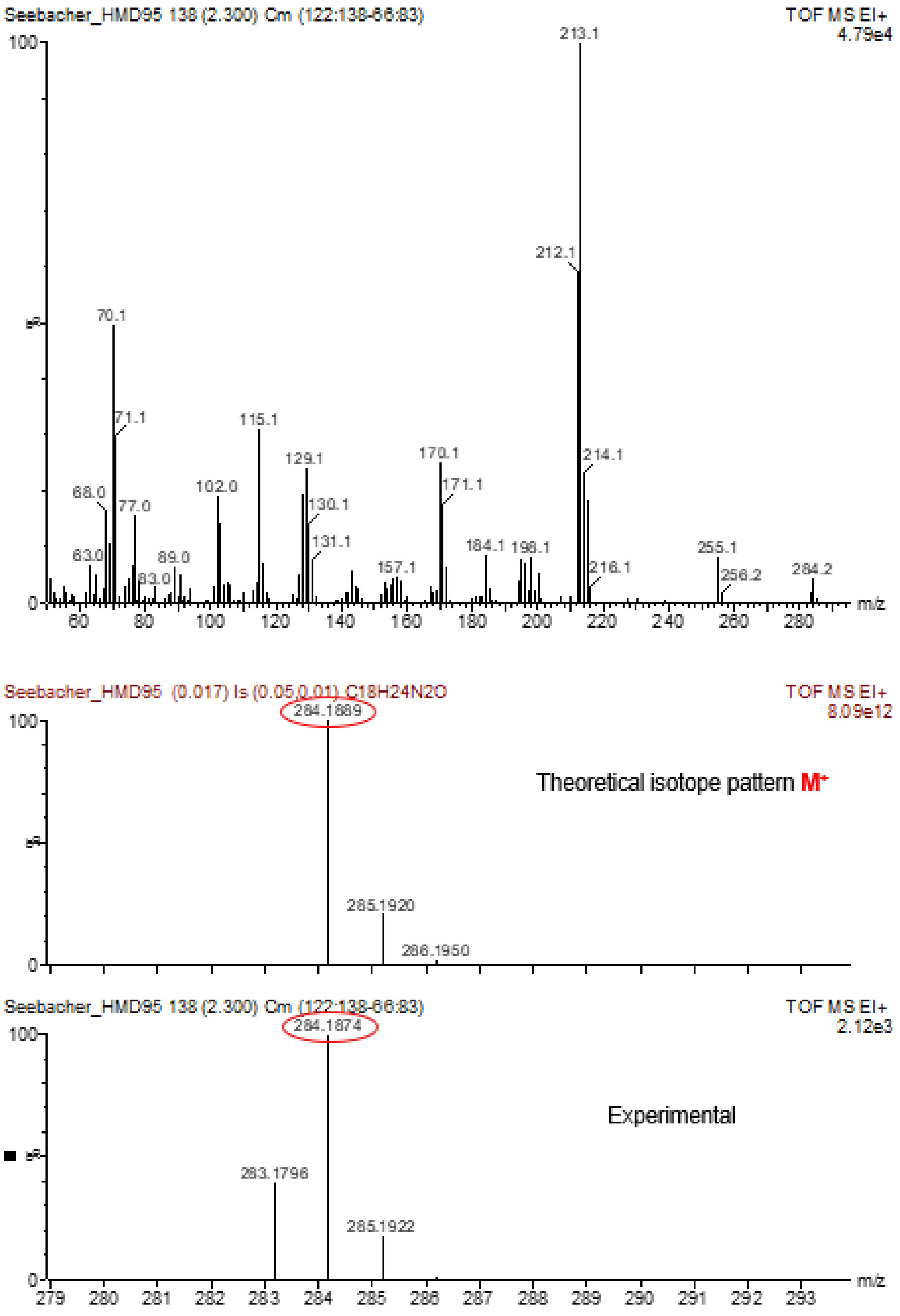

Figure S2. ^{1}H NMR at 400 MHz, ^{13}C NMR at 100 MHz spectra, and MS spectra for compound **2b**

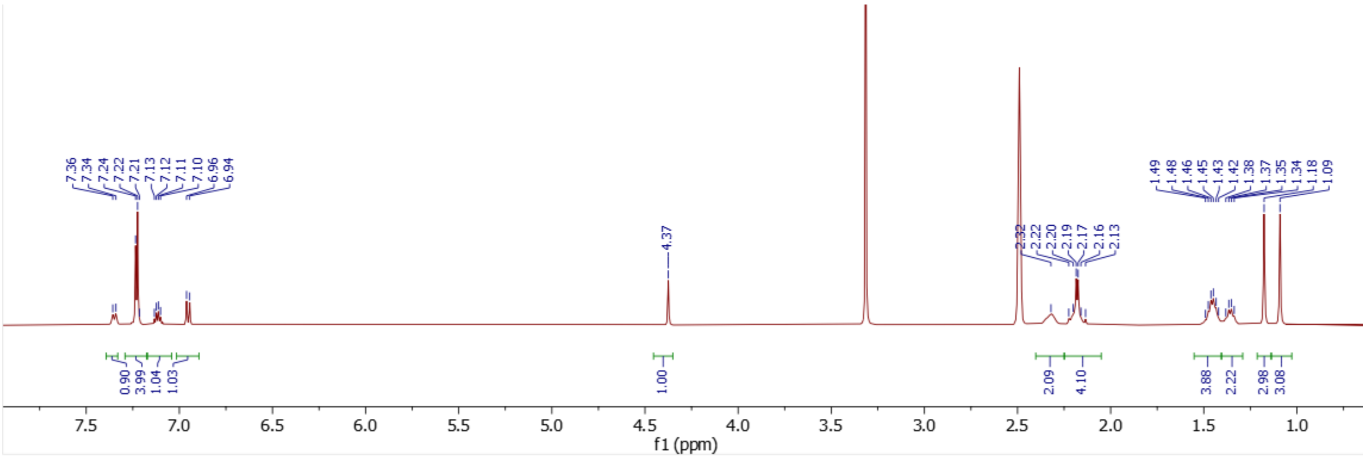

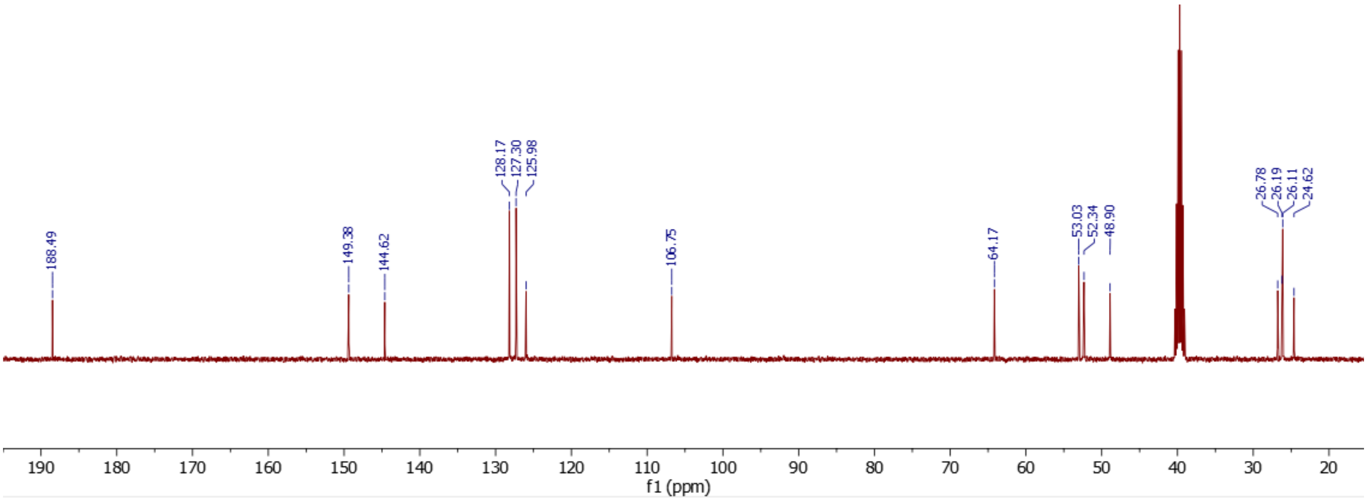

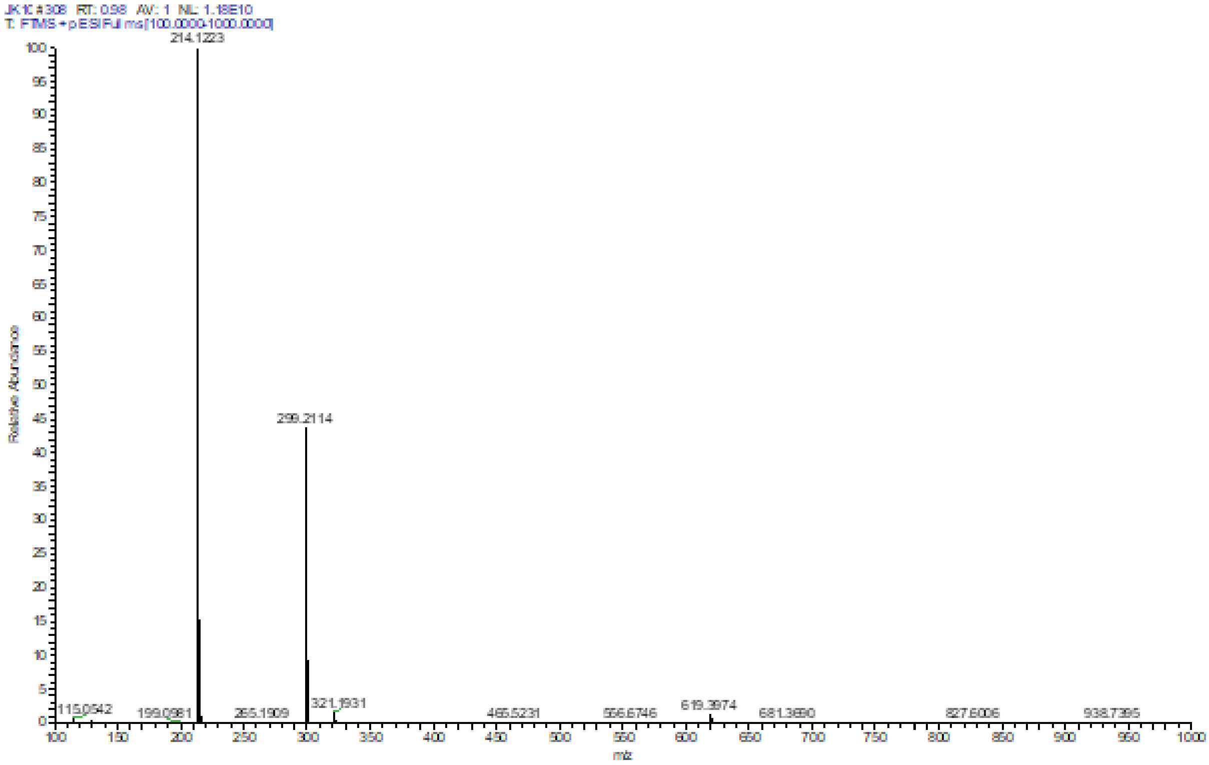

214.1223 [M+H-Piperidin]+

Figure S3. ^{1}H NMR at 400 MHz, ^{13}C NMR at 100 MHz spectra, and MS spectra for compound **2c**

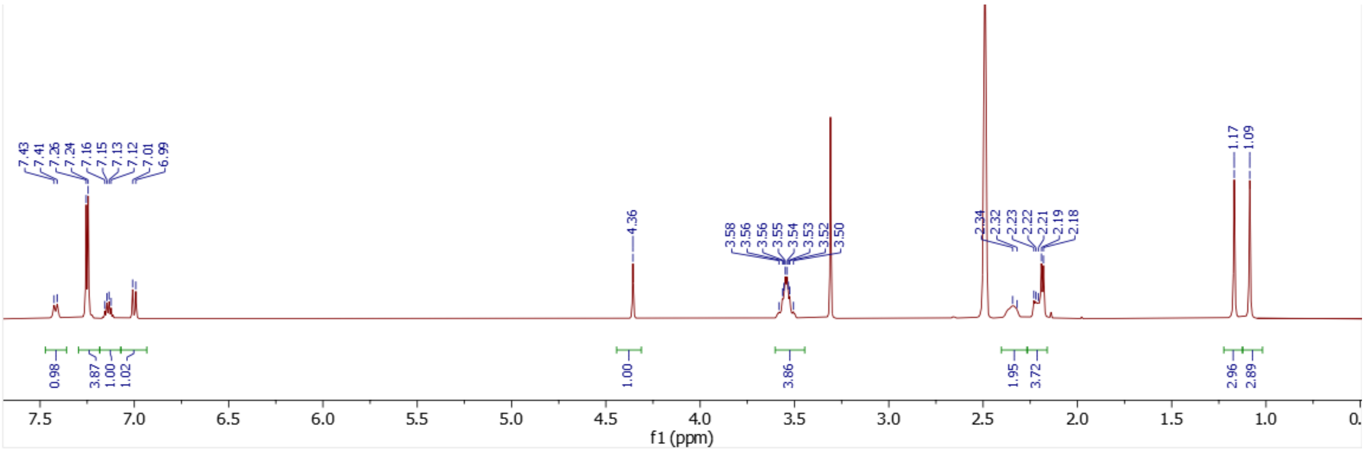

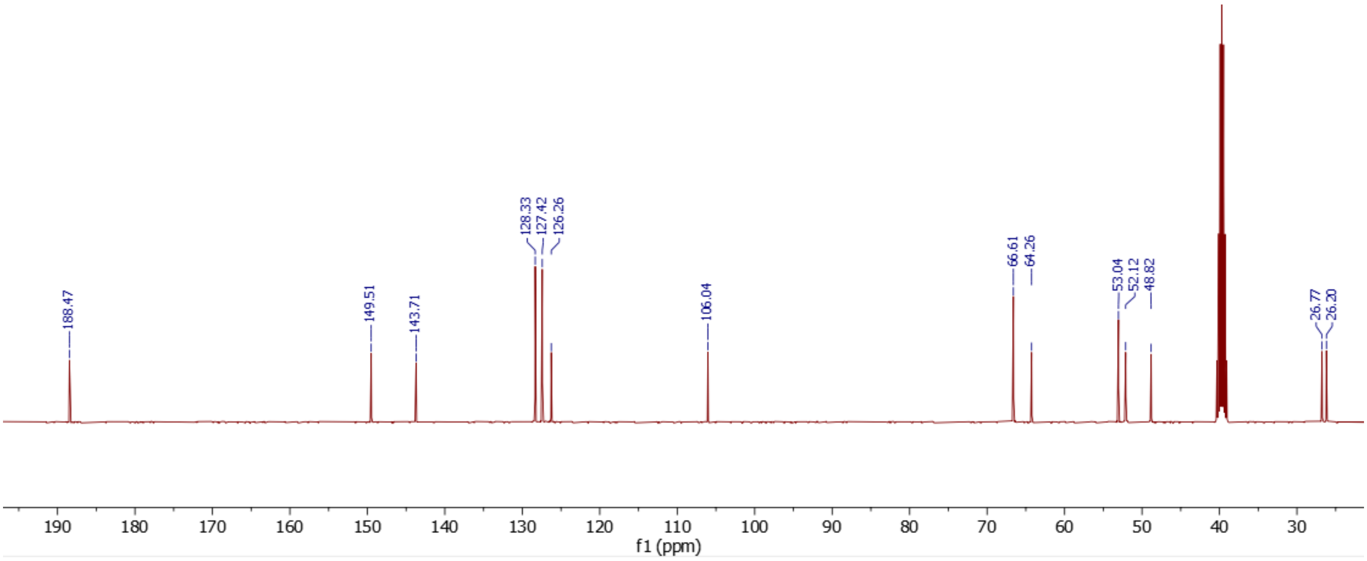

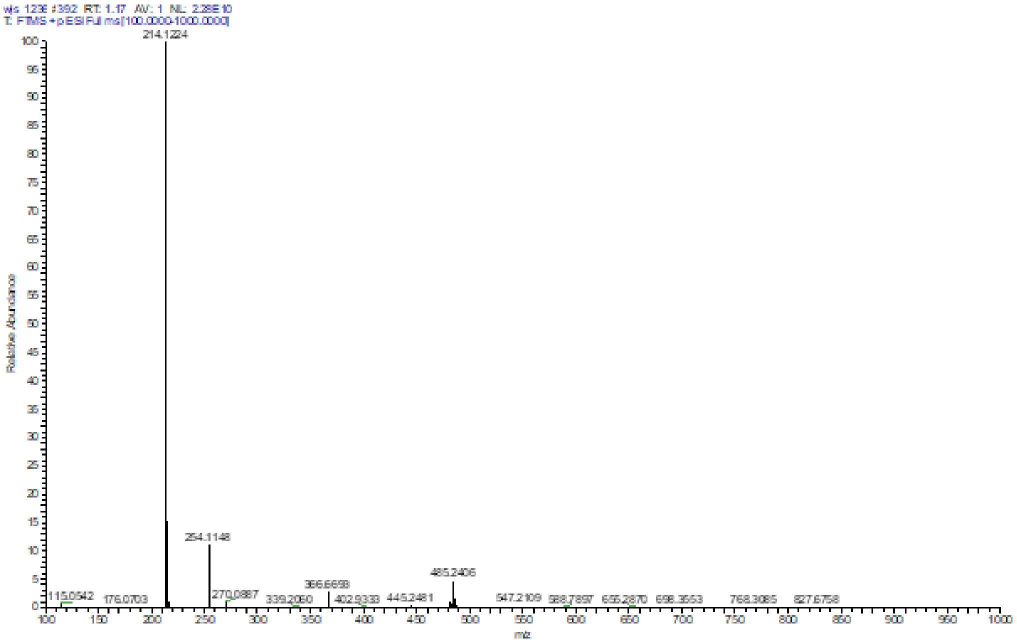

214.1224 = M+H-C4H9ON

Figure S4. ^{1}H NMR at 400 MHz, ^{13}C NMR at 100 MHz spectra, and MS spectra for compound **2d**

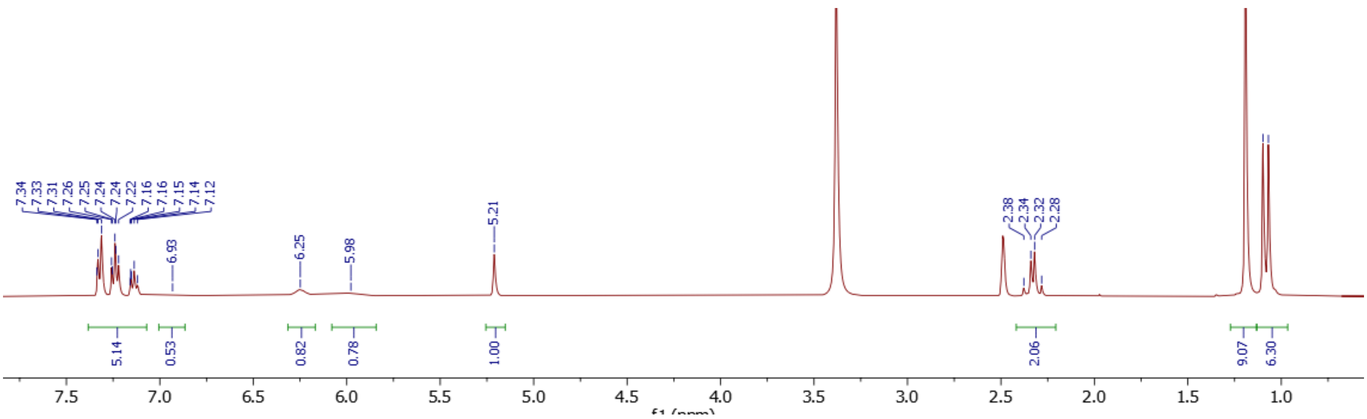

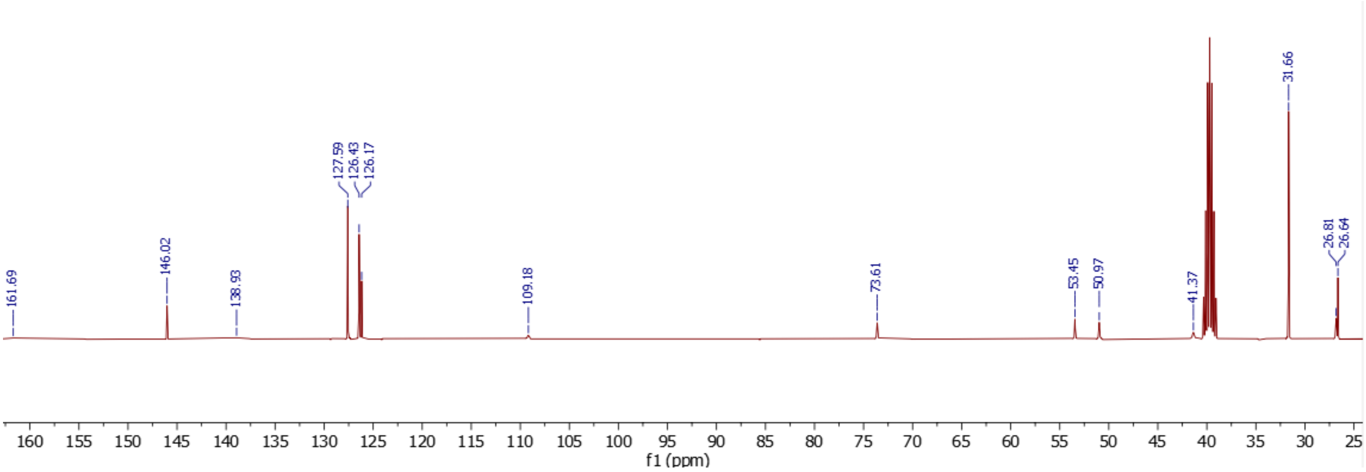

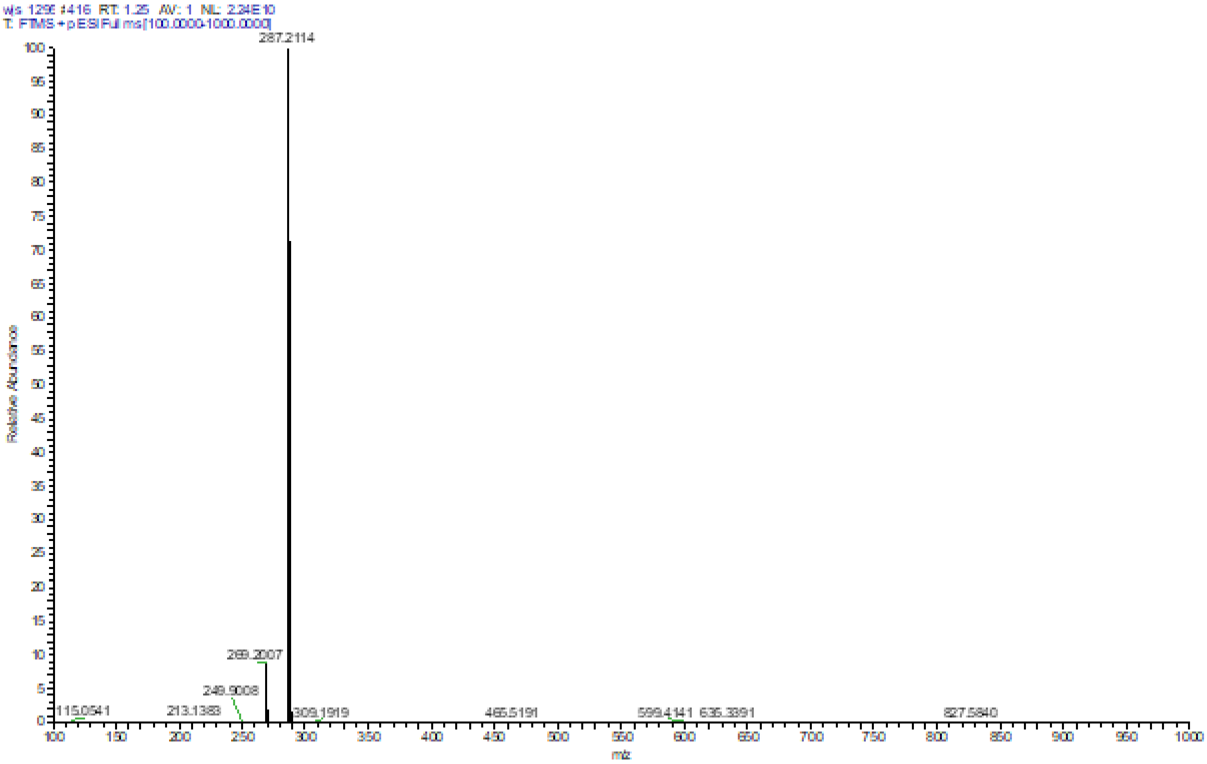

Figure S5. ^{1}H NMR at 400 MHz, ^{13}C NMR at 100 MHz spectra, and MS spectra for compound **3a**

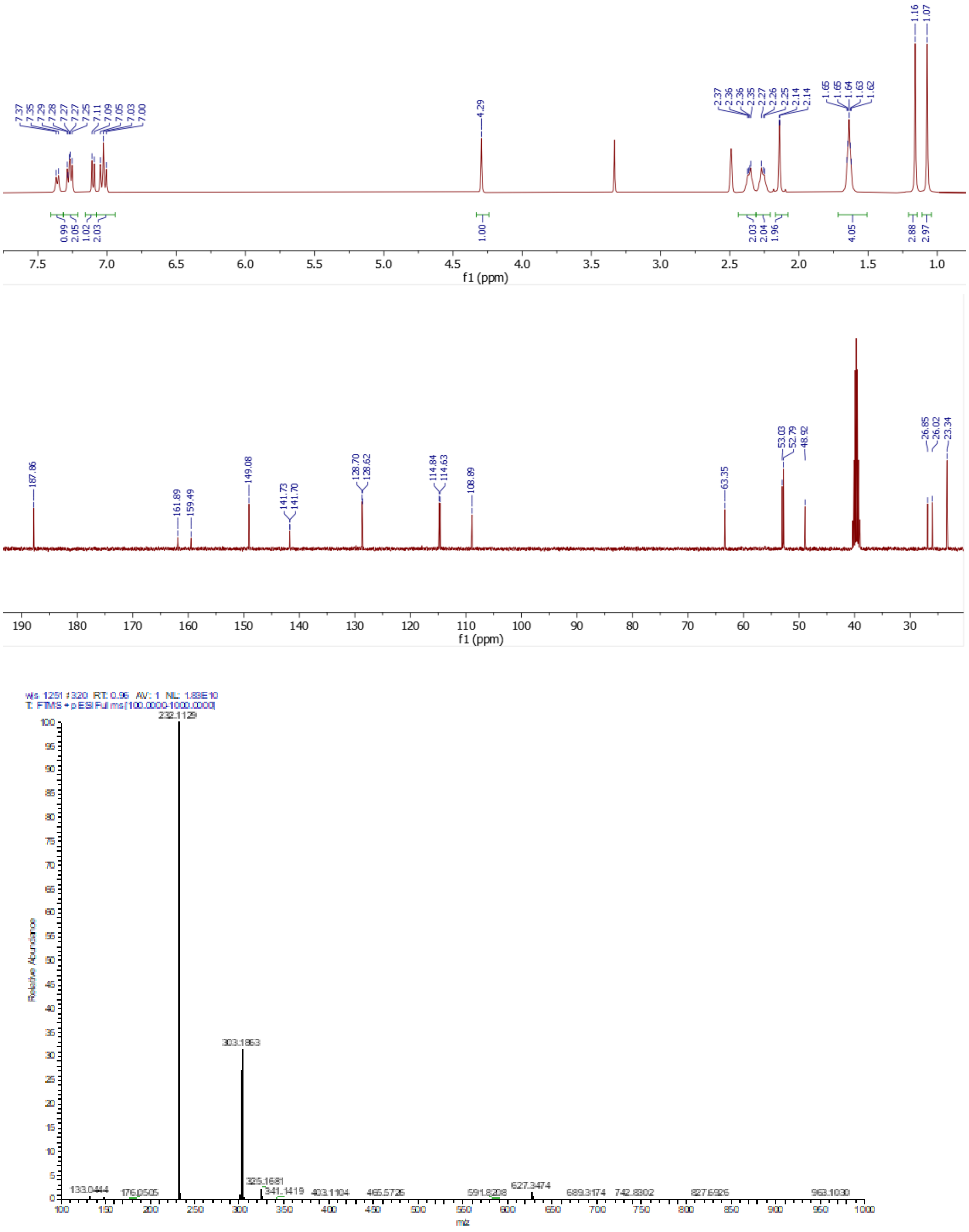

Figure S6. ^{1}H NMR at 400 MHz, ^{13}C NMR at 100 MHz spectra, and MS spectra for compound **3b**

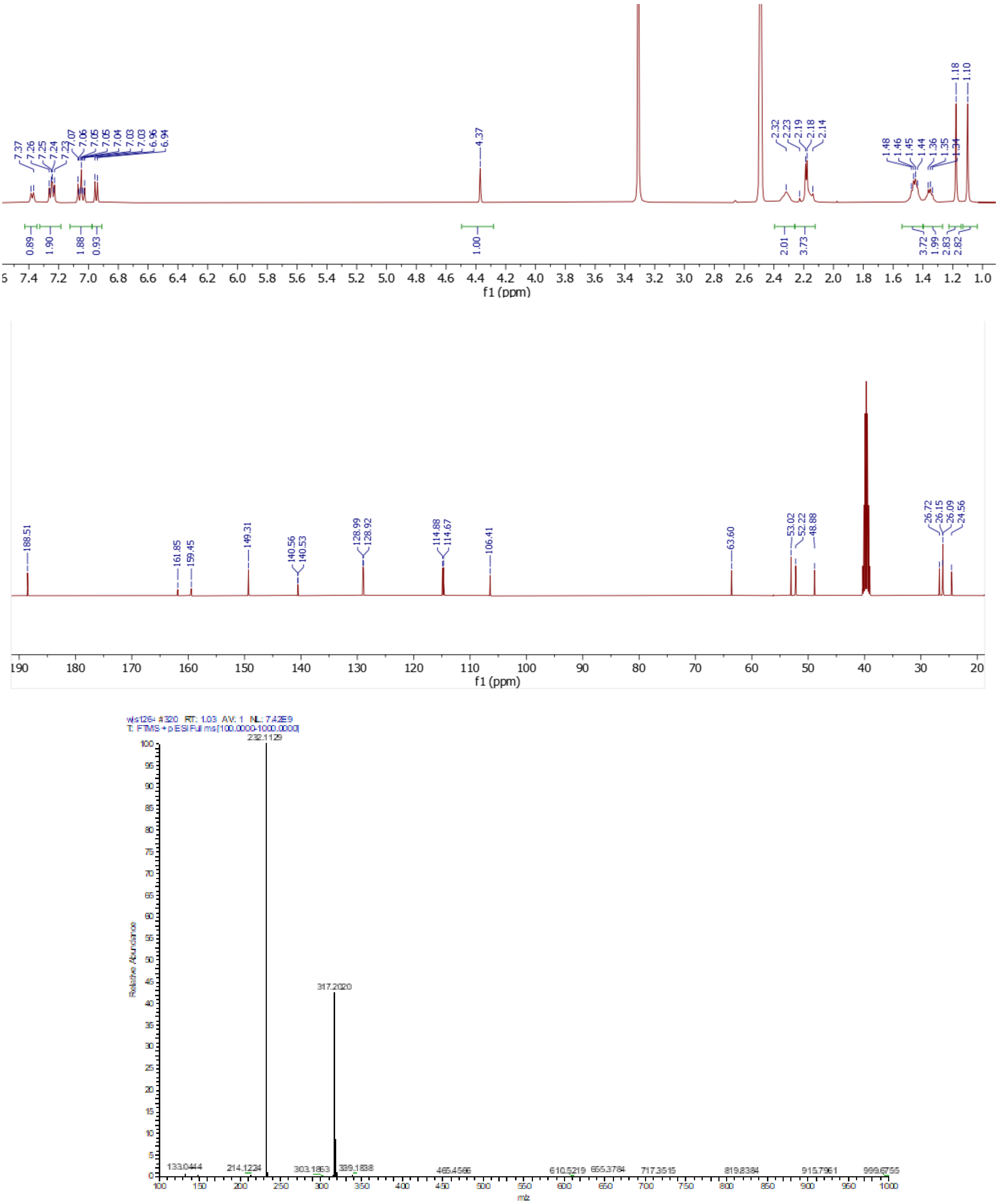

232.1129 [M+H-Piperidin]$^{+}$

Figure S7. ^{1}H NMR at 400 MHz, ^{13}C NMR at 100 MHz spectra, and MS spectra for compound **3c**

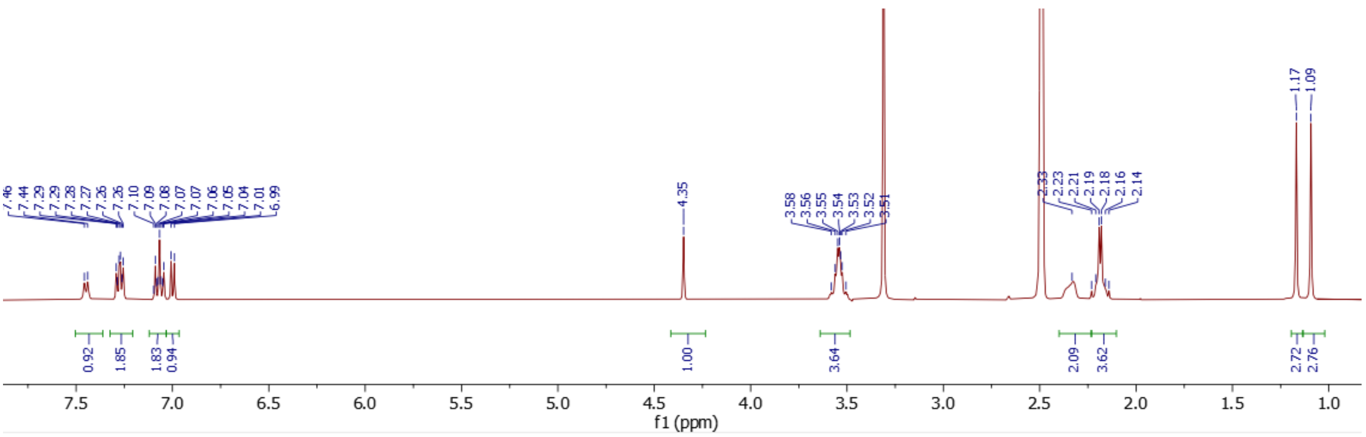

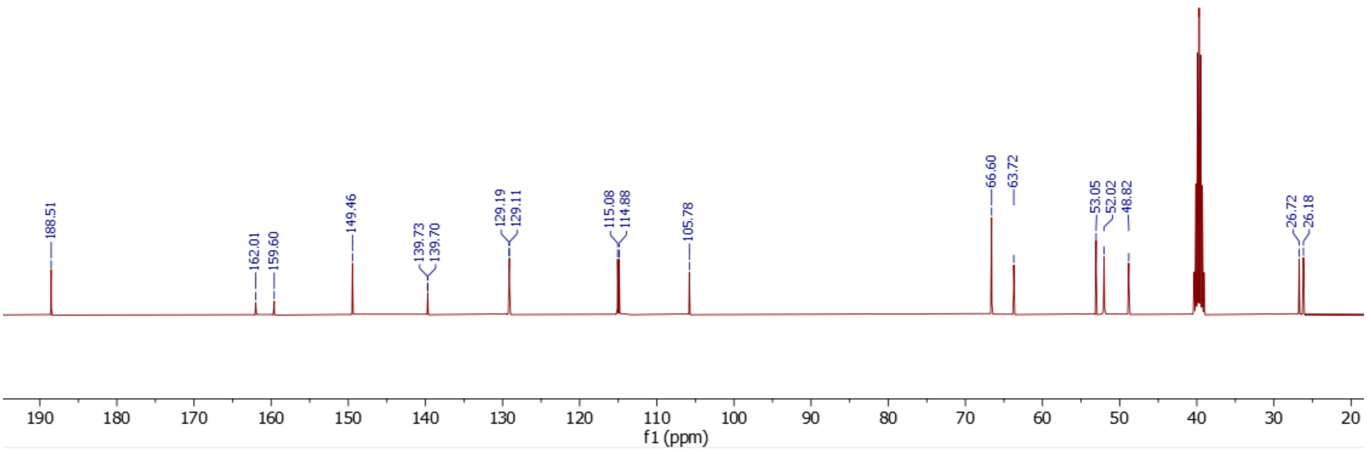

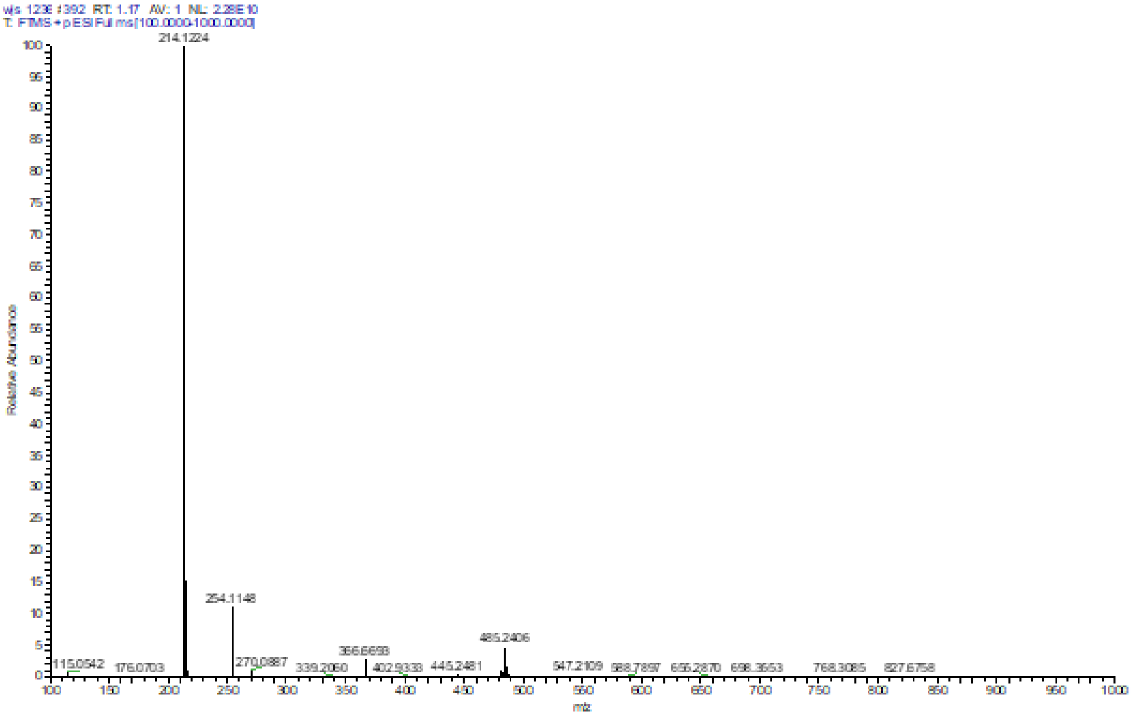

214= M+H-Morpholin

Figure S8. ^{1}H NMR at 400 MHz, ^{13}C NMR at 100 MHz spectra, and MS spectra for compound **4a**

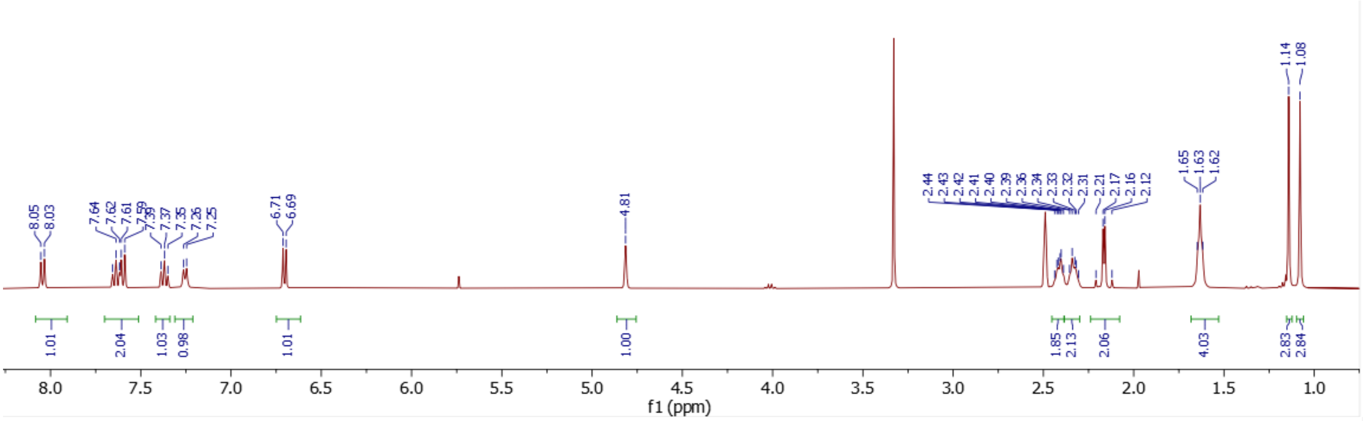

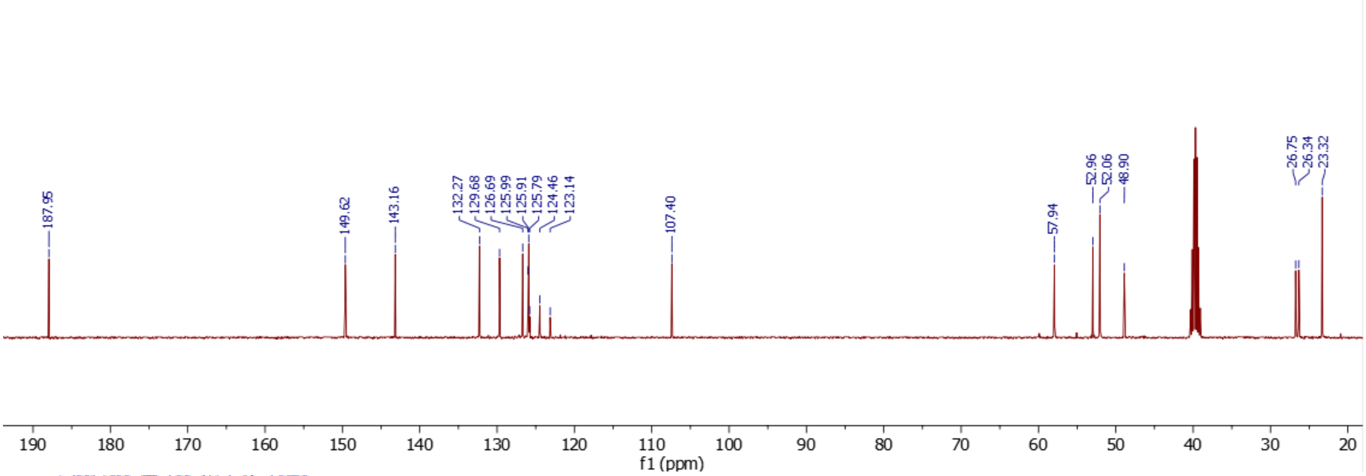

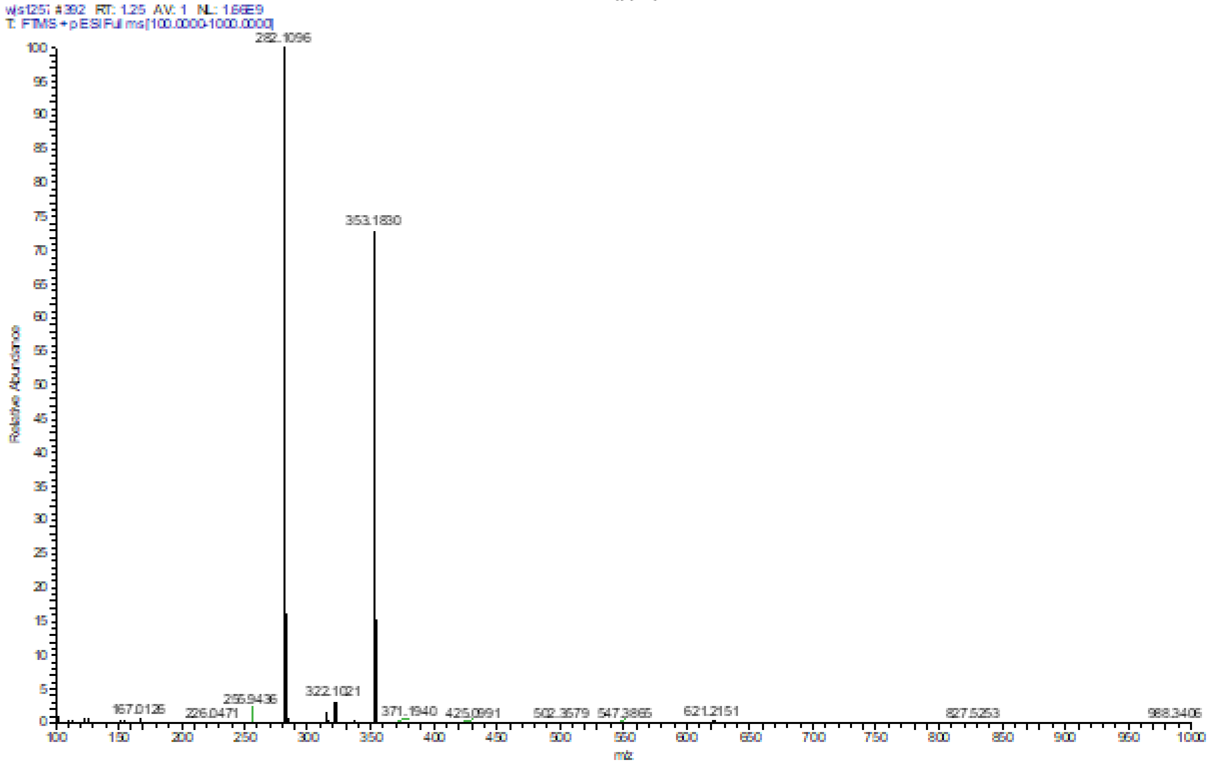

282.1096 [M+H-Pyrrolidin]$^{+}$

Figure S9. ^{1}H NMR at 400 MHz, ^{13}C NMR at 100 MHz spectra, and MS spectra for compound **4b**

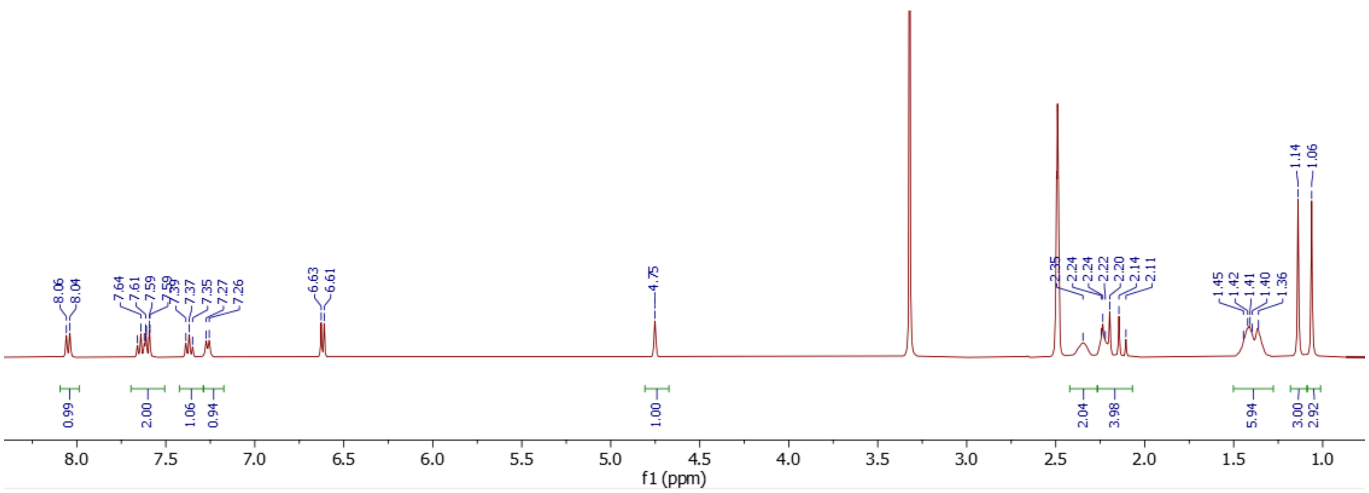

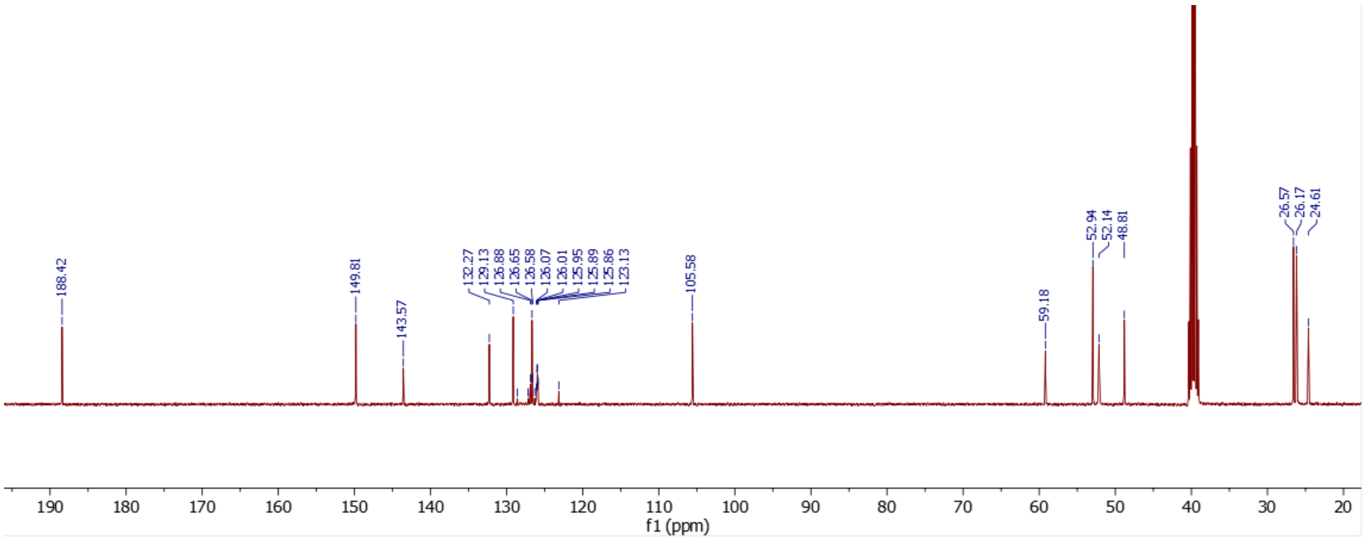

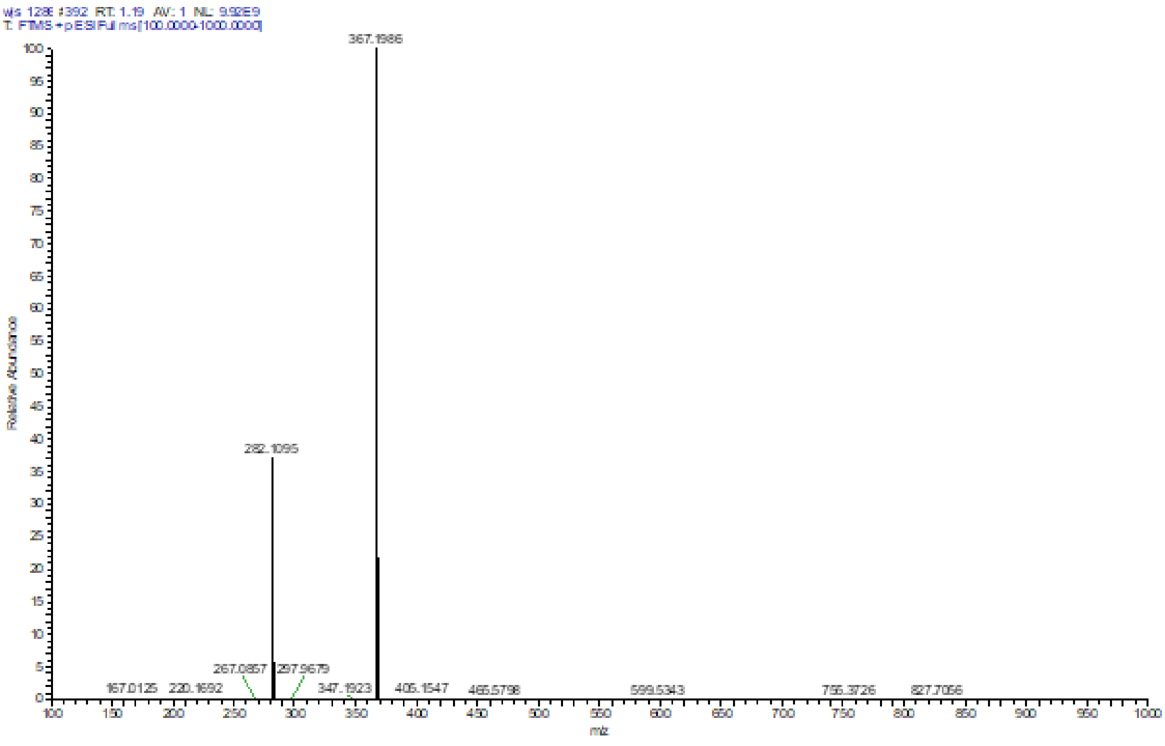

282 = M+H-Piperidin

Figure S10. ^{1}H NMR at 400 MHz, ^{13}C NMR at 100 MHz spectra, and MS spectra for compound **5a**

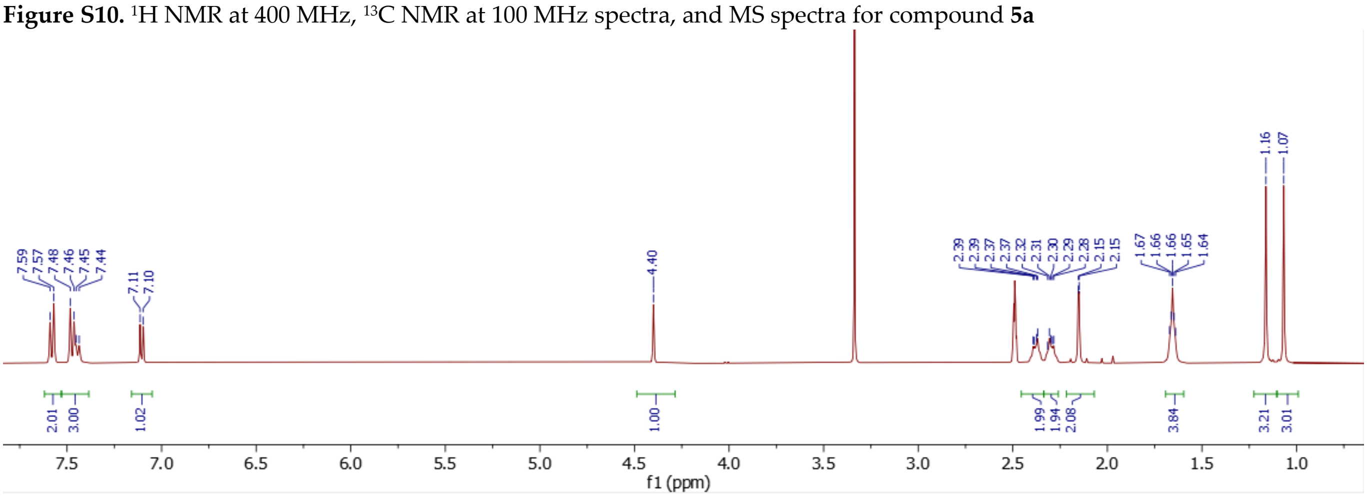

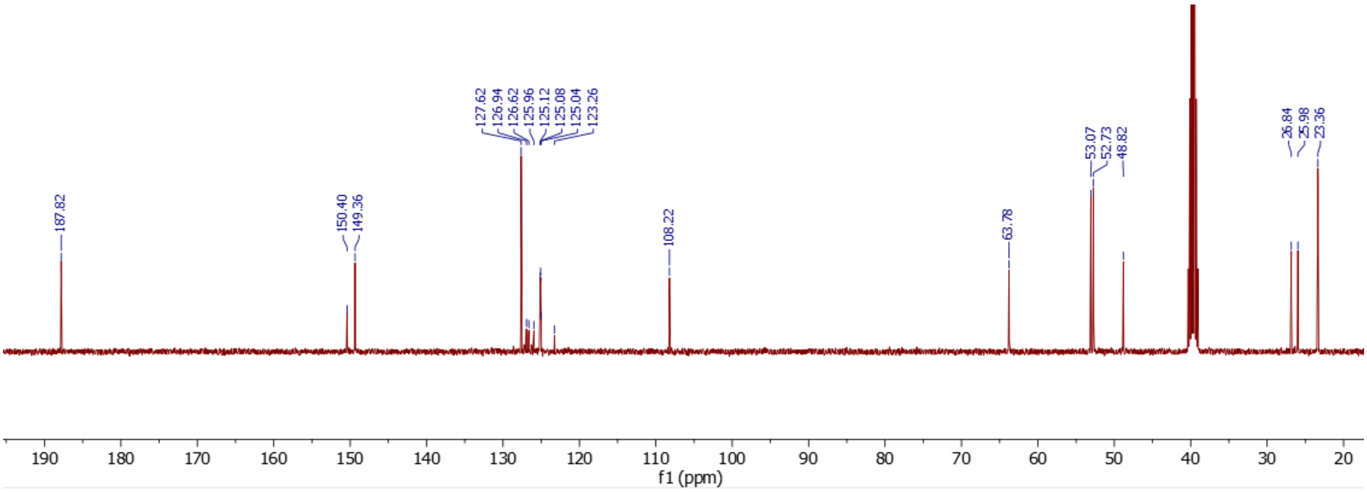

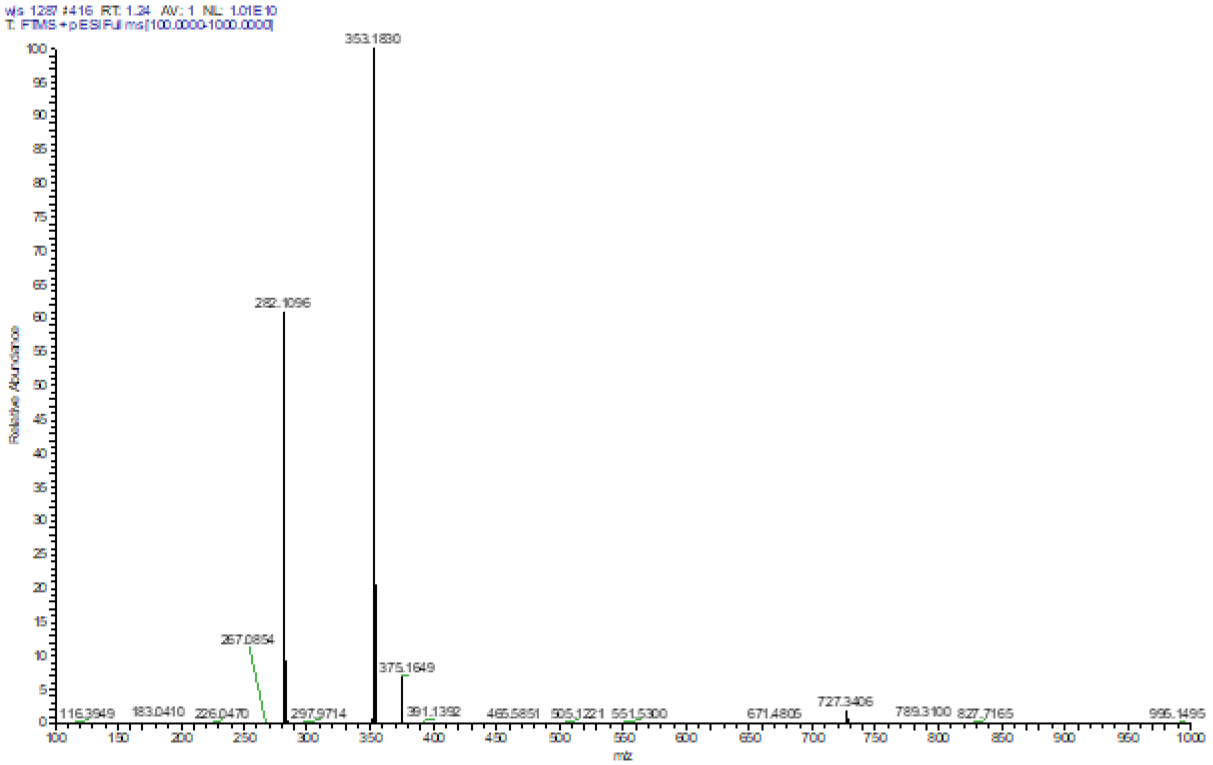

282= M+H-Pyrrolidin

Figure S11. ^{1}H NMR at 400 MHz, ^{13}C NMR at 100 MHz spectra, and MS spectra for compound **5b**

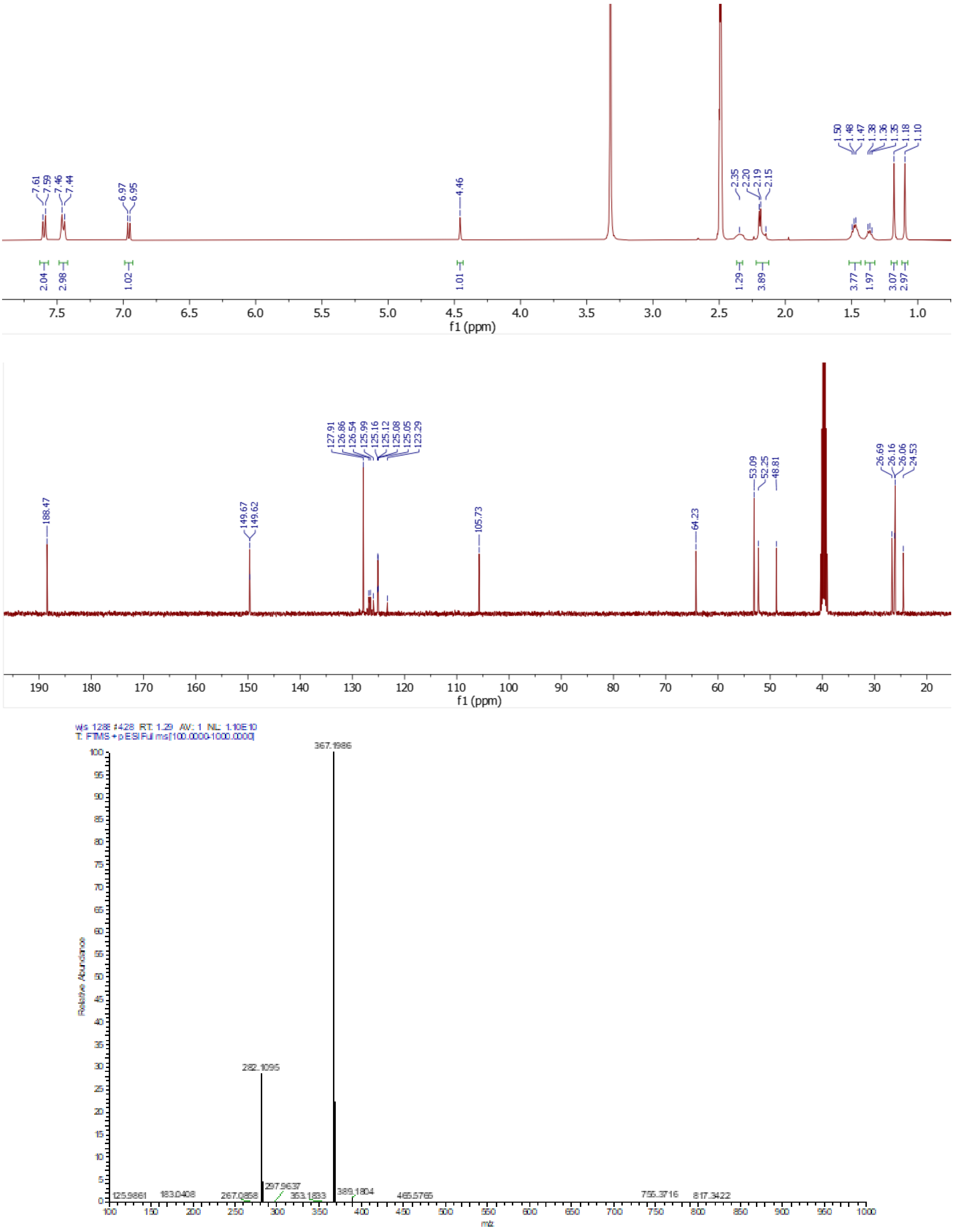

282= M+H-Piperidin

Figure S12. ^{1}H NMR at 400 MHz, ^{13}C NMR at 100 MHz spectra, and MS spectra for compound **6a**

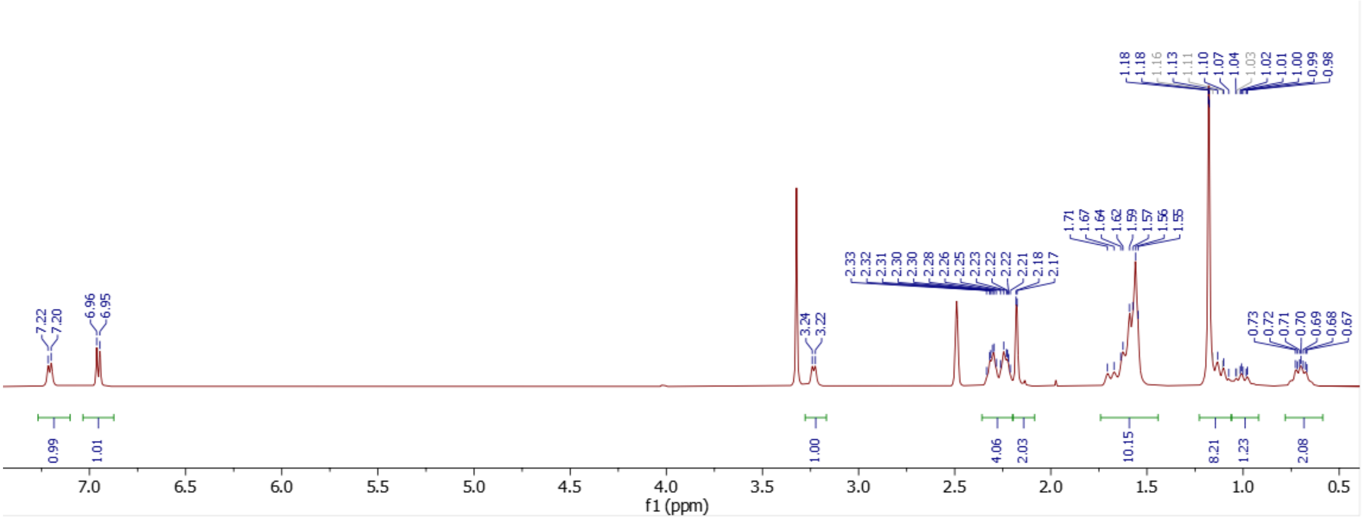

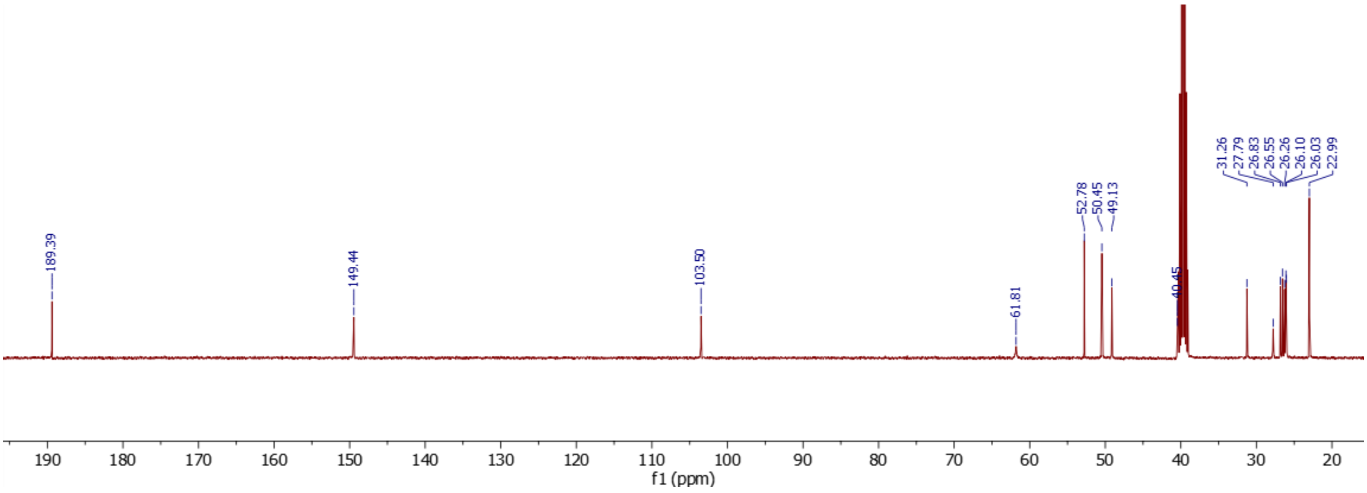

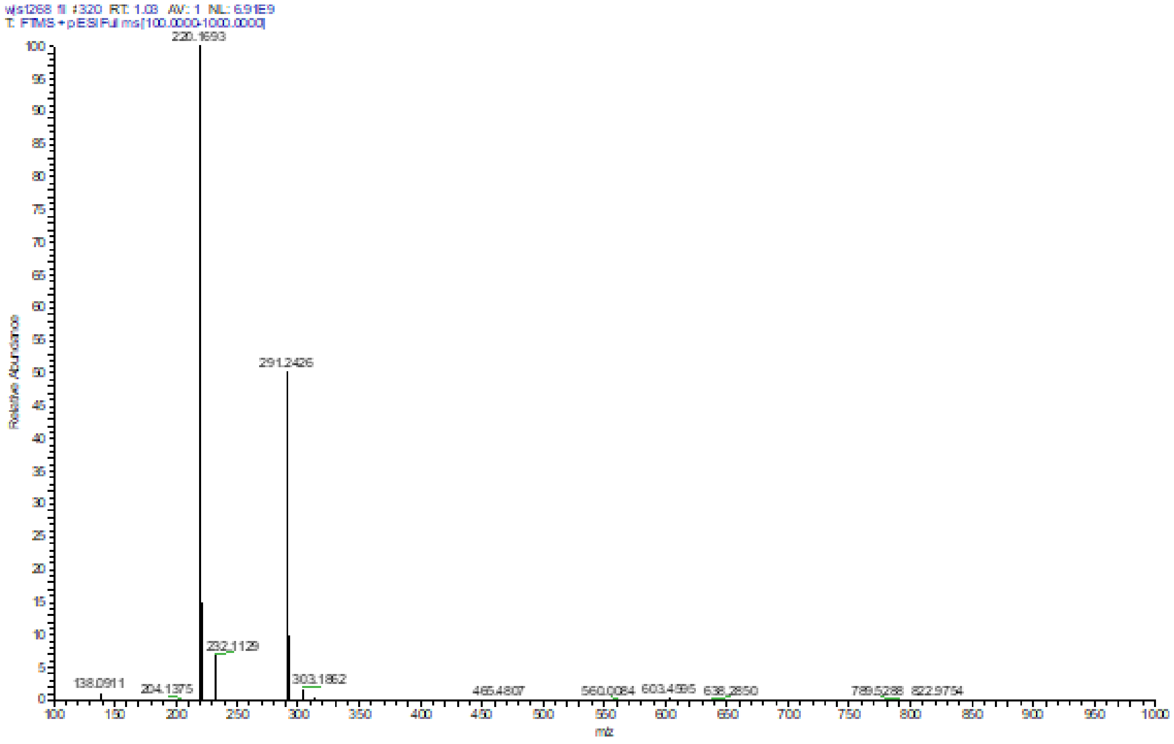

230.1693 [M+H-Pyrrolidin]$^{+}$

Figure S13. ^{1}H NMR at 400 MHz, ^{13}C NMR at 100 MHz spectra, and MS spectra for compound **6b**

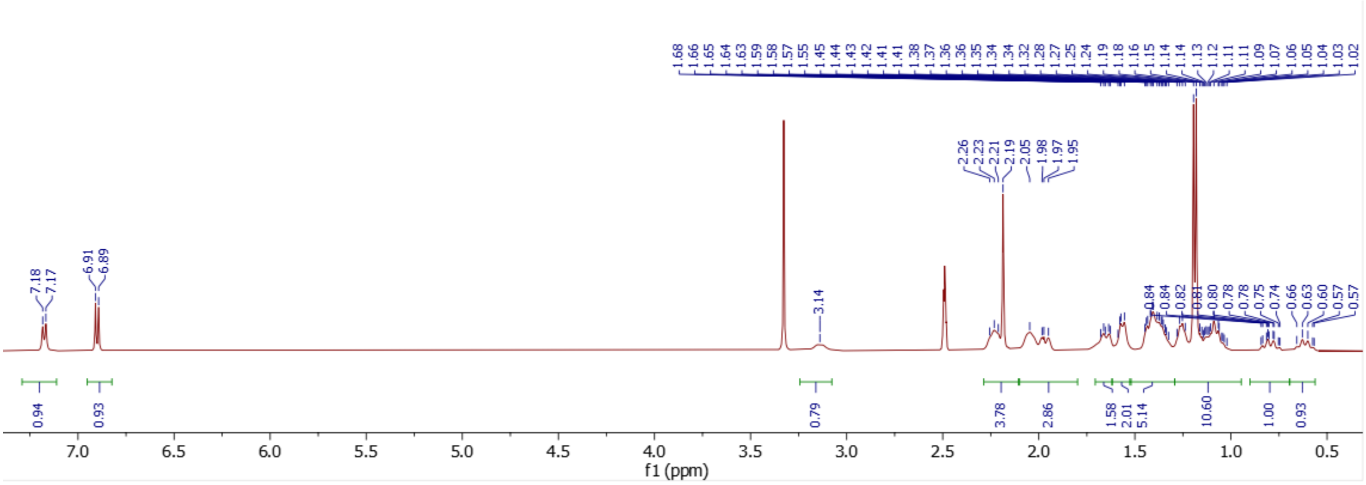

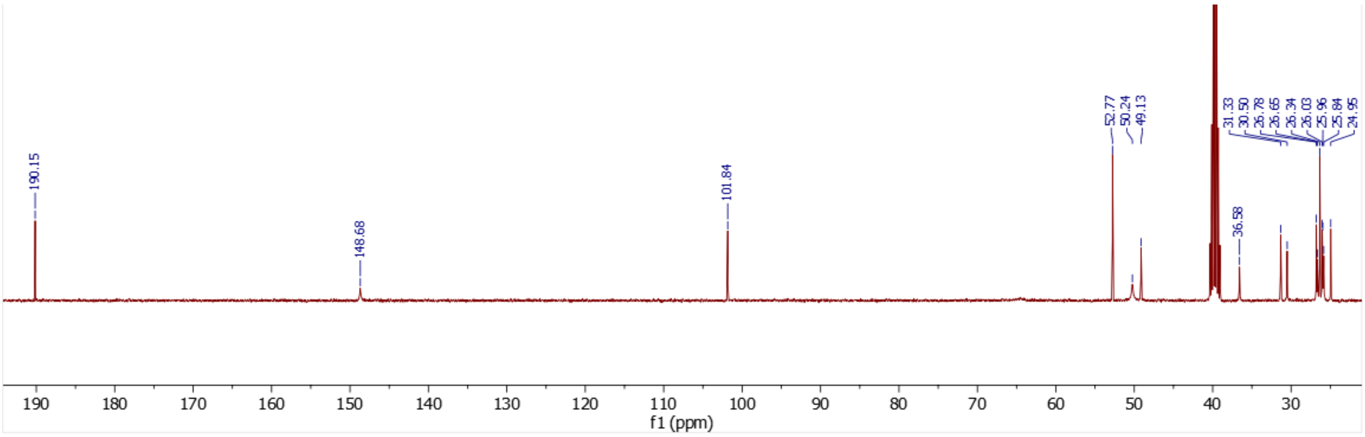

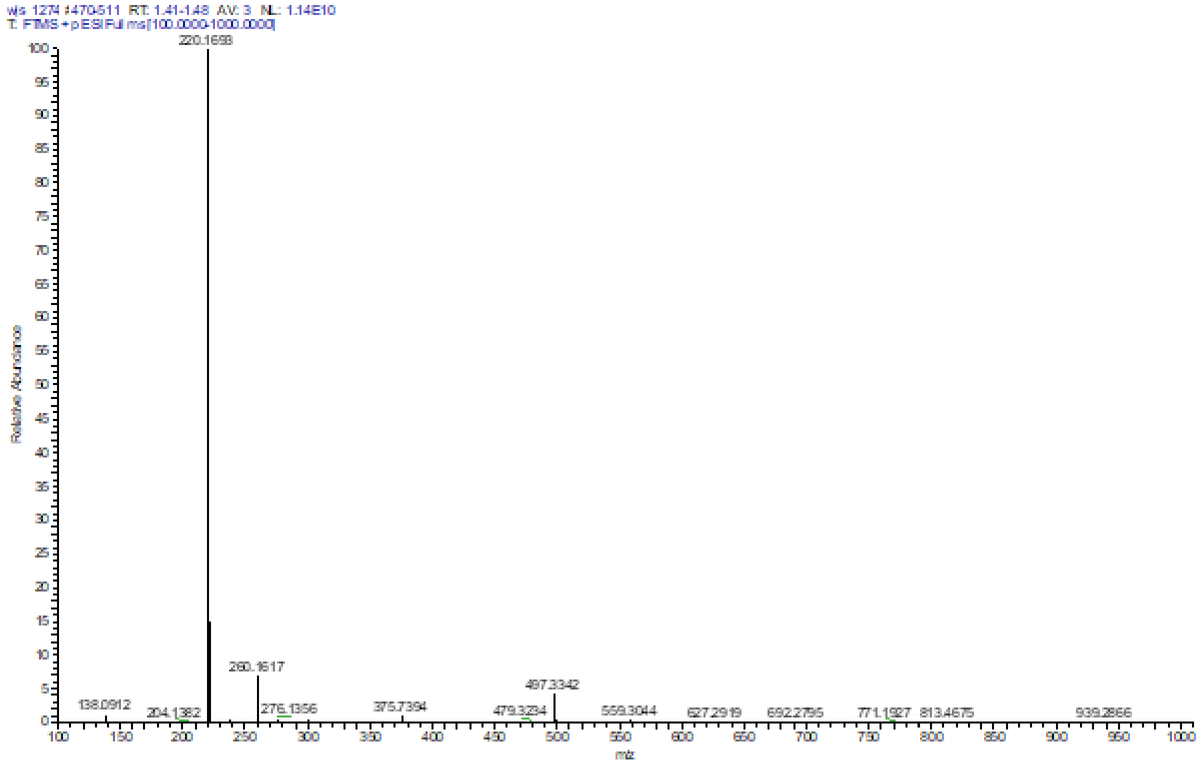

M+H-Piperidin

Figure S14. ^{1}H NMR at 400 MHz, ^{13}C NMR at 100 MHz spectra, and MS spectra for compound **7a**

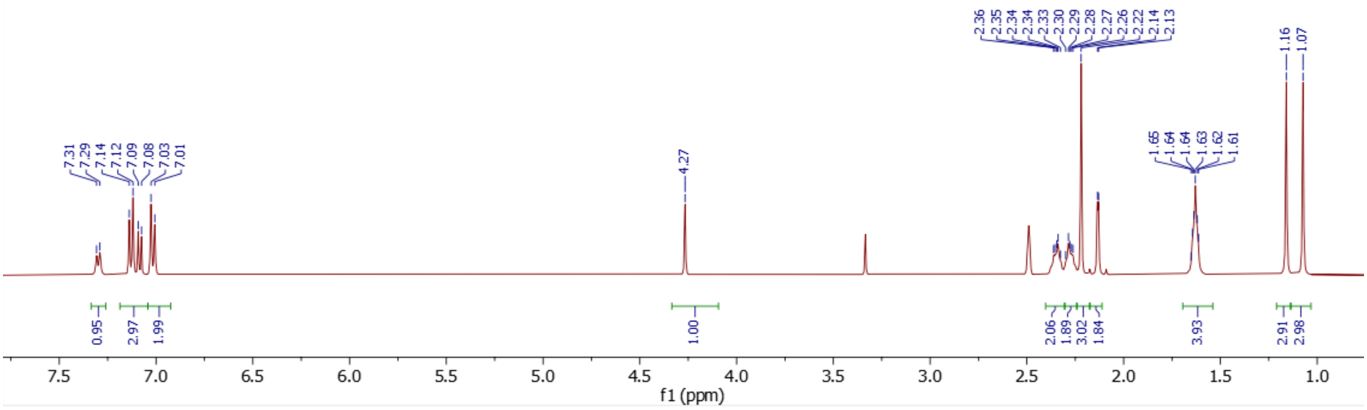

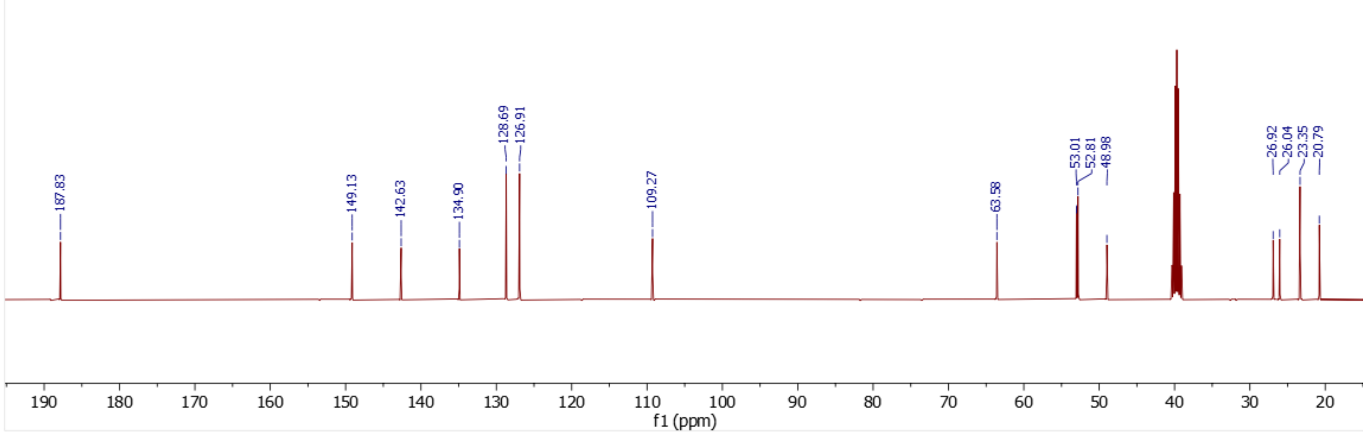

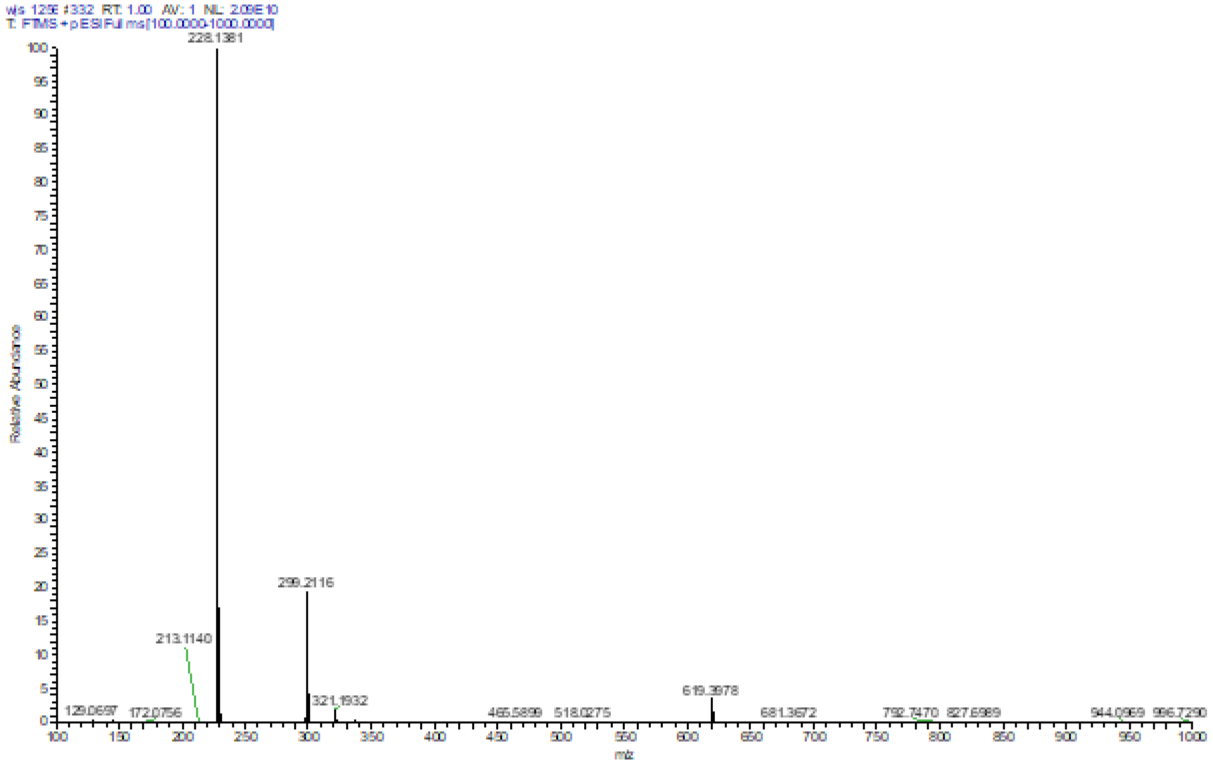

228= M+H-Pyrrolidin

Figure S15. ^{1}H NMR at 400 MHz, ^{13}C NMR at 100 MHz spectra, and MS spectra for compound **8a**

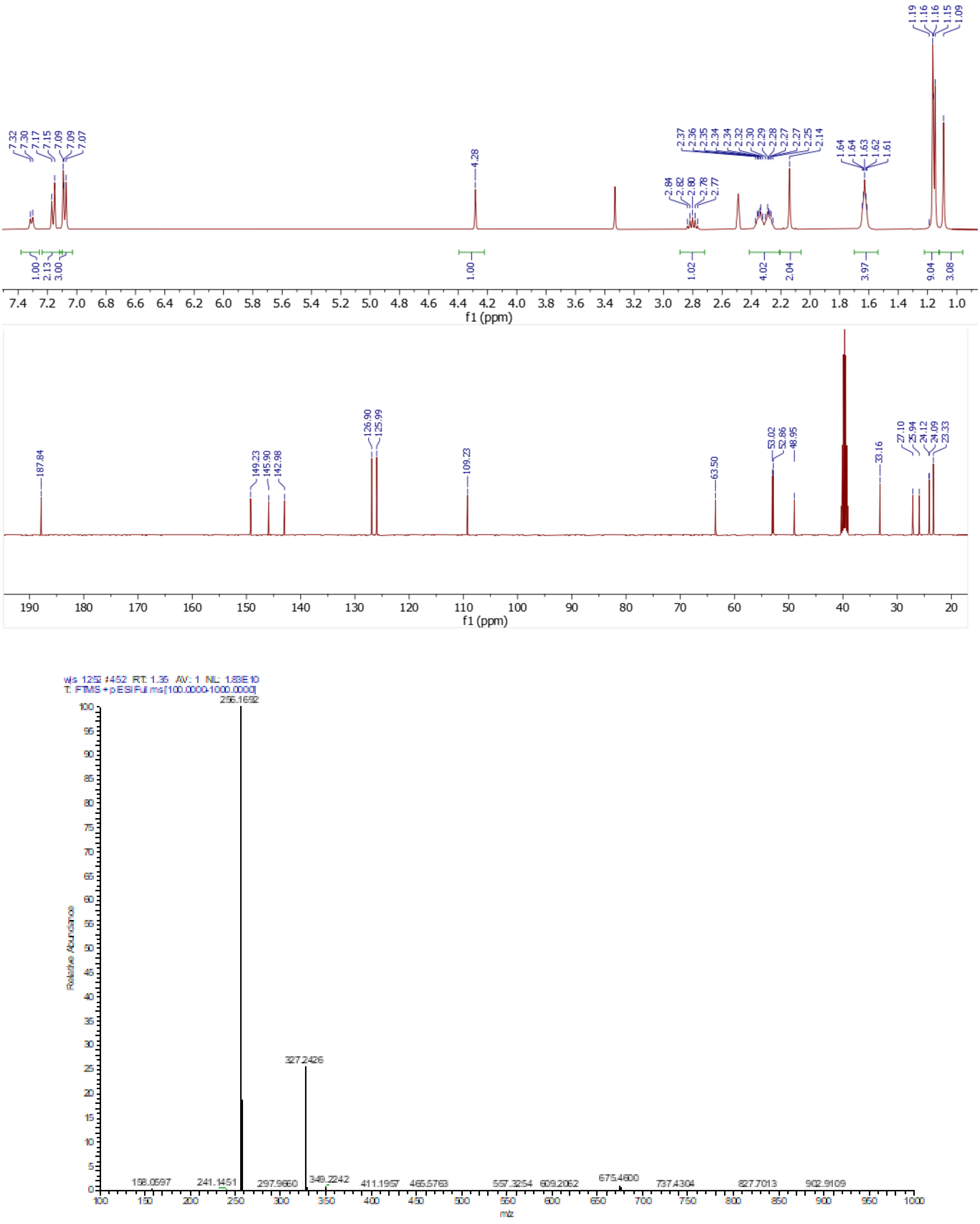

Figure S16. ^{1}H NMR at 400 MHz, ^{13}C NMR at 100 MHz spectra, and MS spectra for compound **9a**

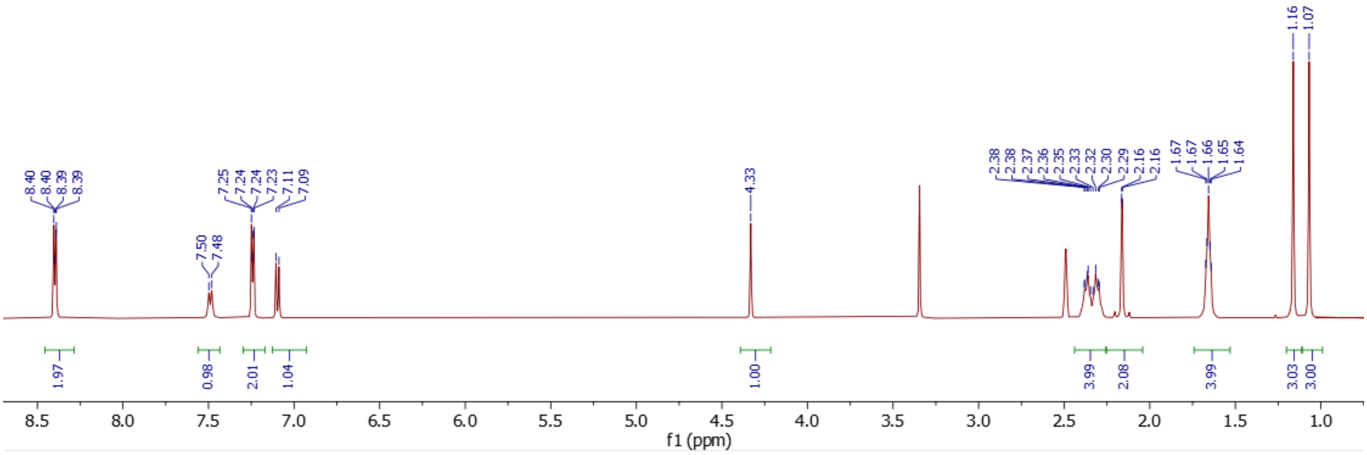

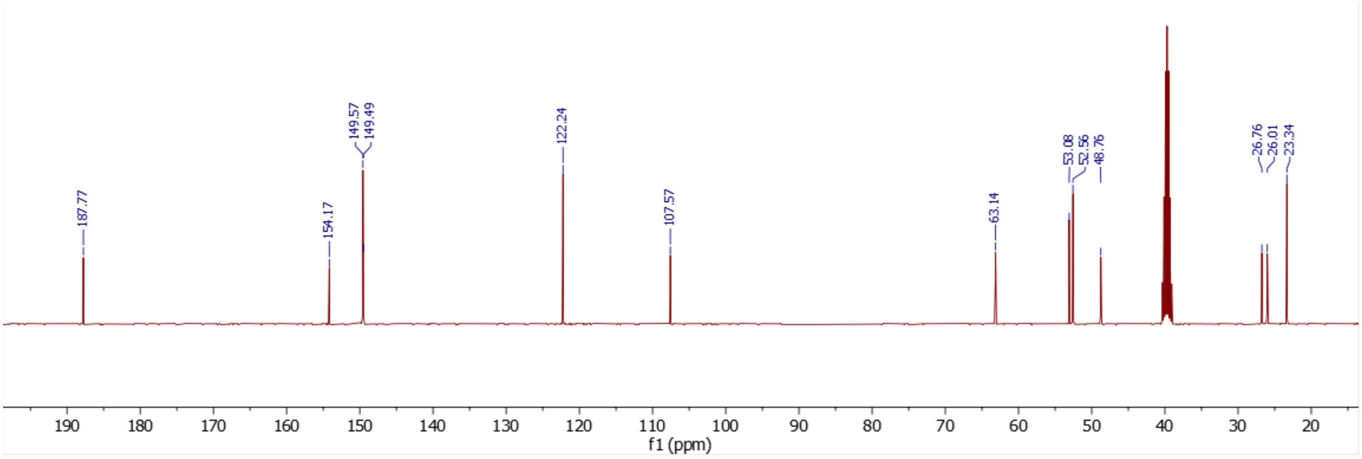

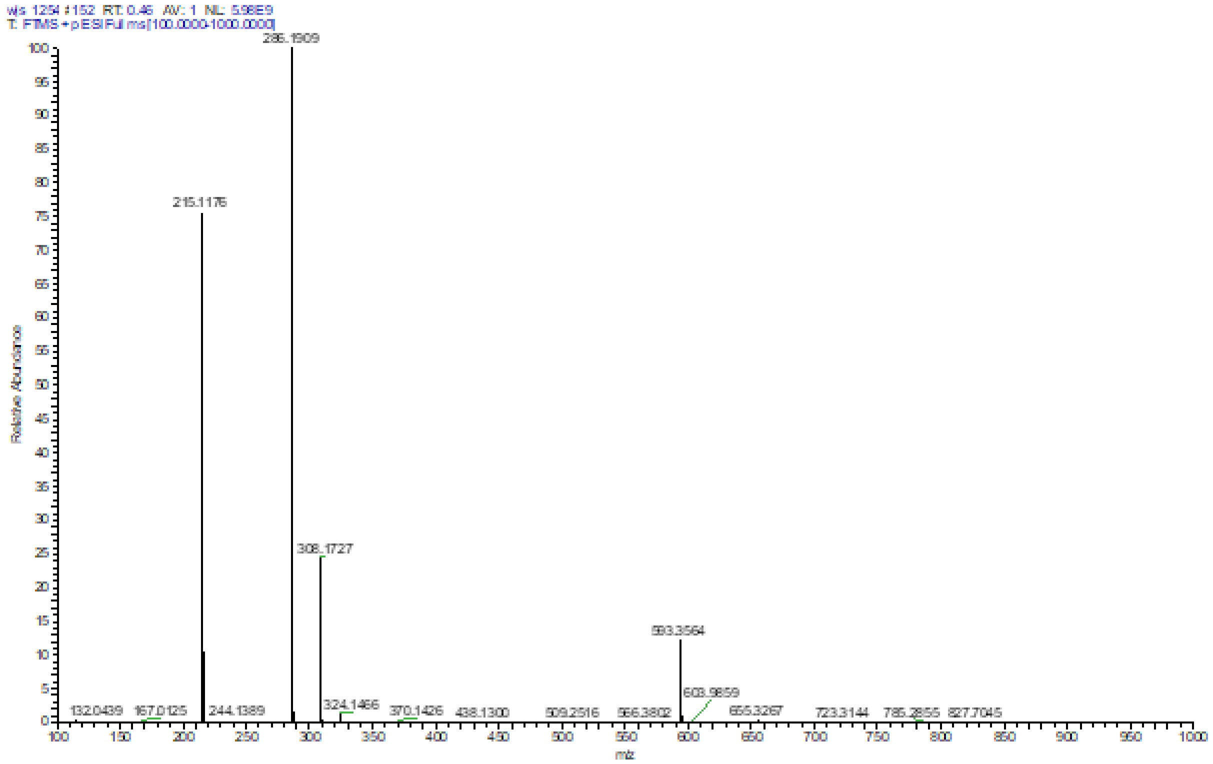

Figure S17. ^{1}H NMR at 400 MHz, ^{13}C NMR at 100 MHz spectra, and MS spectra for compound **10a**

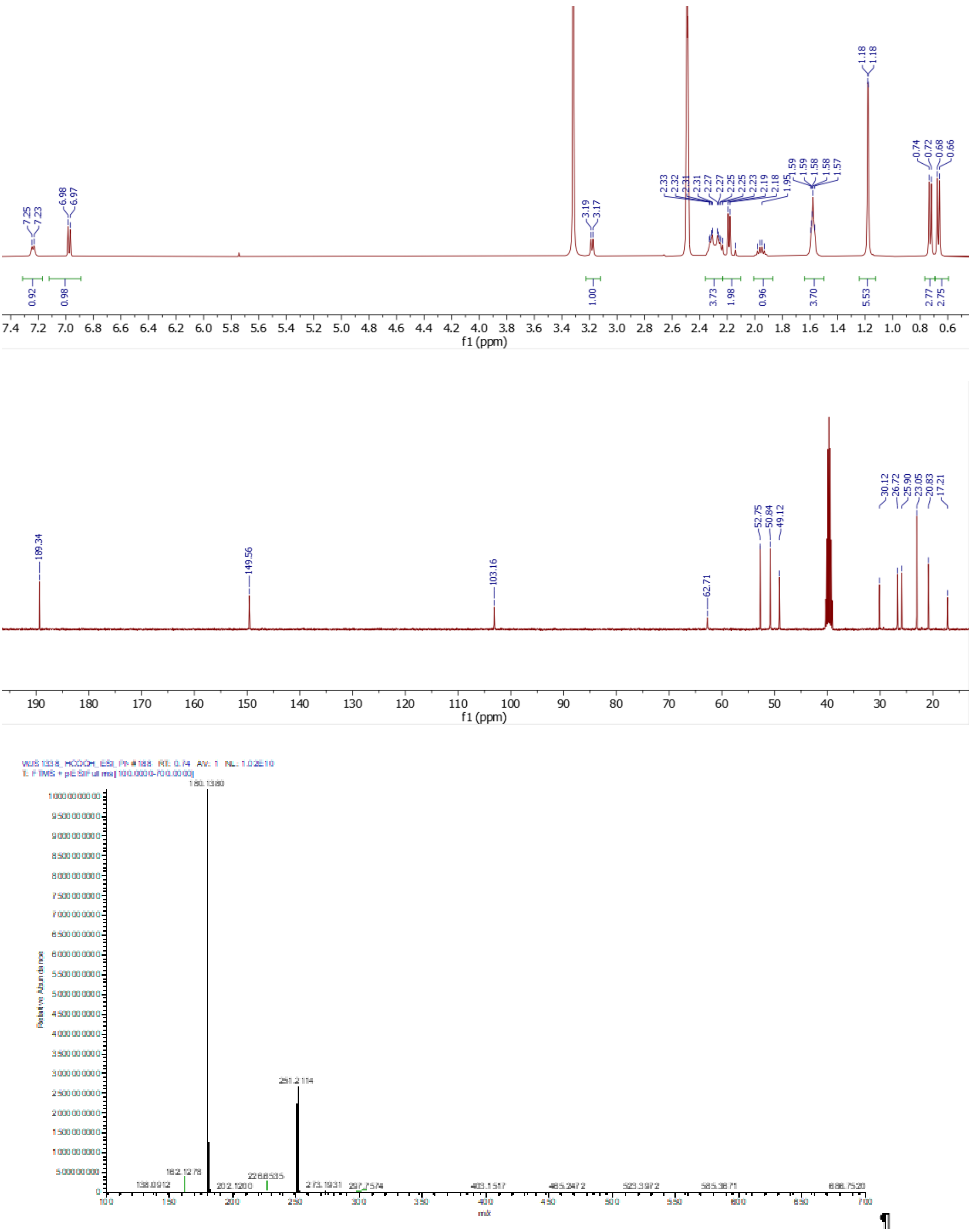

Figure S18. ^{1}H NMR at 400 MHz, ^{13}C NMR at 100 MHz spectra, and MS spectra for compound **11**

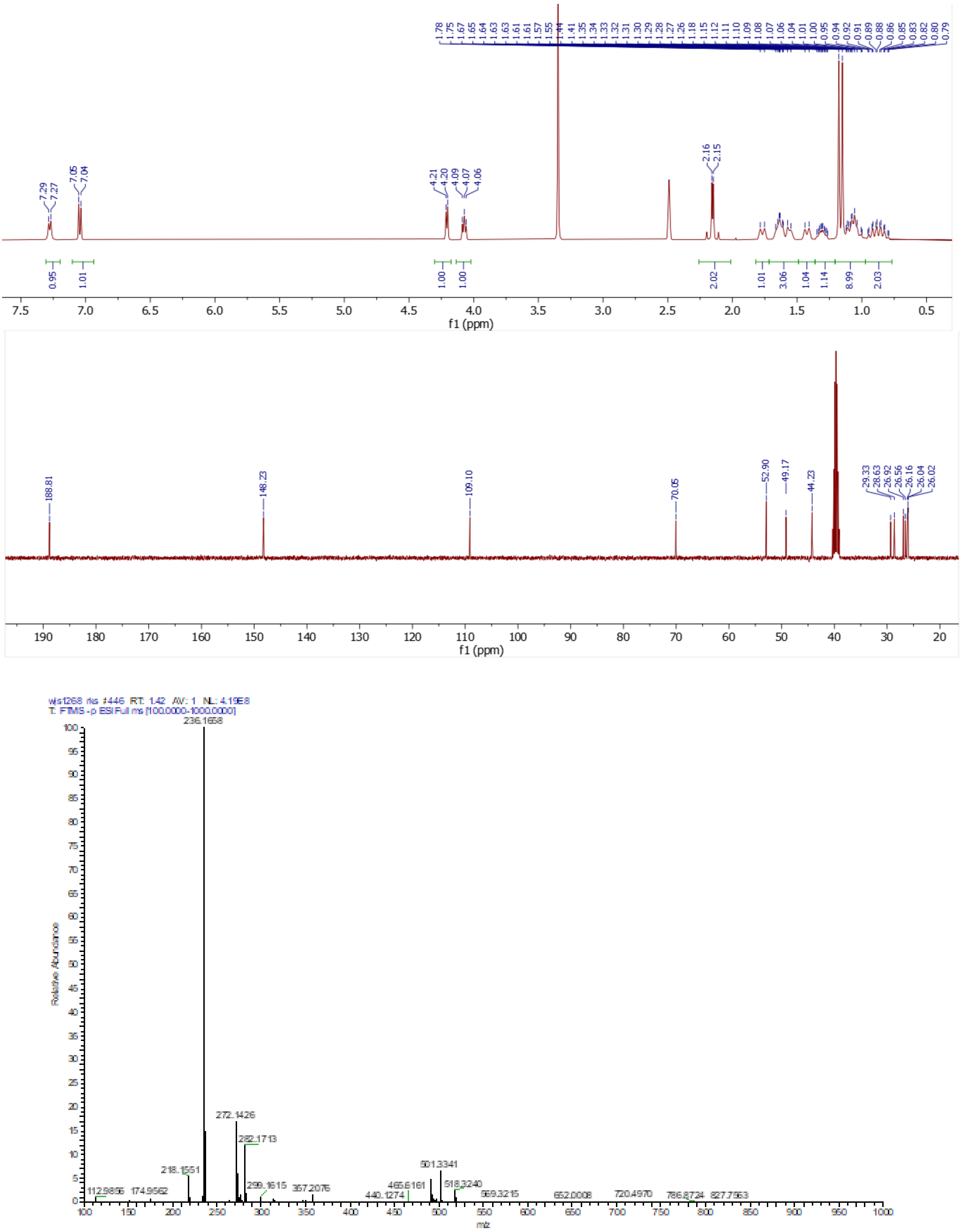

Figure S19. ^{1}H NMR at 400 MHz, ^{13}C NMR at 100 MHz spectra, and MS spectra for compound **12**

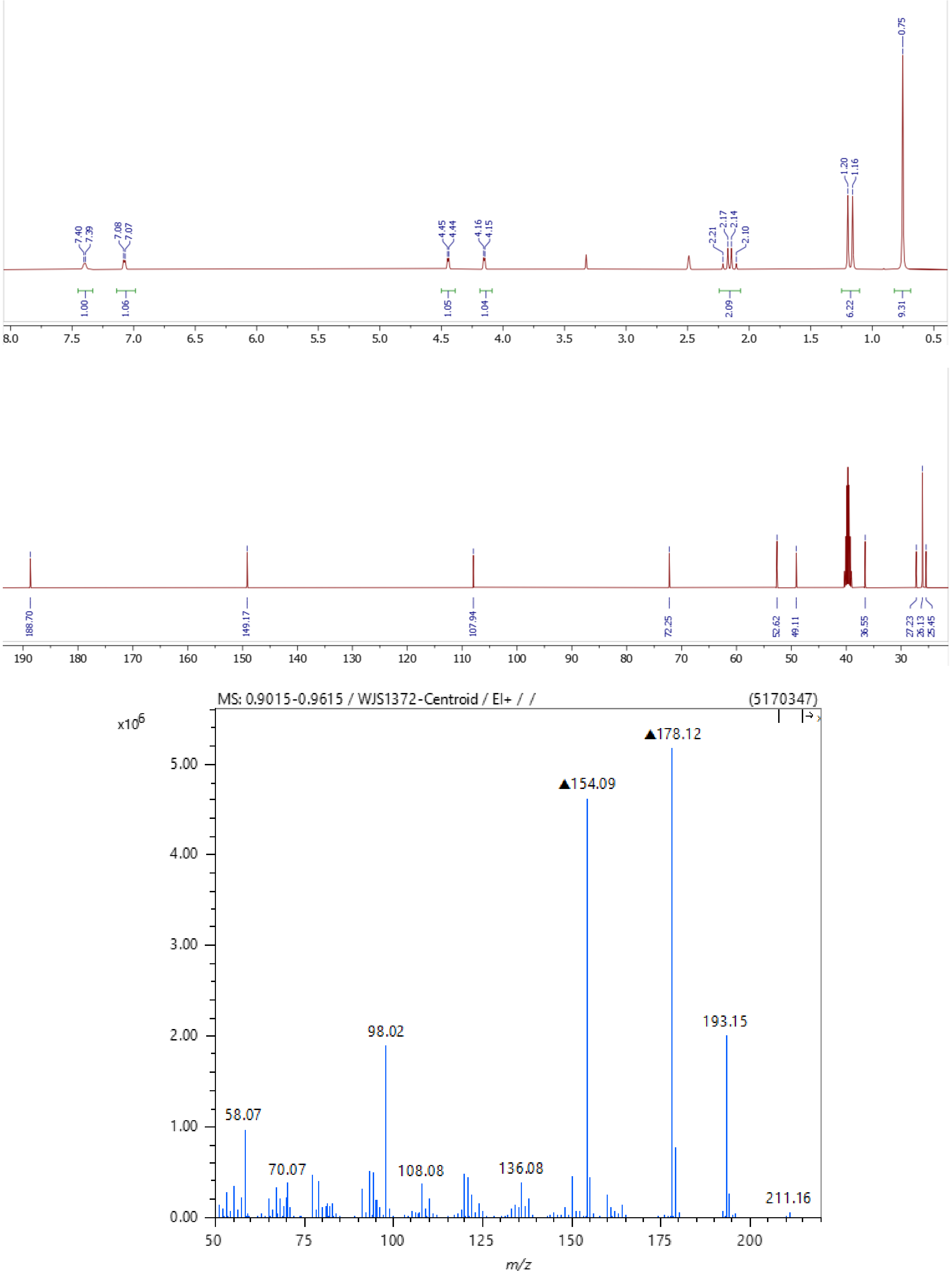

Figure S20. ^{1}H NMR at 400 MHz, ^{13}C NMR at 100 MHz spectra, and MS spectra for compound **13**

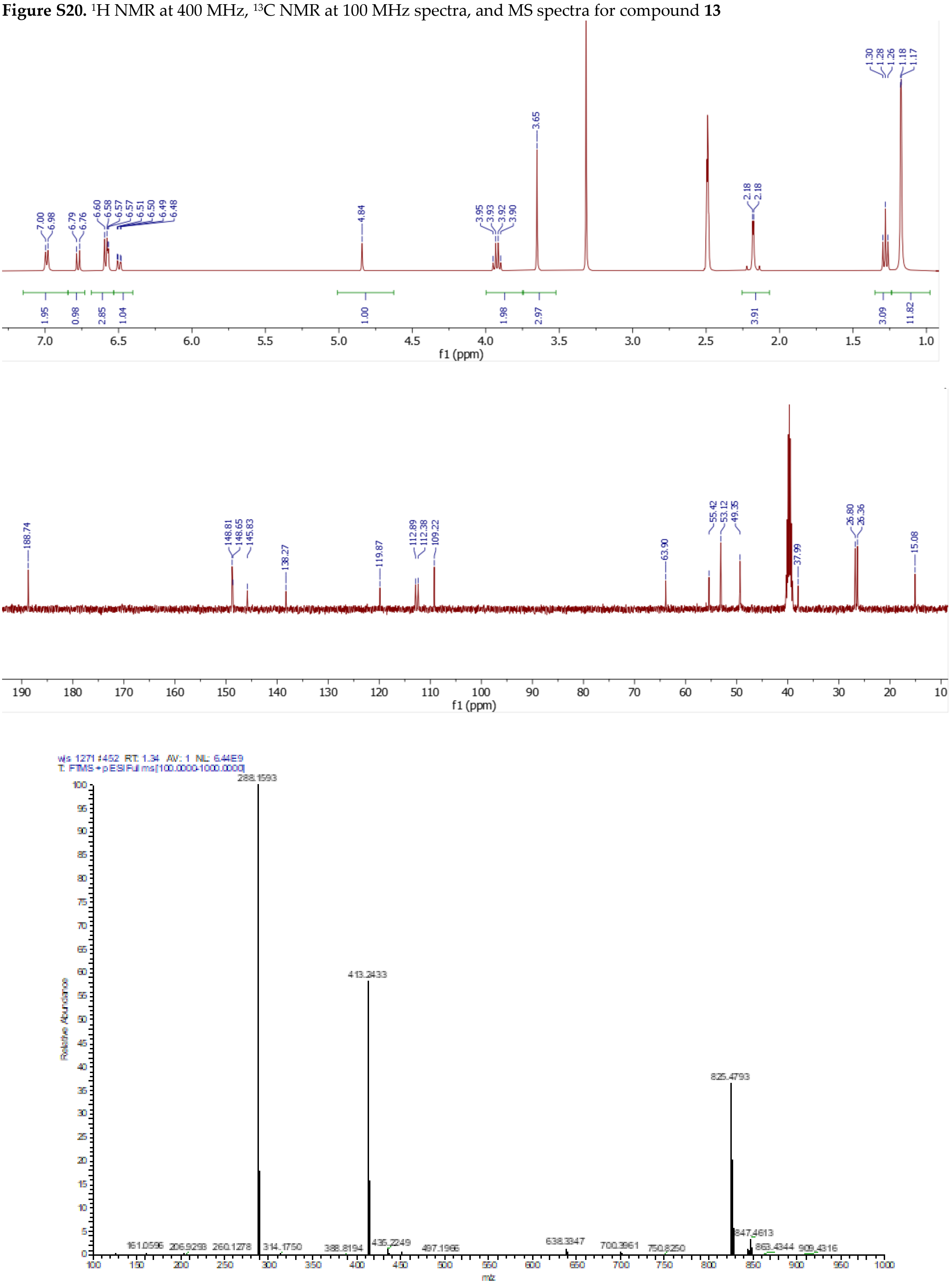

Figure S21. ^{1}H NMR at 400 MHz, ^{13}C NMR at 100 MHz spectra, and MS spectra for compound **14**

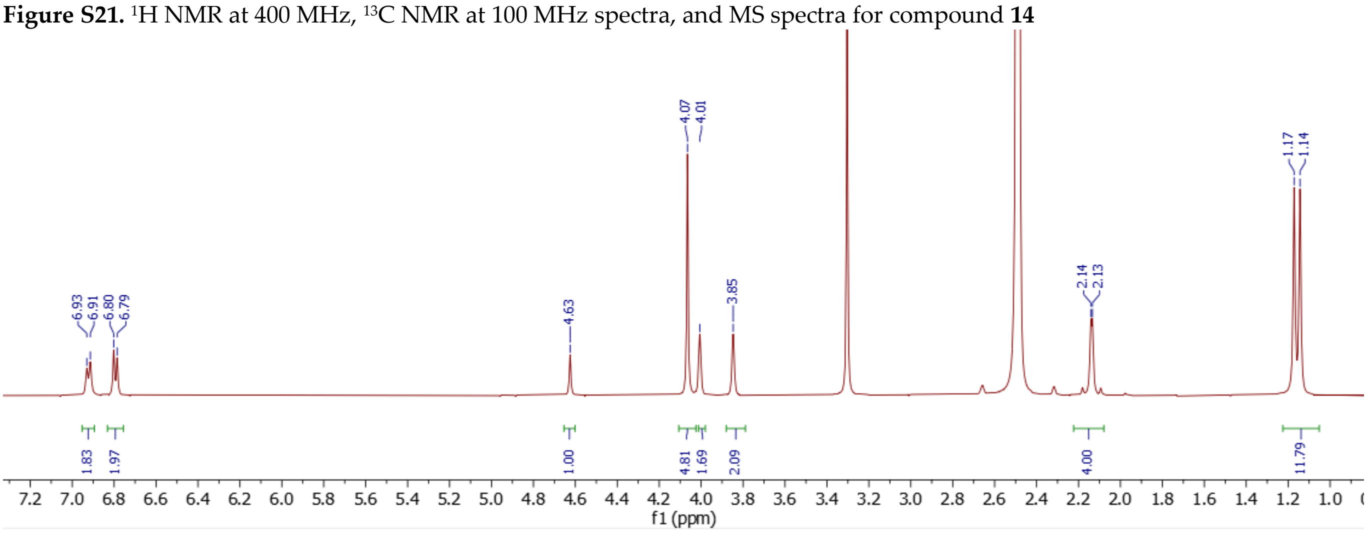

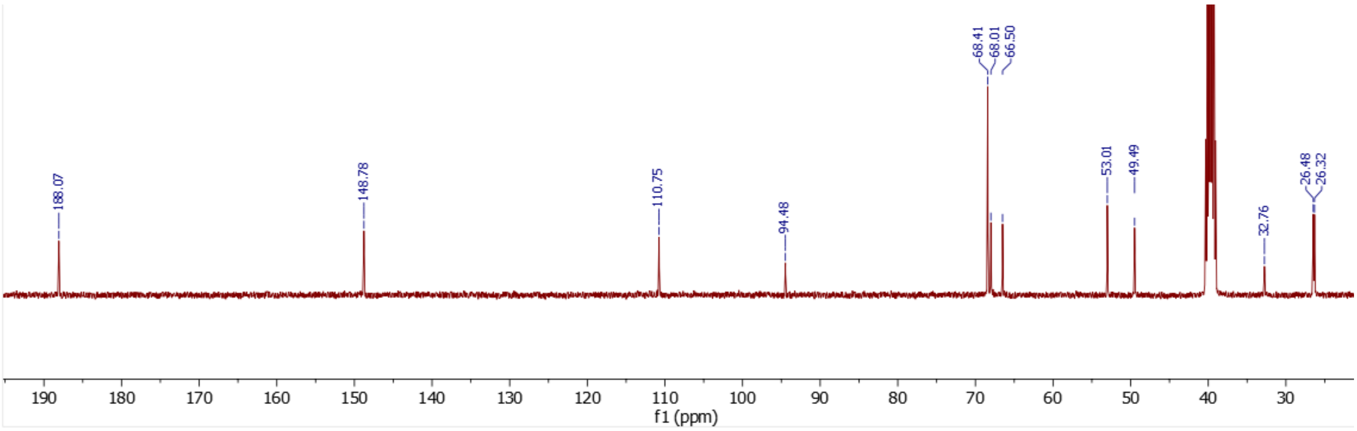

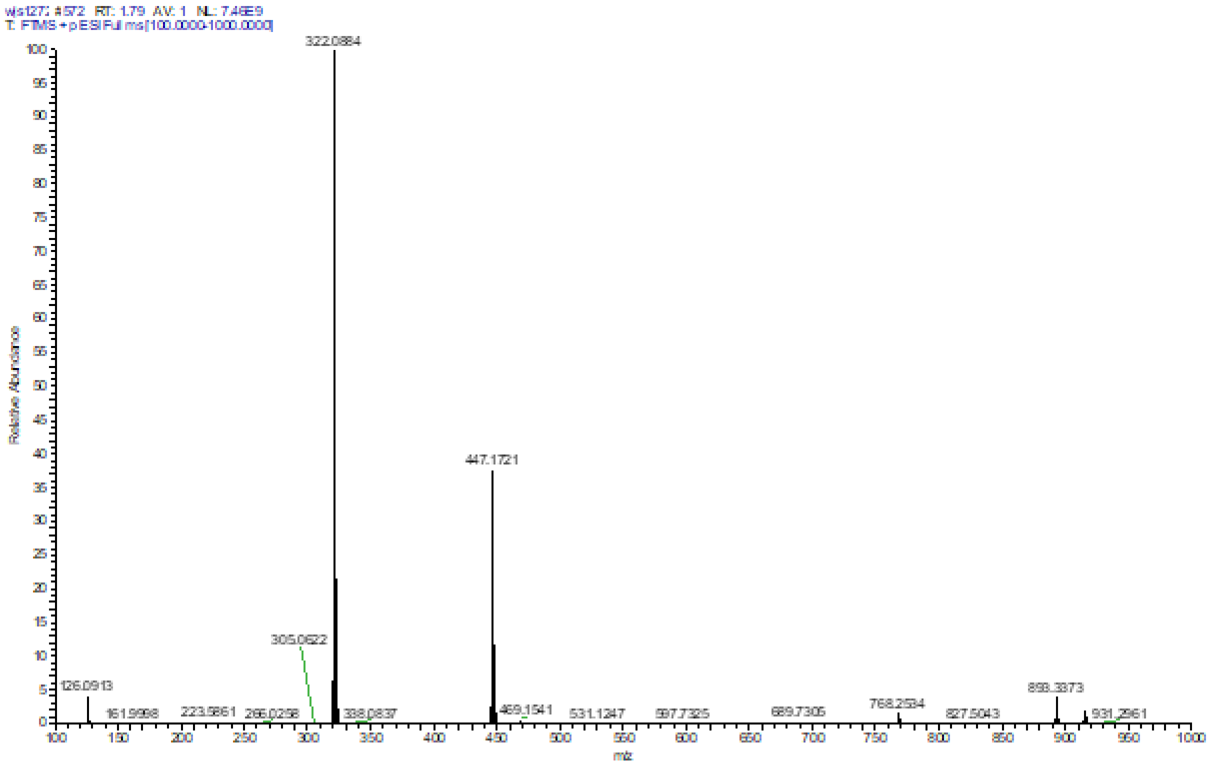

Table 1. Crystal data and structure refinement for **6a**.

Crystal data	
Identification code	WJS1268
Empirical formula	$C_{18}H_{30}N_2O$
Formula weight	290.44
Crystal description	block, colourless
Crystal size	0.23 x 0.15 x 0.13mm
Crystal system, space group	orthorhombic, P b c a
Unit cell dimensions:	a 10.5346(4)Å
b	11.7374(4)Å
c	28.1853(11)Å
Volume	3485.1(2)Å^3
Z	8
Calculated density	1.107Mg/m^3
F(000)	1280
Linear absorption coefficient μ	0.068mm^{-1}
Absorption correction	semi-empirical from equivalents
Max. and min. transmission	0.746 and 0.589
Unit cell determination	2.41° < Θ < 29.91°
	9745 reflections used at 100K
Data collection	
Temperature	100K
Diffractometer	Bruker APEX-II CCD
Radiation source	Incoatec microfocus sealed tube
Radiation and wavelength	MoK_α, 0.71073Å
Monochromator	multilayer monochromator
Scan type	ϕ and ω scans
Θ range for data collection	2.41 to 30.00°
Reflections collected / unique	125061 / 5084
Significant unique reflections	4213 with I > 2σ(I)
R(int), R(sigma)	0.0642, 0.0247
Completeness to Θ = 30.0°	99.9%
Refinement	
Refinement method	Full-matrix least-squares on F^2
Data / parameters / restraints	5084 / 211 / 1
Goodness-of-fit on F^2	1.035
Final R indices [I > 2σ(I)]	R1 = 0.0431, wR2 = 0.1082
R indices (all data)	R1 = 0.0545, wR2 = 0.1167
Extinction expression	none
Weighting scheme	$w = 1/[\sigma^2(F_o^2)+(aP)^2+bP]$ where $P = (F_o^2+2F_c^2)/3$
Weighting scheme parameters a, b	0.0543, 1.4314
Largest Δ/σ in last cycle	0.001
Largest difference peak and hole	0.417 and -0.219e/Å^3
Structure Solution Program	SHELXS-97 (Sheldrick, 2008)
Structure Refinement Program	SHELXL-2014/6 (Sheldrick, 2015)

Table 2. Selected bond lengths [Å] and angles [°] for **6a**.

N1-C2	1.4682(12)
N1-C6	1.3332(12)
C2-C3	1.5295(13)
C3-C4	1.5246(13)
C4-O4	1.2458(11)
C4-C5	1.4258(12)
C5-C6	1.3780(12)
C5-C9	1.5151(12)
C9-N11	1.4782(12)
N11-C12	1.4682(12)
N11-C15	1.4711(12)
C2-N1-C6	120.23(8)
C2-N1-H1	120.5(4)
C6-N1-H1	117.6(5)
O4-C4-C5	123.97(8)
O4-C4-C3	119.92(8)
C5-C4-C3	115.93(8)
C6-C5-C4	118.29(8)
C6-C5-C9	120.68(8)
C4-C5-C9	120.94(8)
C12-N11-C15	103.35(8)
C12-N11-C9	114.00(8)
C15-N11-C9	111.11(7)
O4-C4-C5-C6	178.86(8)
C3-C4-C5-C6	3.78(12)
O4-C4-C5-C9	2.21(13)
C3-C4-C5-C9	-172.87(8)
C4-C5-C6-N1	9.01(14)
C9-C5-C6-N1	-174.33(8)

Table 3. Hydrogen bond for **6a** [Å, °].

D-H···A	d(D-H)	d(H···A)	d(D···A)	<(DHA)
N1-H1···O4[i)]	0.88	1.9071(16)	2.7754(10)	168.7(6)

Symmetry transformations used to generate equivalent atoms:

[i)] 3/2-x, y-1/2, z

Table 4. Atomic coordinates and equivalent isotropic displacement parameters (Å^2) for **6a**. U_{eq} is defined as one third of the trace of the orthogonalized U_{ij} tensor.

	x	y	z	U_{eq}
N1	0.80786(8)	0.59527(7)	0.57590(3)	0.01582(17)
C2	0.87110(9)	0.70462(8)	0.56689(3)	0.01519(18)
C3	0.77025(9)	0.78680(8)	0.54810(3)	0.01419(18)
C4	0.64934(8)	0.79051(8)	0.57779(3)	0.01204(17)
O4	0.58493(6)	0.87944(6)	0.57938(2)	0.01720(15)
C5	0.61254(8)	0.68579(7)	0.59943(3)	0.01195(17)
C6	0.69135(9)	0.59289(8)	0.59435(3)	0.01350(17)
C7	0.97509(10)	0.68505(10)	0.53006(4)	0.0244(2)
C8	0.92818(10)	0.74893(10)	0.61347(4)	0.0217(2)
C9	0.48665(8)	0.67494(8)	0.62502(3)	0.01250(17)
N11	0.42549(7)	0.56605(7)	0.61192(3)	0.01451(16)
C12	0.30326(9)	0.54614(10)	0.63555(4)	0.0231(2)
C13	0.24392(11)	0.44916(10)	0.60675(5)	0.0303(3)
C14	0.29471(10)	0.46833(10)	0.55600(4)	0.0271(2)
C15	0.39252(10)	0.56394(9)	0.56121(4)	0.0223(2)
C21	0.50148(9)	0.68812(9)	0.67934(3)	0.01639(19)
C22	0.58155(10)	0.59488(10)	0.70246(4)	0.0222(2)
C23	0.58787(13)	0.61102(12)	0.75640(4)	0.0332(3)
C24	0.64059(12)	0.72816(12)	0.76873(4)	0.0321(3)
C25	0.56294(12)	0.82213(11)	0.74576(4)	0.0299(3)
C26	0.55433(11)	0.80582(9)	0.69198(4)	0.0219(2)

Table 5. Hydrogen coordinates and isotropic displacement parameters (Å^2) for **6a**.

	x	y	z	U_{iso}
H1	0.8516(5)	0.5314(3)	0.5760(5)	0.028(4)
H31	0.8070	0.8644	0.5468	0.025(2)
H32	0.7478	0.7644	0.5153	0.025(2)
H6	0.6601	0.5213	0.6048	0.017(3)
H71	1.0374	0.6308	0.5426	0.034(2)
H72	1.0173	0.7575	0.5230	0.034(2)
H73	0.9374	0.6543	0.5010	0.034(2)
H81	0.8607	0.7579	0.6371	0.033(2)
H82	0.9690	0.8227	0.6079	0.033(2)
H83	0.9913	0.6945	0.6252	0.033(2)
H9	0.4306	0.7380	0.6137	0.017(3)
H121	0.2494	0.6152	0.6345	0.031(3)
H122	0.3158	0.5234	0.6690	0.031(3)
H131	0.1501	0.4536	0.6075	0.046(3)
H132	0.2708	0.3740	0.6192	0.046(3)
H141	0.3349	0.3982	0.5435	0.041(3)
H142	0.2254	0.4914	0.5343	0.041(3)
H151	0.4686	0.5480	0.5417	0.037(3)
H152	0.3559	0.6378	0.5512	0.037(3)
H21	0.4145	0.6831	0.6934	0.024(3)
H221	0.6685	0.5968	0.6891	0.028(2)
H222	0.5442	0.5195	0.6952	0.028(2)
H231	0.5018	0.6027	0.7701	0.044(3)
H232	0.6428	0.5514	0.7704	0.044(3)
H241	0.6398	0.7383	0.8036	0.038(3)
H242	0.7297	0.7335	0.7578	0.038(3)
H251	0.6025	0.8968	0.7527	0.041(3)
H252	0.4764	0.8223	0.7595	0.041(3)
H261	0.6398	0.8146	0.6779	0.029(3)
H262	0.4987	0.8654	0.6783	0.029(3)

Table 6. Anisotropic displacement parameters (Å^2) for **6a**. The anisotropic displacement factor exponent takes the form: $-2\pi^2[h^2 a^{*2}U_{11} + ... + 2 h k a^* b^* U_{12}]$.

	U_{11}	U_{22}	U_{33}	U_{23}	U_{13}	U_{12}
N1	0.0144(4)	0.0108(4)	0.0223(4)	0.0003(3)	0.0049(3)	0.0023(3)
C2	0.0124(4)	0.0142(4)	0.0190(4)	-0.0002(3)	0.0030(3)	-0.0010(3)
C3	0.0143(4)	0.0128(4)	0.0154(4)	0.0011(3)	0.0014(3)	-0.0024(3)
C4	0.0118(4)	0.0113(4)	0.0130(4)	-0.0006(3)	-0.0019(3)	-0.0015(3)
O4	0.0162(3)	0.0106(3)	0.0248(3)	0.0012(3)	-0.0019(3)	0.0005(3)
C5	0.0119(4)	0.0110(4)	0.0129(4)	0.0001(3)	0.0011(3)	-0.0005(3)
C6	0.0149(4)	0.0104(4)	0.0153(4)	0.0006(3)	0.0021(3)	-0.0009(3)
C7	0.0179(5)	0.0249(5)	0.0306(5)	-0.0012(4)	0.0111(4)	-0.0012(4)
C8	0.0163(4)	0.0252(5)	0.0236(5)	-0.0018(4)	-0.0043(4)	-0.0003(4)
C9	0.0106(4)	0.0123(4)	0.0146(4)	-0.0001(3)	0.0009(3)	-0.0001(3)
N11	0.0113(3)	0.0151(4)	0.0172(4)	0.0006(3)	0.0002(3)	-0.0035(3)
C12	0.0130(4)	0.0290(5)	0.0272(5)	0.0039(4)	0.0028(4)	-0.0056(4)
C13	0.0183(5)	0.0260(6)	0.0465(7)	0.0038(5)	-0.0044(5)	-0.0095(4)
C14	0.0192(5)	0.0236(5)	0.0384(6)	-0.0101(5)	-0.0066(4)	-0.0030(4)
C15	0.0245(5)	0.0244(5)	0.0181(4)	-0.0019(4)	-0.0039(4)	-0.0082(4)
C21	0.0132(4)	0.0225(5)	0.0134(4)	-0.0020(3)	0.0018(3)	0.0005(3)
C22	0.0254(5)	0.0249(5)	0.0162(4)	0.0048(4)	-0.0016(4)	-0.0008(4)
C23	0.0396(7)	0.0440(7)	0.0162(5)	0.0074(5)	-0.0044(4)	-0.0046(6)
C24	0.0312(6)	0.0486(8)	0.0165(5)	-0.0028(5)	-0.0050(4)	-0.0022(5)
C25	0.0282(6)	0.0407(7)	0.0209(5)	-0.0132(5)	-0.0023(4)	0.0027(5)
C26	0.0243(5)	0.0225(5)	0.0191(4)	-0.0063(4)	-0.0034(4)	0.0038(4)

Table 7. Full list of bond lengths [Å] and angles [°] for **6a**.

N1-C2	1.4682(12)
N1-C6	1.3332(12)
N1-H1	0.88
C2-C3	1.5295(13)
C2-C7	1.5266(13)
C2-C8	1.5350(14)
C3-C4	1.5246(13)
C3-H31	0.99
C3-H32	0.99
C4-O4	1.2458(11)
C4-C5	1.4258(12)
C5-C6	1.3780(12)
C5-C9	1.5151(12)
C6-H6	0.95
C7-H71	0.98
C7-H72	0.98
C7-H73	0.98
C8-H81	0.98
C8-H82	0.98
C8-H83	0.98
C9-N11	1.4782(12)
C9-C21	1.5467(12)
C9-H9	1.00
N11-C12	1.4682(12)
N11-C15	1.4711(12)
C12-C13	1.5314(16)
C12-H121	0.99
C12-H122	0.99
C13-C14	1.5436(18)
C13-H131	0.99
C13-H132	0.99
C14-C15	1.5306(15)
C14-H141	0.99
C14-H142	0.99
C15-H151	0.99
C15-H152	0.99
C21-C22	1.5278(14)
C21-C26	1.5315(14)
C21-H21	1.00
C22-C23	1.5335(15)
C22-H221	0.99
C22-H222	0.99
C23-C24	1.5229(19)
C23-H231	0.99
C23-H232	0.99
C24-C25	1.5182(18)
C24-H241	0.99
C24-H242	0.99
C25-C26	1.5305(15)
C25-H251	0.99
C25-H252	0.99
C26-H261	0.99
C26-H262	0.99

C2-N1-C6	120.23(8)
C2-N1-H1	120.5(4)
C6-N1-H1	117.6(5)
N1-C2-C7	108.17(8)
N1-C2-C3	107.22(7)
C7-C2-C3	110.97(8)
N1-C2-C8	109.02(8)
C7-C2-C8	110.58(8)
C3-C2-C8	110.77(8)
C4-C3-C2	114.10(7)
C4-C3-H31	108.7
C2-C3-H31	108.7
C4-C3-H32	108.7
C2-C3-H32	108.7
H31-C3-H32	107.6
O4-C4-C5	123.97(8)
O4-C4-C3	119.92(8)
C5-C4-C3	115.93(8)
C6-C5-C4	118.29(8)
C6-C5-C9	120.68(8)
C4-C5-C9	120.94(8)
N1-C6-C5	125.35(8)
N1-C6-H6	117.3
C5-C6-H6	117.3
C2-C7-H71	109.5
C2-C7-H72	109.5
H71-C7-H72	109.5
C2-C7-H73	109.5
H71-C7-H73	109.5
H72-C7-H73	109.5
C2-C8-H81	109.5
C2-C8-H82	109.5
H81-C8-H82	109.5
C2-C8-H83	109.5
H81-C8-H83	109.5
H82-C8-H83	109.5
N11-C9-C5	109.59(7)
N11-C9-C21	112.20(7)
C5-C9-C21	112.00(7)
N11-C9-H9	107.6
C5-C9-H9	107.6
C21-C9-H9	107.6
C12-N11-C15	103.35(8)
C12-N11-C9	114.00(8)

C15-N11-C9	111.11(7)
N11-C12-C13	103.65(9)
N11-C12-H121	111.0
C13-C12-H121	111.0
N11-C12-H122	111.0
C13-C12-H122	111.0
H121-C12-H122	109.0
C12-C13-C14	103.96(9)
C12-C13-H131	111.0
C14-C13-H131	111.0
C12-C13-H132	111.0
C14-C13-H132	111.0
H131-C13-H132	109.0
C15-C14-C13	104.56(9)
C15-C14-H141	110.8
C13-C14-H141	110.8
C15-C14-H142	110.8
C13-C14-H142	110.8
H141-C14-H142	108.9
N11-C15-C14	105.34(8)
N11-C15-H151	110.7
C14-C15-H151	110.7
N11-C15-H152	110.7
C14-C15-H152	110.7
H151-C15-H152	108.8
C22-C21-C26	110.26(8)
C22-C21-C9	113.97(8)
C26-C21-C9	110.93(8)
C22-C21-H21	107.1
C26-C21-H21	107.1
C9-C21-H21	107.1
C21-C22-C23	111.00(9)
C21-C22-H221	109.4
C23-C22-H221	109.4
C21-C22-H222	109.4
C23-C22-H222	109.4
H221-C22-H222	108.0
C24-C23-C22	110.71(10)
C24-C23-H231	109.5
C22-C23-H231	109.5
C24-C23-H232	109.5
C22-C23-H232	109.5
H231-C23-H232	108.1
C25-C24-C23	111.23(10)
C25-C24-H241	109.4
C23-C24-H241	109.4
C25-C24-H242	109.4
C23-C24-H242	109.4
H241-C24-H242	108.0
C24-C25-C26	111.31(9)
C24-C25-H251	109.4
C26-C25-H251	109.4
C24-C25-H252	109.4
C26-C25-H252	109.4
H251-C25-H252	108.0
C25-C26-C21	111.39(9)
C25-C26-H261	109.3
C21-C26-H261	109.3
C25-C26-H262	109.3
C21-C26-H262	109.3
H261-C26-H262	108.0

C6-N1-C2-C7	-158.85(9)
C6-N1-C2-C3	-39.11(11)
C6-N1-C2-C8	80.85(11)
N1-C2-C3-C4	49.19(10)
C7-C2-C3-C4	167.11(8)
C8-C2-C3-C4	-69.66(10)
C2-C3-C4-O4	150.80(8)
C2-C3-C4-C5	-33.91(11)
O4-C4-C5-C6	178.86(8)
C3-C4-C5-C6	3.78(12)
O4-C4-C5-C9	2.21(13)
C3-C4-C5-C9	-172.87(8)
C2-N1-C6-C5	11.00(14)
C4-C5-C6-N1	9.01(14)
C9-C5-C6-N1	-174.33(8)
C6-C5-C9-N11	-40.22(11)
C4-C5-C9-N11	136.35(8)
C6-C5-C9-C21	84.97(10)
C4-C5-C9-C21	-98.46(10)
C5-C9-N11-C12	179.01(8)
C21-C9-N11-C12	53.93(10)
C5-C9-N11-C15	-64.71(10)
C21-C9-N11-C15	170.21(8)
C15-N11-C12-C13	44.16(10)
C9-N11-C12-C13	164.88(8)
N11-C12-C13-C14	-31.48(11)
C12-C13-C14-C15	7.50(12)
C12-N11-C15-C14	-39.44(11)
C9-N11-C15-C14	-162.10(8)
C13-C14-C15-N11	19.02(11)
N11-C9-C21-C22	59.79(10)
C5-C9-C21-C22	-63.95(10)
N11-C9-C21-C26	-175.05(8)
C5-C9-C21-C26	61.21(10)
C26-C21-C22-C23	56.61(11)
C9-C21-C22-C23	-177.88(9)
C21-C22-C23-C24	-57.07(13)
C22-C23-C24-C25	56.25(14)
C23-C24-C25-C26	-55.48(13)
C24-C25-C26-C21	55.38(13)

C22-C21-C26-C25	-55.73(11)
C9-C21-C26-C25	177.05(8)
